# Environmental evolution of a coastal lake in the Larsemann Hills, East Antarctica during the Holocene: a multi-proxy perspective

**DOI:** 10.1038/s41598-026-39218-8

**Published:** 2026-02-15

**Authors:** G. S. Joju, Anish Kumar Warrier, B. S. Mahesh, Avirajsinh Jadav, Cheryl A. Noronha-D’Mello, Masud Kawsar, M. C. Manoj, A. S. Yamuna Sali, Gokul Valsan, A. A. Krishnaprasad, Shardool Kokare, K. Balakrishna, Rahul Mohan

**Affiliations:** 1 Centre for Climate Studies, Manipal Institute of Technology, Manipal Academy of Higher Education, Manipal, 576104 Karnataka India; 2 Antarctic Science Division, National Centre for Polar and Ocean Research, Headland Sada, Vasco-da-Gama, 403804 Goa India; 3 Indian Institute of Tropical Meteorology, Ministry of Earth Sciences, Pune, 411008 India; 4 Birbal Sahni Institute of Palaeosciences, 53 University Road, Lucknow, 226007 Uttar Pradesh India; 5 Academy of Scientific and Innovative Research (AcSIR), Ghaziabad, 201002 India; 6 Department of Sciences, Manipal Institute of Technology, Manipal Academy of Higher Education, Manipal, 576104 Karnataka India; 7 Centre for Smart Coastal Sustainability, Manipal Institute of Technology, Manipal Academy of Higher Education, Manipal, 576104 Karnataka India

**Keywords:** Environmental sciences, Limnology, Ocean sciences

## Abstract

Coastal lakes in Antarctica provide exceptional archives of past climatic and environmental change. Their evolution is closely linked to variations in relative sea level (RSL) driven by deglaciation, making them ideal natural laboratories for investigating marine-lacustrine transitions and reconstructing ancient sea levels. This study presents a high-resolution reconstruction of mid- to late-Holocene paleoenvironmental changes from Heart Lake, a low-elevation coastal lake in the Larsemann Hills, East Antarctica. Multiple proxies, including diatom assemblages, environmental magnetic parameters, geochemical indicators, and sedimentological features, were employed to decipher the regional paleoclimate and environmental history. The lake existed as a submarine basin from approximately 6.37 to 3.07 cal ka BP. Around 4.3 cal ka BP, increasing chemical weathering indices suggest a trend toward warmer conditions in the lake. The first appearance of lacustrine diatoms around 3.07 cal ka BP marks the onset of environmental transition within the basin. By ~ 1.75 cal ka BP, the lake had become an isolated freshwater system. This shift from a marine to a lacustrine environment was likely driven by a post-glacial isostatic rebound and the consequent uplift of the land surface.

**Supplementary Information:**

The online version contains supplementary material available at 10.1038/s41598-026-39218-8.

## Introduction

The East Antarctic Ice Sheet (EAIS), the largest on the planet, has the potential to raise the global sea level by around 53 metres^1,2^. Any changes in its volume must, therefore, be monitored closely since it has the potential to impact on the mean sea levels around the world. Understanding the regional deglacial history and the variables controlling ice glacial behavior is crucial in this regard^3^. There are many unknowns about the extent of the EAIS after the last glacial maximum (LGM), particularly during the Holocene. Paleoclimate studies focusing on the Holocene can shed some light on the behavior and extent of the EAIS during this period. Archives such as ice cores, marine sediments, and lake sediments allow for the reconstruction of the past climate. Even though ice cores provide high-resolution climate records, their availability is limited in the coastal regions of Antarctica. Marine sediments, on the other hand, are influenced by numerous oceanic and terrestrial factors and, therefore, do not represent regional climatic variations accurately. Lake sediments can play a vital role in this scenario. As the lake sediments are sensitive to the environmental changes around them, they can be used as archives to reconstruct the paleoclimatic and paleoenvironmental variations on local to regional scales.

**Fig. 1:**
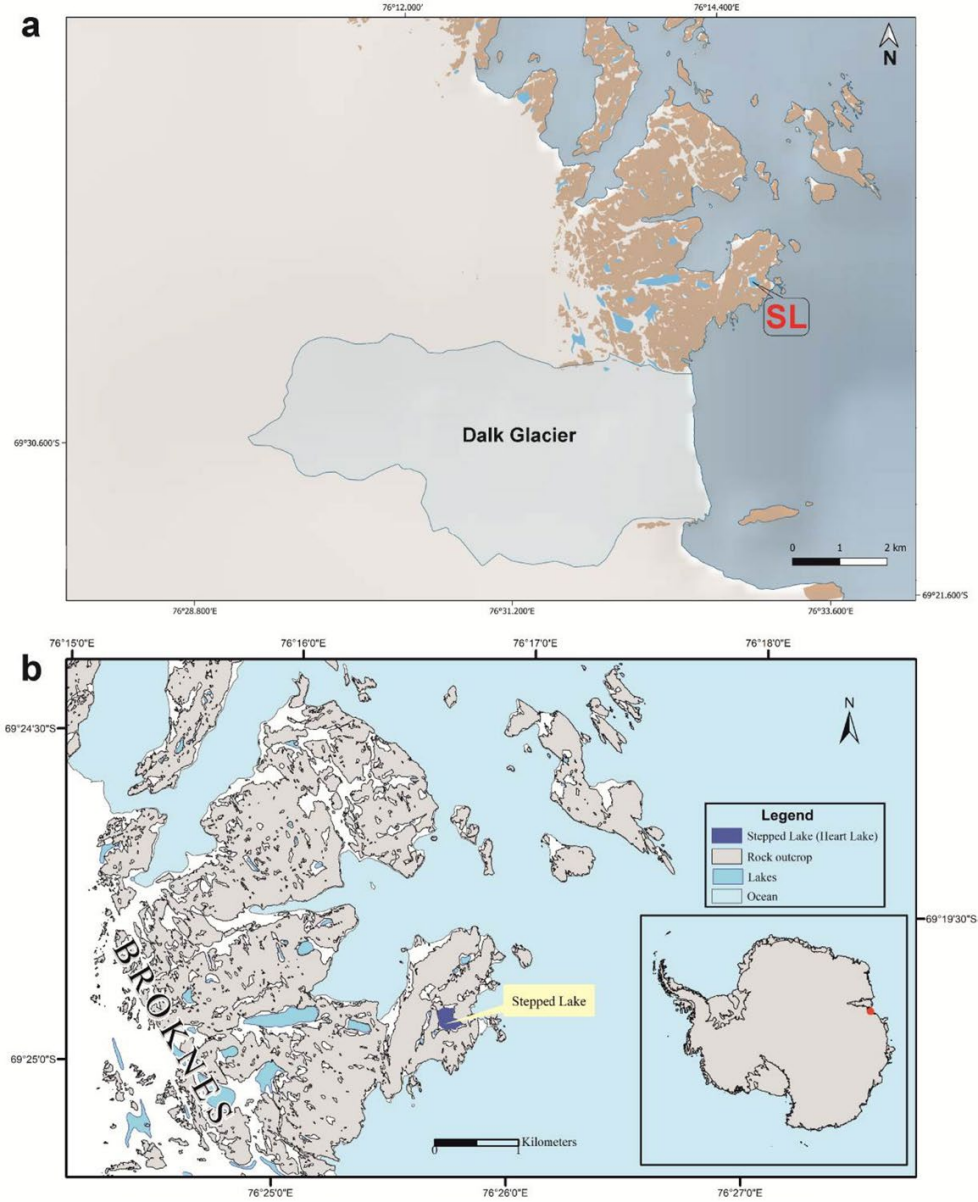
**(a)** Map showing location of Dalk Glacier^7^, generated using the software Quantarctica^7^ (version 3.2, https://npolar.no/quantarctica/); **(b)** Map of Larsemann Hills in East Antarctica showing the location of Heart Lake (modified after ref^8^. SL represents Stepped Lake (Heart Lake). This map was generated using the software ArcGIS (version 10.1, https://www.esri.com/en-us/arcgis/products/arcgis-desktop/overview).

Coastal lakes from Antarctica provide unique records of past climatic and environmental changes. The environmental conditions of these lakes are influenced by the variations in the relative sea level (RSL). The RSL is controlled by the rheological properties of the mantle, the volume of ice cover over the land, and the eustatic sea level (ESL)^4^. During times of high RSL, these lakes are susceptible to marine incursions due to their proximity to the coast and low elevation, while during periods of RSL low stand, the lake environment changes from marine to lacustrine. Thus, sedimentary records from these lakes can be used to reconstruct the past marine-lacustrine transitions and the deglacial history of the region. Heart Lake (also called Stepped Lake) is one such coastal lake in the Larsemann Hills, East Antarctica. The proximity of the lake to the coast (~ 200 m) makes it an ideal candidate to probe for evidence of such marine incursions during the RSL high stand (Fig. 1). Previous studies from Heart Lake revealed that until 2.95 cal ka BP, the lake was under marine influence, during which time marine and sea-ice diatoms dominated the assemblage^5^. Following this, a shift in environmental conditions was observed wherein lacustrine diatoms dominated the assemblage from 2.95 cal ka BP. No transition zone between marine and lacustrine conditions was reported earlier. Ref^6^. carried out organic geochemical and diatom analyses on a 135 cm long sediment core from the same lake. The authors observed the dominance of marine and sea-ice diatoms in the sedimentary record till 5 ka BP, followed by freshwater diatoms till the modern age. The observations were not consistent with the data reported in ref^5^. This could probably be due to updated radiocarbon measurements and calibration programs or different coring locations within the lake.

Most current studies on the coastal lakes are based on diatom studies and stable isotope studies on organic-rich sediments^5,6,9–11^. Studies based on diatom assemblage are an ideal tool for deciphering the past marine-lacustrine transitions. However, the environmental settings under which the lake existed (submarine basin or isolated basin), could have a major impact on the environmental variations. The lake basin could be affected by different processes, such as when it was a submarine basin and later, when it was an isolated basin. In this scenario, inorganic geochemistry and environmental magnetism provide insights into the reconstruction of lake settings, provenance, transportation and depositional history^12,13^. Environmental magnetic measurements have been previously applied to lacustrine sediments of Antarctica to reconstruct the climatic and environmental variations^12,14,15^. Even though there are several studies on the Holocene climate/environmental history of East Antarctica^16–22^, more studies are needed to improve the spatial coverage of the Antarctic environmental variations during the Holocene. In the current study, we have carried out multi-proxy analyses on a well-dated sediment core from Heart Lake to gain better insights into the past climate and environmental changes in the Larsemann Hills region of East Antarctica, during the past 6.37 ka.

## Study area

The Larsemann Hills lies along the Ingrid Christensen Coast of Princess Elizabeth Land. It is an ice-free oasis in East Antarctica located along 69°24’S and 76°20’E. There are two major (Stornes and Broknes Peninsula) and one minor (Mirror Peninsula) peninsula in the Larsemann Hills, along with several islands^23^. The peninsulas are enclosed by the Dalk Glacier to the southeast and the ice sheet towards the south. Larsemann Hills cover an area of around 50 square kilometers and has more than 150 lakes^24^. The air temperature during the austral summer is around 4 °C, while it ranges between − 15 °C and − 18 °C during the austral winter^25^. The region is distinguished by slightly undulant topography, consisting of low-rounded hills dotted with lakes of variable sizes and U-shaped glaciated valleys^26^. The main erosive agents in the Larsemann Hills are ice, water and salt^25^. The lakes in the region are partially ice-free during the austral summer, while they have an ice cover of up to 2 m thick during the austral winter^25^. Heart Lake is located around 200 m from the coast, 2.5 km from the Dalk Glacier on the east, and 2.6 km from the continental ice-sheet towards the south^5,24^ (Fig. 1a and b). The lake sill is at an elevation of ~ 5 m above sea level^5,6^. The geology around the lake is dominated by granites, gneisses and metapelites consisting of minerals such as garnet, magnetite, spinel, biotite and some cordierite^27^. Cyanobacteria in the form of microbial mats are present on the lake bed^5^. Previous studies indicate that structurally decayed microbial mats are present down the core in Heart Lake, overlying consolidated organic sediment, inorganic diamicton and consolidated sand and grit^25^.

## Materials and methods

### Sediment coring

The SL1 sediment core from Heart Lake, Larsemann Hills (69° 22’ 33.6"S, 76° 22’ 58.8"E), was obtained by means of a UWITEC piston corer at a water depth of ~ 4 m during the 33rd Indian Scientific Expedition to Antarctica (January 2014). The obtained sediment core had a length of 112.5 cm and was later transported to the land-based laboratory at the National Centre for Polar and Ocean Research, India. The subsampling of the sediment core was done at an interval of 0.5 cm.

### Chronology and lithology

Untreated bulk sedimentary organic matter from eight sections was sent for AMS radiocarbon dating at the University of Arizona, United States of America. The pre-treatment of the samples was carried out at the AMS facility and ^14^C was measured on acid-insoluble organic matter. The obtained ^14^C dates were converted into calibrated years (BP) using the program BACON^28^ (version 3.0.0) on the ‘R’ platform^29^ (version 4.2.0). SHCal20 curve^30^ and Marine20 curve^31^ was used for the calibration (Supplementary Table 1).

We assessed the diatom assemblages for downcore to delineate the marine-lacustrine transition. Permanent slides were prepared for the top 25 cm at 1 cm intervals and at 5 cm intervals from 30 cm to the core-bottom. Diatom analysis, sediment treatment and slide preparation followed the technique described in ref^32^. Based on previous literature^5,6,10,33^, the most abundant species (Supplementary Fig. 1), *Stauroforma inermis* (freshwater species) and *Fragilariopsis curta* (sea-ice indicator species), were assessed to identify the marine-lacustrine transitions (See Supplementary Note 1).

The ^14^C content of modern freshwater lakes in the Larsemann Hills is in near equilibrium with atmospheric CO_2_^25^. Therefore, no reservoir correction was applied to the upper section of the core (8 cm to the core-top), which represents a freshwater lake (see Sect. “Chronology and lithology”). These samples were calibrated using the SHCal20 calibration curve^30^. In contrast, diatom data indicate a marine influence in the lake from the core-bottom up to 9 cm depth. For all dated samples below this depth, a reservoir correction factor of 1300 years was applied^34,35^, and the ages were calibrated using the Marine20 calibration curve^29^. Based on the resulting age-depth model, sedimentation rates were subsequently calculated.

### Environmental magnetism

The environmental magnetic analysis of the samples was carried out at the “CSIR-National Institute of Oceanography,” Goa, and the “National Centre for Polar and Ocean Research,” Goa, following the standard protocols^36,37^. The magnetic susceptibility measurements were carried out on a Bartington MS2B dual frequency magnetic susceptibility meter at two frequencies: a low frequency of 0.465 kHz (χ_lf_) and a high frequency of 4.65 kHz (χ_hf_). The percentage frequency-dependent magnetic susceptibility (χ_fd_%) was calculated from χ_lf_ and χ_hf_^36^. Anhysteretic Remanent Magnetization (ARM) was applied to the samples with a peak alternating field of 100_mT_ and a DC field of 50_µT_. To calculate the susceptibility of ARM, the ARM was divided by the intensity of DC field^38^. Isothermal remanent magnetization (IRM) was applied by to the samples with an MMPM10 pulse magnetizer. IRM induced at a field of 1 T was taken as the “Saturation Isothermal Remanent Magnetization” (SIRM). Remanence induced in the samples was determined on an AGICO JR-6 A spinner magnetometer with a sensitivity of 2.4 × 10^− 6^ A/m. The L ratio was calculated using the equation “L ratio = (SIRM + IRM_−300mT_)/(SIRM + IRM_−100mT_)”^38,39^. Several inter-parametric ratios were plotted in order to ascertain the magnetic properties of the studied samples^36,38^.

### Geochemical analysis of sediment samples

Around 0.2 g of powdered sediment samples were digested in a mixture of concentrated HNO_3_, HCl, and HF following ref^40^. The samples were heated on a hotplate for complete digestion and filtered on 0.22 μm filter paper and finally made up to 50 ml using 0.2 N HNO_3_. The digested samples were analyzed on an ICP-OES (“Thermo Scientific iCAP 7000 Series”) at the “Environmental Research Laboratory, Manipal Institute of Technology”. Certified reference material BCR^®^–667 was also analyzed to ensure the quality of the analysis. Duplicate samples and check standards were used for the QA/QC checks. The RSD was found to be less than 5%.

Weathering indices were calculated to understand the paleoweathering. “Chemical index of alteration (CIA)”^41^ is commonly used to understand the rate of weathering. It is given as

1 $${\text{CIA = [A}}{{\mathrm{l}}_{\mathrm{2}}}{{\mathrm{O}}_{\mathrm{3}}}{\mathrm{/(A}}{{\mathrm{l}}_{\mathrm{2}}}{\mathrm{O3}}\,{\mathrm{+}}\,{\mathrm{CaO+N}}{{\mathrm{a}}_{\mathrm{2}}}{\mathrm{O+}}{{\mathrm{K}}_{\mathrm{2}}}{\text{O)] }}^*100$$

Values less than 50 for this index reflect fresh rock, values greater than 70 indicate moderate to strong weathering, while values near 100 suggest completely weathered rocks.

The “plagioclase index of alteration (PIA)”^42^ is an indicator of the grade of alteration of feldspars in the samples.

2 $${\text{PIA = [}}\left( {{\mathrm{A}}{{\mathrm{l}}_{\mathrm{2}}}{\mathrm{O3-}}{{\mathrm{K}}_{\mathrm{2}}}{\mathrm{O}}} \right){\mathrm{/(Al2O3}}\,{\mathrm{+}}\,{\mathrm{CaO+N}}{{\mathrm{a}}_{\mathrm{2}}}{\mathrm{O+}}{{\mathrm{K}}_{\mathrm{2}}}{\text{O)] }}^*100$$

Values less than 50 reflect fresh rocks, while values close to 100 indicate clay minerals. The “index of compositional variation (ICV)”^43^ is used to estimate the compositional maturity of the sediments. The formula is.

3 $${\mathrm{ICV=(F}}{{\mathrm{e}}_{\mathrm{2}}}{{\mathrm{O}}_{\mathrm{3}}}{\mathrm{+}}{{\mathrm{K}}_{\mathrm{2}}}{\mathrm{O+N}}{{\mathrm{a}}_{\mathrm{2}}}{\mathrm{O}}\,{\mathrm{+}}\,{\mathrm{CaO+MgO}}\,{\mathrm{+}}\,{\mathrm{MnO+Ti}}{{\mathrm{O}}_{\mathrm{2}}}{\mathrm{)/A}}{{\mathrm{l}}_{\mathrm{2}}}{{\mathrm{O}}_{\mathrm{3}}}$$

ICV values below 1 reflect compositionally mature sediments, whereas values greater than 1 reflect compositionally immature sediments.

### Loss-on-ignition analysis

Loss-on-ignition analysis was carried out to estimate the total organic carbon (TOC) content of the sediment samples^44^. Around 1 g of the sediments was weighed in silica crucibles and dried overnight at 105 °C. The crucibles were weighed after this step and placed at 550 °C in a muffle furnace for four hours to determine the TOC. The difference in the weight of the samples following this step gives the TOC content of the samples^44^. Dean’s formula was used to calculate the TOC content of the sediments^45,46^.

### Sediment particle size analysis, end member modelling and quartz grain studies

For particle size analysis, the samples were pretreated with H_2_O_2_ and HCl to remove the organic matter and carbonates, respectively. The samples were then centrifuged at 2500 rpm and analysed on a Laser Diffraction Particle Size Analyzer (Beckman Coulter LS*™* 13 320) at the Birbal Sahni Institute of Palaeosciences, Lucknow. End-member analysis (EMA) of the granulometric data was carried out using the program AnalySize^47^, a software package running on MATLAB (https://github.com/greigpaterson/AnalySize). For the present study, non-parametric end-member analysis was carried out on AnalySize v1.2.2. Based on the coefficient of determination (R^2^) and angular deviation (θ), a four end-member model was selected for the data (See Supplementary Note 2, Supplementary Fig. 2). The statistical analysis of the granulometric parameters was carried out using AnalySize and GRADISTAT^48^ (v. 9.1).

For quartz grain studies, about 2 g of the sediments were oven-dried and 10 ml of 30% H_2_O_2_ was added to eliminate the organic content. The clay cover and fine-grained particles attached to the quartz granules were removed by the addition of sodium hexametaphosphate to the samples after oven-drying. The samples were sieved on a 125 μm sieve, and the > 125 μm fraction was oven dried. Using a stereo zoom magnifying microscope (Nikon SMZ745), rounded quartz grains from the obtained fractions observed and counted, and the percentage of rounded quartz grains in each sample was calculated.

### Scanning electron microscopy and energy dispersive spectroscopic studies

Magnetic extracts from selected samples were subjected to SEM-EDS analysis to understand the morphology of the magnetic crystal. Magnetic minerals were extracted according to ref^49^. The extracted samples were observed under a Zeiss EVO MA18 Scanning Electron Microscope (SEM). The composition of the extracted particles was identified using an Oxford X-act Energy Dispersive Spectrometer (EDS).

### Statistical analysis

Principal component analysis of the dataset (environmental magnetic, geochemical and TOC data) was carried out on PAST^50^ (Version 4.03) in order to understand the control of paleoclimate on the environmental magnetic and elemental data of Heart Lake. Broken stick model^51,52^ was applied to identify the significant components in the dataset, accountable for the variance. Pearson’s correlation analysis (two-tailed) of the dataset was carried out on SPSS (v. 27.0.1.0).

## Results and discussion

### Chronology and lithology

The results from the diatom analysis (Supplementary Table 2) indicate that the top 8 cm of the sediment core is entirely populated by freshwater diatoms (*Stauroforma inermis*), indicating lacustrine conditions in the lake. Between 9 and 17 cm depth, a mixed assemblage of freshwater diatoms (*Stauroforma inermis*) and marine diatoms (*Fragilariopsis curta*) was observed, while from 18 cm to the bottom of the core, marine diatoms (*Fragilariopsis curta*) are dominant. This indicates that the section from 9 cm to 17 cm represents a transition zone between marine and lacustrine conditions, while from 18 cm to the core-bottom, the lake was under marine influence. Based on these results, the sediment core was divided into three zones, viz., zone 1 (112.5–18 cm: marine conditions/submarine basin), zone 2 (17–9 cm: transition zone) and zone 3 (8 cm to core-top: freshwater conditions). The diatom records indicate that Heart Lake underwent a transition from a submarine basin in Zone 1 to a freshwater lake in Zone 3.

As the catchment lacked terrestrial organic matter such as lichens or mosses, and the region contains no carbonate-bearing rocks, the lake basin chronology was not influenced by dead carbon. Furthermore, radiocarbon dates from different sections of the core showed no evidence of external terrestrial organic matter inputs or contributions from carbonate-bearing rocks (e.g., hard-water effects). The calibrated ages span from 0.25 cal ka BP to 6.37 cal ka BP (Supplementary Fig. 3). The temporal resolution of the ^14^C-dated sediment sequence is 56.58 years/cm. Sedimentation rates in Heart Lake range from 3.4 cm/ka to 58.9 cm/ka, with a mean of 34.9 cm/ka. The highest average sedimentation rate occurs in Zone 1 (39.6 cm/ka), decreasing to 7.0 cm/ka in Zone 2, and reaching the lowest value in Zone 3 (5.6 cm/ka).

The lithology of the core is dominated by muddy sand between 18 cm and 112.5 cm (Supplementary Fig. 3). Above this, between 9 cm and 17 cm, coarse sand and mud, along with compressed algal mats, are recorded. In the top 8 cm of the sediment core, cyanobacterial mats with coarse sand and silt are observed.

### Magnetic concentration

**Fig. 2:**
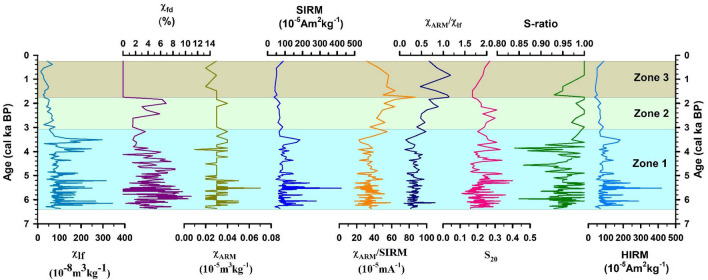
Downcore variations in the environmental magnetic properties of SL1 sediment core.

The downcore variations in the environmental magnetic parameters of Heart Lake are given in the Fig. 2. The parameter χ_lf_ acts as a proxy for the concentration of magnetic grains^36,37^. Higher values of magnetic susceptibility indicate higher concentrations of magnetic minerals. Low-frequency magnetic susceptibility (χ_lf_) values vary from 24.5 to 344.3 × 10^− 8^ m^3^ kg^− 1^ with a mean value (± S.D.) 96.1 (± 58.5) ×10^− 8^ m^3^ kg^− 1^ (Table 1). The parameter χ_fd_ is reflective of the intensity of pedogenesis, and χ_fd_% is a semi-quantitative indicator of the concentration of superparamagnetic (SP) grains in the samples^37,53^. χ_fd_% values less than 2 signifies the lack of superparamagnetic grains. Values between 2 and 10 are suggestive of a mixture of superparamagnetic and coarser grains, and values > 10 indicate the dominance of superparamagnetic grains^54^. Most samples show χ_fd_% between 2 and 10, indicating a mixture of SP grains and coarse grains, while few samples show values > 10 suggesting the predominance of SP grains in these samples (Table 1). χ_ARM_ acts as an indicator for the proportion of stable single domain (SSD) grains^55^. The samples show average χ_ARM_ values of 0.03 (± 0.01) ×10^− 5^ m^3^kg^− 1^ (Table 1). χ_ARM_ is also positively correlated with χ_lf_ (*r* = 0.61, *p* = 4.6 × 10^− 7^, *n* = 57), suggesting the influence of SSD-sized grains on the magnetic data of Heart Lake. The peak field IRM_1000mT_ was the saturation isothermal remanent magnetization (SIRM). The SIRM is reflective of the concentration of all the remanence-carrying magnetic minerals^56^. SIRM ranges from 36.6 × 10^− 5^ Am^2^kg^− 1^ to 423.4 × 10^− 5^ Am^2^kg^− 1^ with a mean value (± S.D.) of 93.5 (± 52.6) ×10^− 5^ Am^2^kg^− 1^ (Table 1). A statistically significant correlation is noted among χ_lf_ and SIRM (*r* = 0.79, *p* = 3.6 × 10^− 13^, *n* = 57; Supplementary Table 3), reflecting the control of magnetically strong ferrimagnets in the samples.

**Table 1:** Descriptive statistics of the environmental magnetic, geochemical and sedimentological parameters of the SL1 sediment core.

Parameter	Units	*n*	Mean	Median	Standard Deviation	Minimum	Maximum
χ_lf_	10^− 8^ m^3^ kg^− 1^	109	96.1	79.22	58.53	24.58	344.33
χ_fd_%	%	109	4.37	4.17	2.82	0.00	10.91
χ_ARM_	10^− 5^ m^3^ kg^− 1^	109	0.03	0.03	0.01	0.01	0.07
SIRM	10^− 5^ Am^2^ kg^− 1^	109	93.46	81.26	52.62	36.63	423.37
χ_ARM_/SIRM	10^− 5^ mA^− 1^	109	37.02	35.86	10.97	16.96	87.04
χ_ARM_/χ_lf_	Dimensionless	109	0.40	0.36	0.19	0.10	1.17
S_20_	Dimensionless	109	-	-	-	0.11	0.38
S-ratio	Dimensionless	109	-	-	-	0.84	1.00
HIRM	10^− 5^ Am^2^ kg^− 1^	109	91.57	79.18	52.32	36.63	418.87
L-ratio	Dimensionless	109	1.11	1.11	0.04	1.00	1.24
Ag	ppm	102	127.92	143.32	56.59	7.17	203.50
Al	ppm	102	40379.44	40184.68	8859.43	15569.95	58280.45
Ba	ppm	102	309.70	328.53	69.00	114.14	452.13
Ca	ppm	102	9852.82	9486.88	1973.85	6109.83	16093.30
Cd	ppm	102	2.71	2.74	1.33	0.33	6.69
Co	ppm	102	8.56	9.04	2.71	2.44	14.99
Cr	ppm	102	41.48	46.64	16.16	4.80	65.50
Cu	ppm	102	11.63	12.52	4.24	2.63	26.54
Fe	ppm	102	14746.32	16633.40	5414.47	208.18	22231.29
K	ppm	102	29927.29	31640.00	6671.45	10334.73	40130.00
Li	ppm	102	60.05	64.11	15.77	16.59	83.25
Mg	ppm	102	3016.23	3231.17	1219.76	595.81	5217.54
Mn	ppm	102	353.94	352.65	88.53	108.36	619.82
Na	ppm	102	11681.85	11455.11	2665.78	6362.84	17922.36
Ni	ppm	102	11.08	11.49	6.23	1.69	44.71
Pb	ppm	94	17.36	16.09	7.85	0.08	37.87
Sr	ppm	102	68.31	69.35	12.57	38.35	95.30
Zn	ppm	102	193.87	154.51	116.79	46.58	648.55
Zr	ppm	102	46.21	48.04	12.97	5.63	72.32
Rb	ppm	102	229.12	238.80	61.79	93.47	339.61
V	ppm	99	44.58	50.84	22.44	0.83	84.57
Ti	ppm	102	1622.42	1894.16	719.00	11.03	2652.28
Sand	%	107	54.82	52.44	20.92	14.87	97.39
Silt	%	107	38.39	39.69	17.80	2.06	68.71
Clay	%	107	6.79	7.08	3.41	0.54	16.63
TOC	%	66	2.58	1.14	5.18	0.19	23.04

### Magnetic mineralogy

**Fig. 3:**
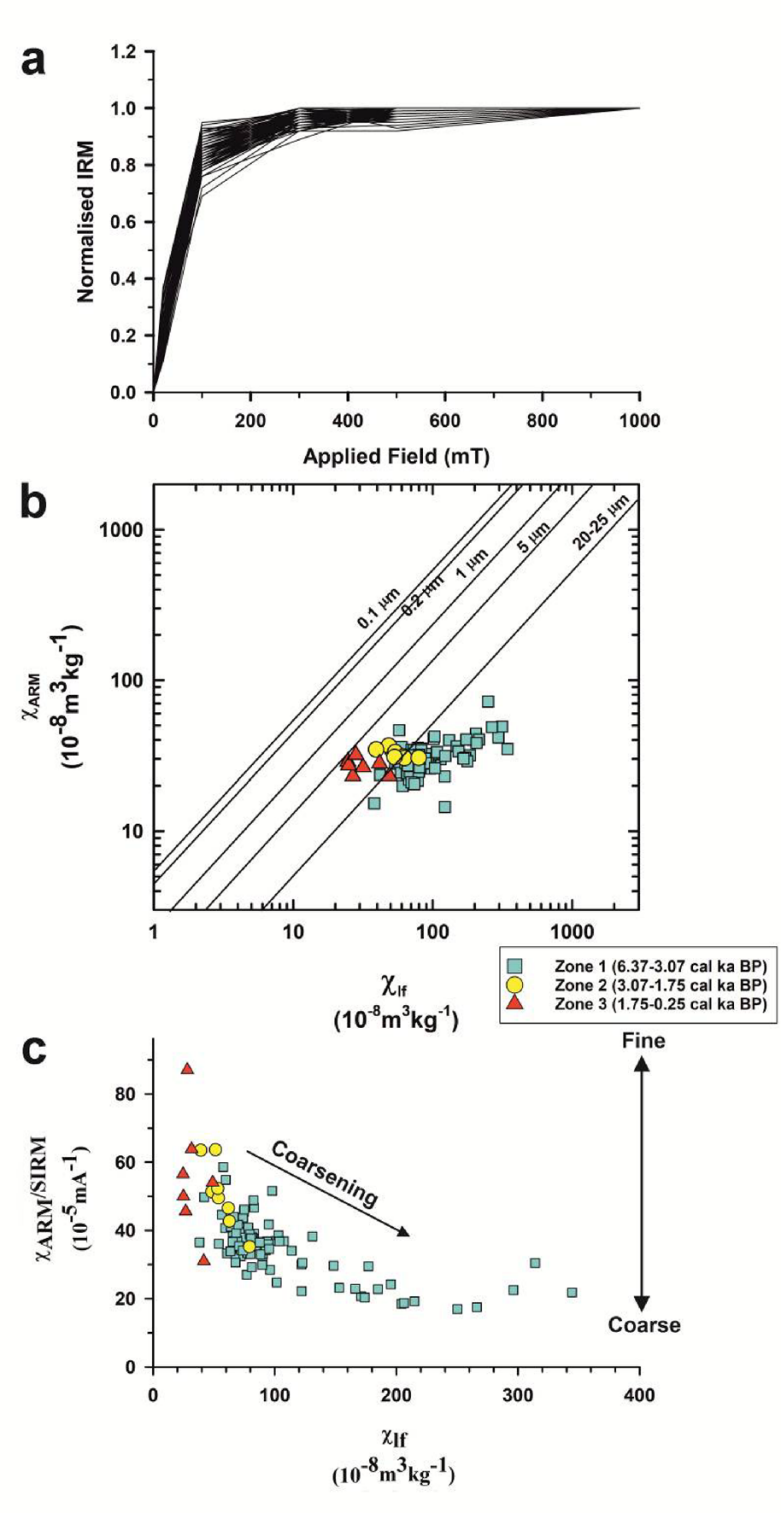
**(a)** Isothermal remanent magnetisation (IRM) acquisition curves for the samples of SL1 core; biplots of **(b)** χ_ARM_ vs. χ_lf_^59^ and **(c)** χ_ARM_/SIRM vs. χ_lf_.

**Fig. 4:**
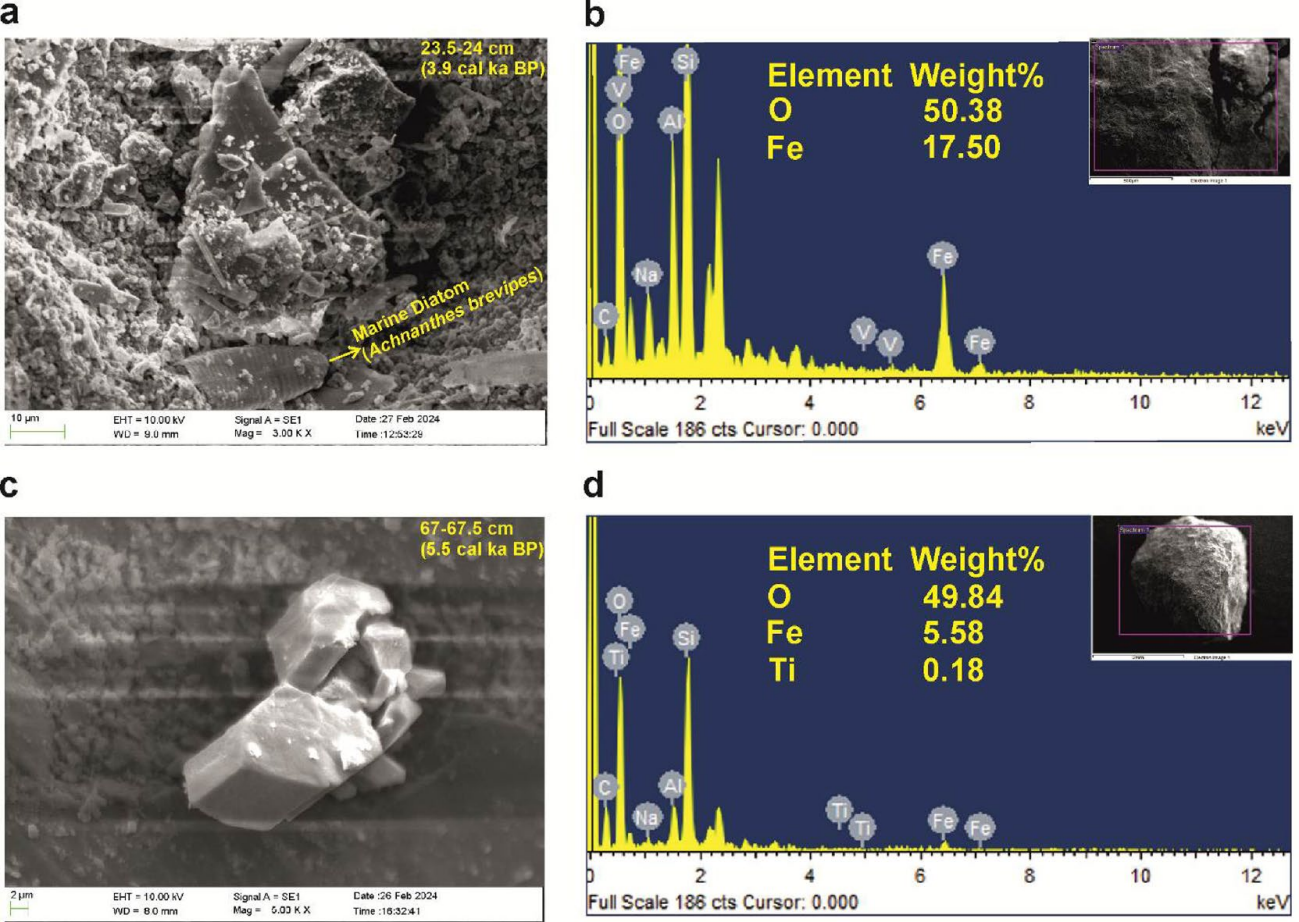
Scanning electron microscopic images of magnetic crystals and their EDS spectra confirming the presence of magnetite and titanomagnetite. Presence of a marine diatom (*Achnanthes brevipes***)** can also be observed in the SEM image of the sample SL1 23.5–24 cm.

The parameters S-ratio and HIRM are used as indicators to identify the magnetic mineralogy. S-ratio (-IRM-_300mT_/SIRM) is a measure of the comparative proportions of the “magnetically soft” minerals (e.g.: magnetite) and the “magnetically hard” minerals (e.g.: hematite) present in the samples^57^. Since magnetite saturates under low fields, typically < 300mT, high S-ratio values indicate the higher proportions of low coercivity minerals in the samples. S-ratio values vary from 0.84 to 1.00 (Table 1), reflecting the prevalence of “magnetically soft” ferrimagnetic minerals. The L-ratio values for the sediment core range between 1.0 and 1.2, reflecting a uniform distribution (Table 1, Supplementary Fig. 4). This suggests that the variations in the HIRM are dominantly controlled by the varying concentrations of hematite rather than any changes in the magnetic coercivities^39,58^. The IRM acquisition curves (Fig. 3a) show that most of the samples saturate at fields below 300mT, signifying the predominance of “low coercivity” minerals such as magnetite and titanomagnetite. This observation is further confirmed by the results from the SEM-EDS analysis of the magnetic extracts, which indicates that the magnetic minerals in Heart Lake predominantly consist of magnetite and titanomagnetite (Fig. 4). The hard fraction of IRM or HIRM is partial to the concentration of “magnetically hard” minerals such as hematite present in the samples. HIRM values vary from 36.6 to 418.9 × 10^− 5^ Am^2^kg^− 1^ with an average (± S.D.) value of 91.6 (± 152.3) × 10^− 5^ Am^2^kg^− 1^ (Table 1). The statistically significant correlation observed between χ_lf_ and HIRM (*r* = 0.79, *p* = 4.5 × 10^− 13^, *n* = 57; Supplementary Table 3) suggests that the “high coercivity” fraction also plays a role in controlling the magnetic signal of Heart Lake sediments.

### Magnetic grain size

The ratios χ_ARM_/SIRM and χ_ARM_/χ_lf_ can be used to decipher the magnetic granulometry. High values of these ratios signify finer magnetic grains, and lower values indicate coarser magnetic grain sizes^38,56^. Both these ratios show a similar trend (Fig. 2) and show a significant correlation (*r* = 0.90, *p* = 2.8 × 10^− 21^, *n* = 57; Supplementary Table 3). χ_ARM_/SIRM ranges from 16.96 to 87.04 × 10^− 5^mA^− 1^ with an average (± S.D.) of 37.02 (± 10.97) × 10^− 5^mA^− 1^, whereas the χ_ARM_/χ_lf_ values vary from a minimum of 0.10 and a maximum of 1.17 with an average (± S.D.) of 0.40 (± 0.19) (Table 1). Both these parameters remain low till ~ 3.07 cal. ka BP and show an increasing trend following this period. The scatter plot of χ_ARM_ vs. χ_lf_^57^ (Fig. 3b) is also indicative of the magnetic particle size. In the plot, all the studied samples are coarse grained having magnetic grain sizes > 5 μm, with most of the samples having a magnetic grain size > 20–25 μm. Coarse-grained magnetic minerals have also been previously reported in the soil samples from the Broknes Peninsula^60^. The biplot of χ_ARM_/SIRM vs. χ_lf_ also indicates the control of coarse magnetic grains in the magnetic susceptibility distribution (Fig. 3c).

### Sources of magnetic minerals

The magnetic minerals present in the sediments are dominantly lithogenous. The catchment geology of Heart Lake consists of metapelites, gneisses and granites of which magnetite is present as an accessory mineral^27^, supporting a terrigenous source for the magnetic minerals. However, the magnetic characteristics of sediments could be affected by the presence of bacterial magnetite^61^, the formation of authigenic Greigite^62^, and the reductive dissolution of magnetic minerals. Bacterial magnetite could be identified by the ratios χ_ARM_/SIRM and χ_ARM_/χ_lf_. χ_ARM_/SIRM values > 200 × 10^− 5^mA^− 1^ and χ_ARM_/χ_lf_ values > 40 × 10^3^ suggests its presence^63^. None of these ratios cross this threshold therefore the presence of bacterial magnetite could be disregarded. Greigite is an iron sulfide that forms under anoxic conditions. SIRM/χ_lf_ values > 30 × 10^3^ Am^− 1^ indicates its presence^62^. For all the studied samples, the SIRM/χ_lf_ values remain well below 30 × 10^3^ Am^− 1,^ suggesting the absence of authigenic Greigite. The reductive dissolution of magnetic minerals typically manifests as a sudden decline in the magnetic grain ratios of χ_ARM_/SIRM and χ_ARM_/χ_lf,_ indicating the coarsening of the magnetic grains. However, such a trend is not observed in the examined core, suggesting no reductive dissolution phases. The ratio 100Ti/Al, a proxy for aeolian influx (Sect. 4.6), shows a very similar trend as to that of HIRM (Supplementary Fig. 5) for the Heart Lake sediments, reflecting the presence of some high coercivity wind-blown grains in SL1 sediment core. The magnetic minerals in Heart Lake may also have a marine origin. The Dalk Glacier, located ~ 2.5 km from Heart Lake (Fig. 1a), lies within its drainage boundary (Supplementary Fig. 6). Glacial sediment inputs from the Dalk Glacier could therefore influence the lake’s magnetic mineral composition. Taken together, the evidence suggests that the magnetic minerals in Heart Lake are most likely derived from a combination of catchment sources, aeolian deposition, and marine inputs.

### Element concentration and weathering

The element concentration in the sediments of core SL1 varies in the order Al > K> Fe > Na> Ca > Mg > Ti > Mn> Ba > Rb> Zn > Ag > Sr > Li> Zr > V> Cr > Pb > Cu > Ni> Co > Cd. Al is found to be most abundant, and its concentration ranges from 1.35% to 5.83% with an average (± S.D.) of 4.02 (± 0.92%).

**Fig. 5:**
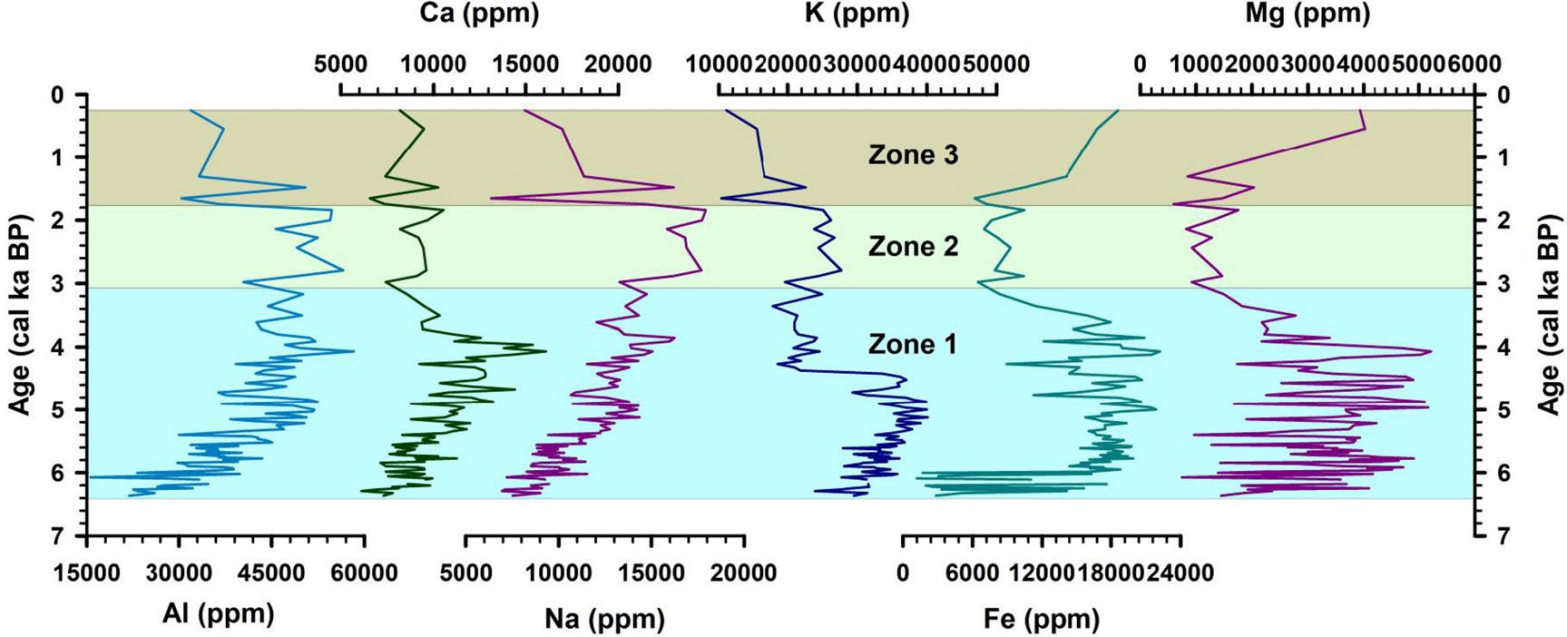
Downcore variations in the major element concentrations for the SL1 sediment core.

**Fig. 6:**
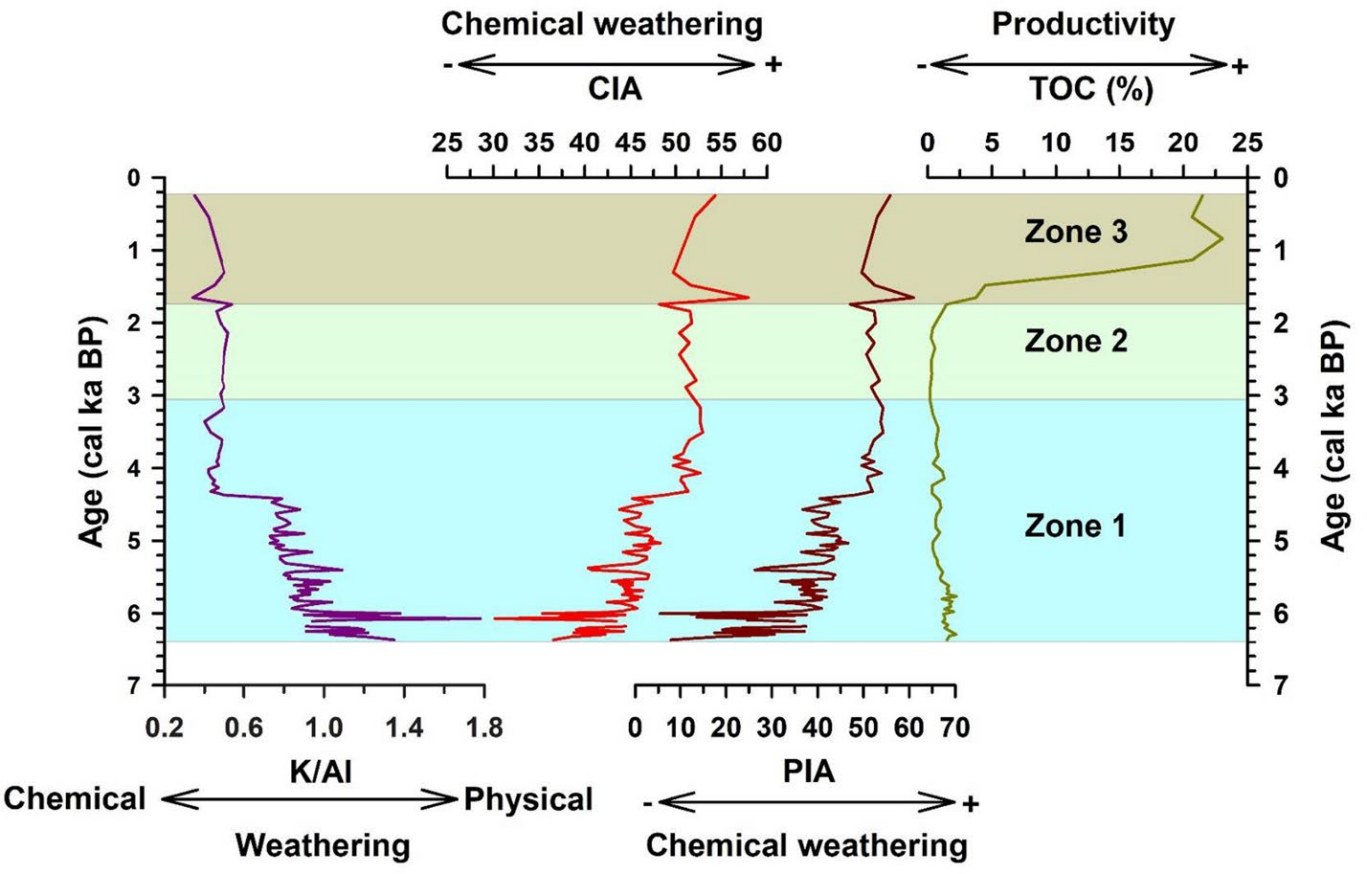
Downcore variations in the geochemical ratios indicating the intensity of physical and chemical weathering and TOC% (a proxy for biogenic productivity).

**Fig. 7:**
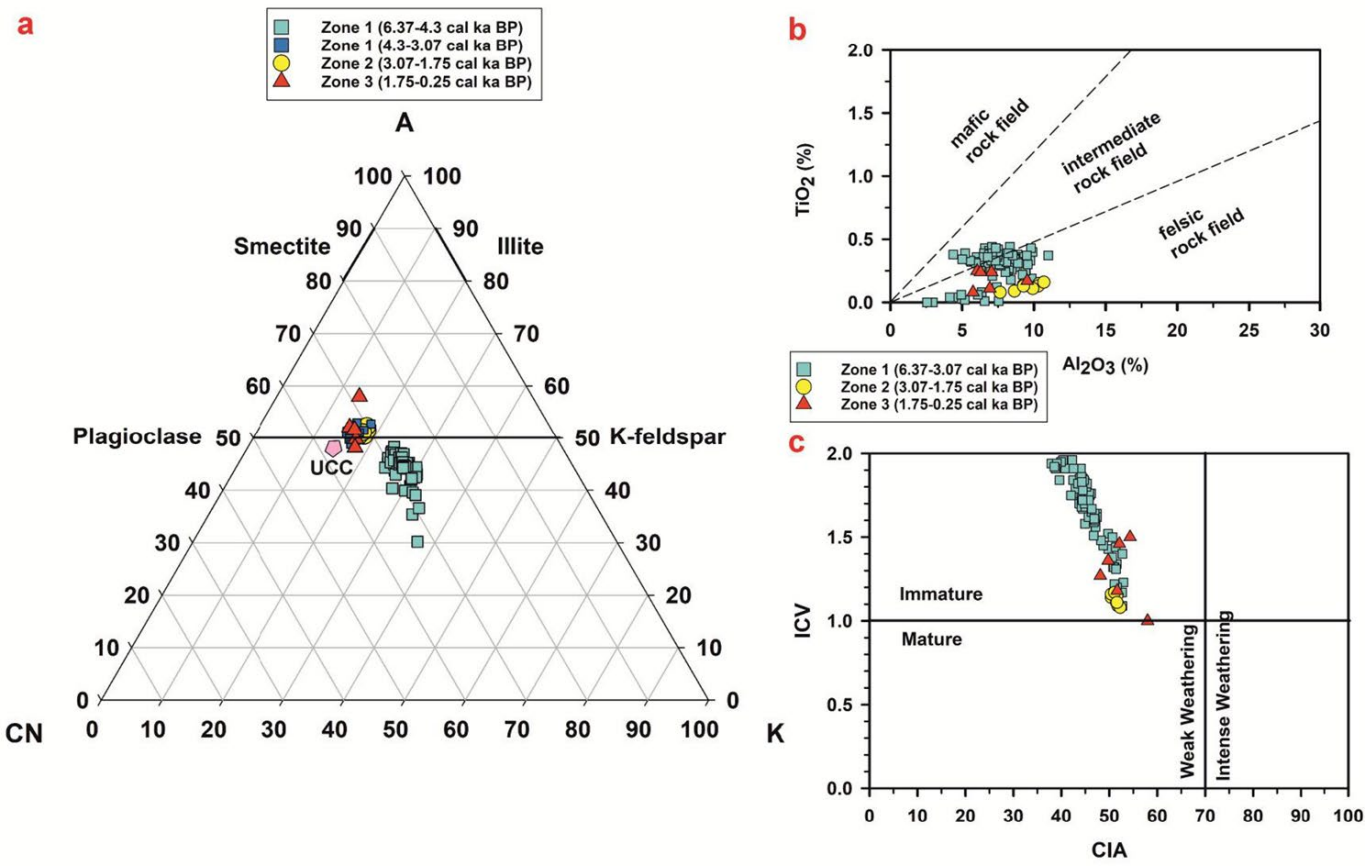
(**a**) Ternary plot of Al_2_O_3_ - (CaO + Na_2_O) - K_2_O (A-CN-K); biplots of (**b**) TiO_2_ vs. Al_2_O_3_ and (**C**) index of compositional variation (ICV) vs. Chemical index of alteration (CIA).

**Fig. 8:**
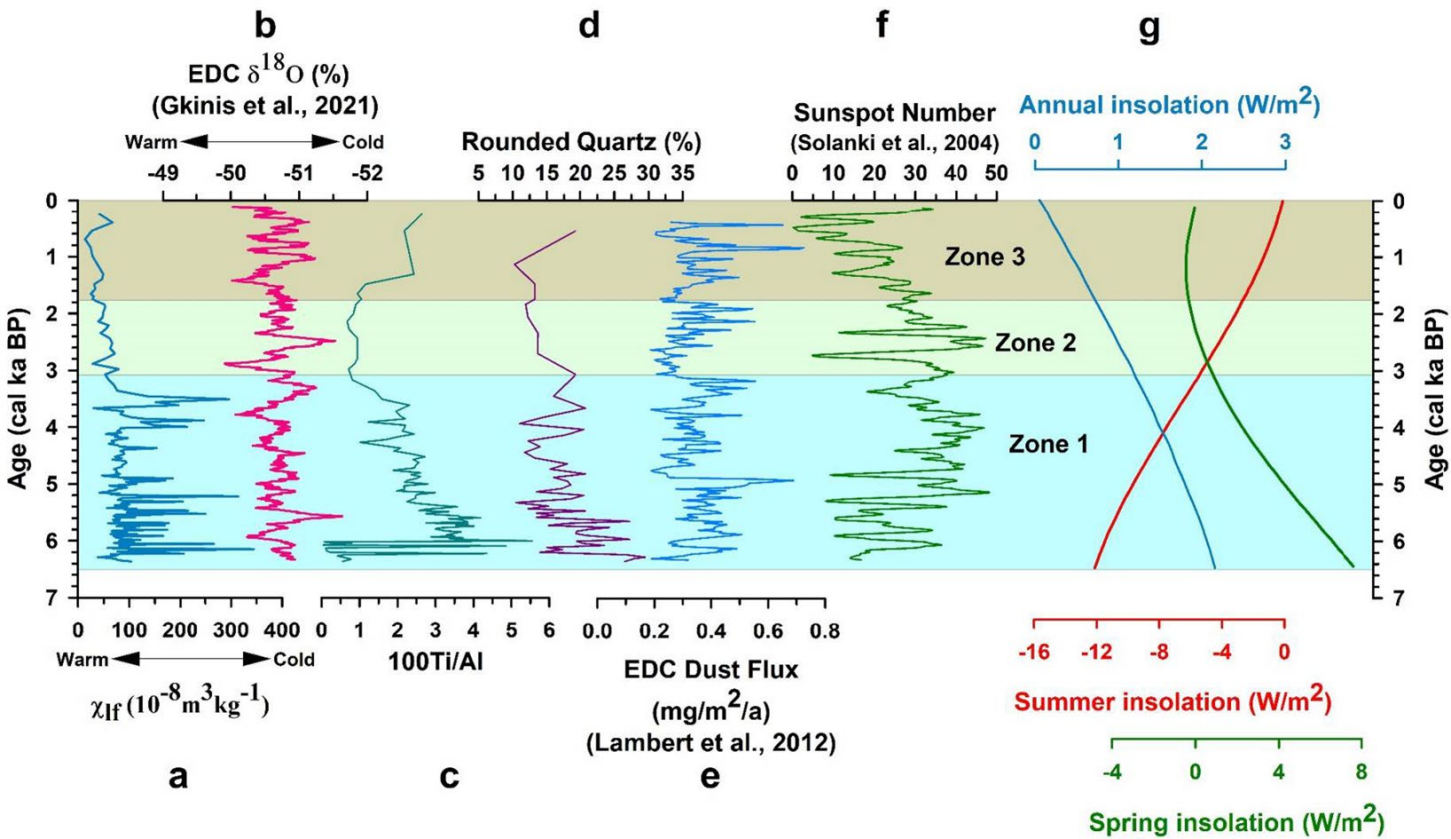
Comparison of **(a)** χ_lf_ data from Heart Lake with **(b)** δ^18^O data of EPICA Dome C (EDC) ice core^73^; **(c)** 100Ti/Al ratio of Heart Lake; **(d)** rounded quartz (%) data from Heart Lake; **(e)** dust flux data from EDC ice core^74^; **(f)** reconstructed sunspot number data^75^, and **(g)** summer, spring, and annual mean insolation at 65°S (modified after ref^76,77^.

The downcore variations in the metal concentrations have been plotted for the significant elements Al, Ca, Na, K, Fe, and Mg (Fig. 5). They show a similar trend up to ~ 4.3 cal ka BP. Following this period, K shows a decreasing trend in Zone 1, while the other metals show an increasing trend, indicating that various factors control the metal concentrations (see Sect. 4.8). The metal concentrations were converted to oxides using a conversion factor to calculate the weathering indices following ref^64–66^. The CIA values for core SL1 range from 3.18 to 57.97, with an average value of 46.16 revealing low to moderate grade of chemical weathering (Fig. 6). PIA values range between 5.35 and 60.94, with an average value of 40.84 suggesting low to moderate alteration taking place in the sediments (Fig. 6). The downcore variations in CIA and PIA show a similar trend, with values remaining low up to ~ 4.3 cal ka BP, following which an increasing trend can be observed. Increasing values of the chemical weathering indices could signify warming conditions in the region. Warm and wet conditions are more conducive to chemical weathering. Increasingly warming conditions could expose the catchment rocks to the atmosphere, leading to the formation of secondary minerals. This change in the weathering trend is also observed in the plot of K/Al, a proxy for weathering. Lower values of this ratio indicate the increasing degree of chemical weathering over physical weathering^67,68^. Values of this ratio remain high up to around 4.3 cal ka BP, following which it shows a declining trend (Fig. 6). In the A-CN-K plot (Fig. 7a), the sediments from Heart Lake can be seen plotting near the join of K-feldspar and plagioclase feldspar, close to the composition of the Upper Continental Crust (UCC). This indicates that the sediments from Heart Lake are primarily unaltered, with a low degree of chemical weathering. The ICV values have been plotted against CIA for the sediments of Heart Lake (Fig. 7b). All the studied samples show ICV values > 1, suggesting that the sediments are compositionally immature and undergoing progressive weathering. The biplot of TiO_2_ vs. Al2O3 indicates the sediment provenance (Fig. 7c). The samples from the studied core plot in the range of intermediate to felsic rock and are consistent with the local geology^27^. The molar ratio 100Ti/Al can be used as a proxy for aeolian input^12,69,70^. Higher values of this ratio indicate a higher influx of aeolian material. 100Ti/Al values range between 0.04 and 5.55, with an average of 2.29. (Fig. 8c). The plot of 100Ti/Al has been compared with the plot of percentage rounded quartz grains from Heart Lake (Fig. 8d). Rounded quartz grains are considered to reflect aeolian transport^71^ and have been previously used as a proxy for aeolian influx in Antarctic Lake sediments^72^. 100Ti/Al data from Heart Lake shows a very similar trend to that of rounded quartz grains, which could reflect the intensity of the aeolian influx in Heart Lake sediments. However, in the end-member analysis (Sect. 4.7), the aeolian component is not reflected. This could be due to the low influx of aeolian material as compared to the sediment deposition by oceanic, glacial and fluvial processes. The TOC% for the sediments of Heart Lake varies from 0.19% to 23.04% with an average (± sd) of 2.58 (± 5.18) %. (Fig. 6). The TOC values remain generally low, suggesting a low degree of productivity in the lake. The highest values of TOC are observed towards the core top, which could be attributed to the presence of cyanobacterial mats in the lake sediments.

### Sediment granulometry and end-member analysis

The particle size analysis of the dataset reveals that the sediments of Heart Lake are predominantly sandy with variable amounts of silt and clay. The mean grain size values for Zone 1 samples vary from 16.9 to 607.5 μm with an average of 90.8 μm. For zone 2 samples, the grain sizes range from 14.4 to 496.7 μm with a mean value of 204 μm, while zone 3 samples have a grain size between 49.3 and 228.3 μm mean grain size of 117.6 μm. In the biplot of skewness vs. sorting (Supplementary Fig. 7), most of the samples are poorly sorted to very poorly sorted and are fine skewed to very fine skewed. The C-M diagram^78^ is useful in identifying the hydrodynamic conditions involved in sediment deposition. The parameter “C” represents the coarse 1 percentile (99th percentile or D99) values of the grain size in microns while “M” represents the median grain size (or D50) in microns. In the C-M diagram of Heart Lake sediments (Supplementary Fig. 8), most of the samples plot along the fields of rolling, rolling and suspension, suspension and rolling and uniform suspension, indicating varying energy conditions of the transporting medium.

**Fig. 9:**
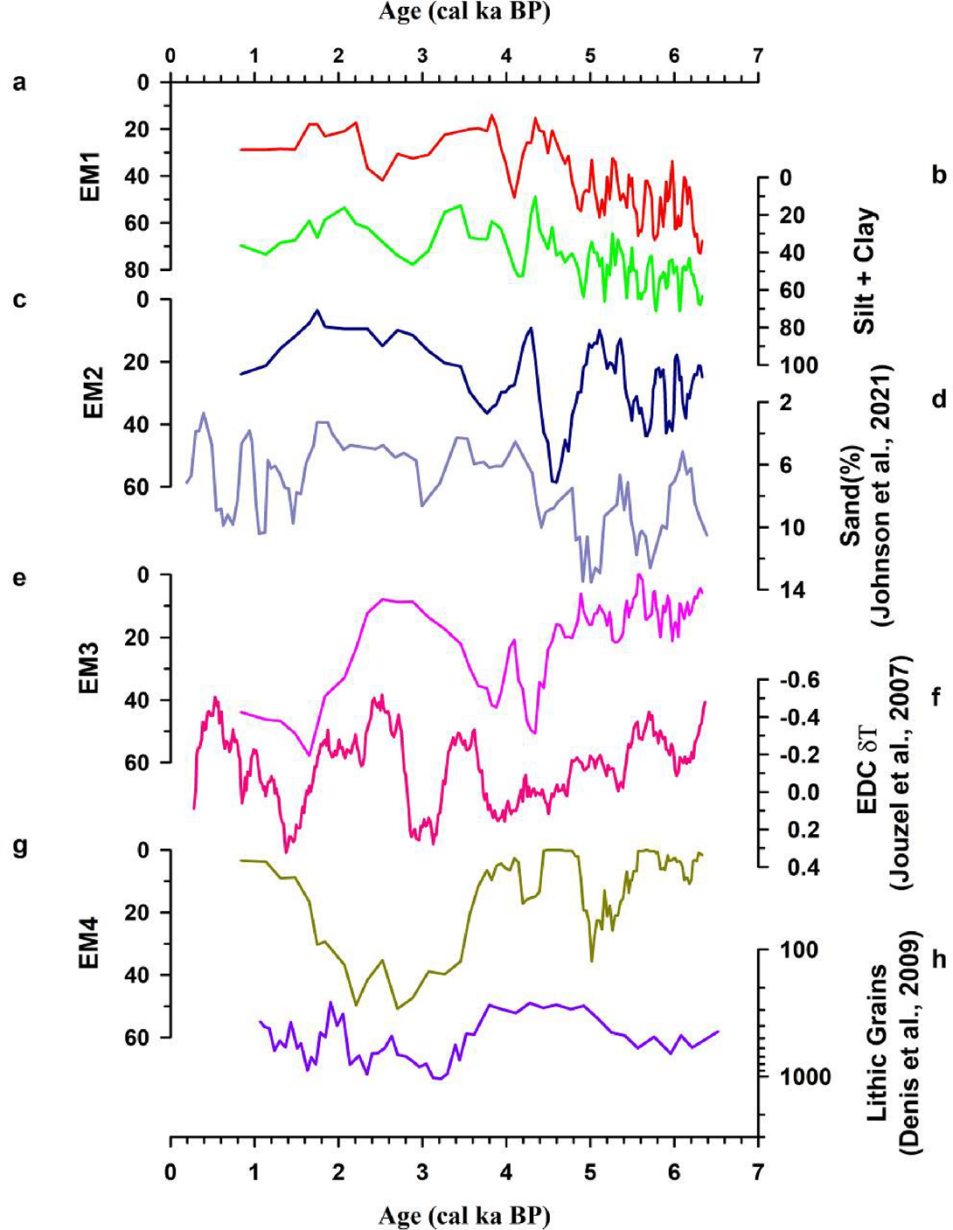
End-member (EM)1 abundance data from Heart Lake **(b)** combined silt and clay percentage from Heart Lake **(c)** EM2 abundance **(d)** Sand percentage data from ref^79^. **(e)** EM3 abundance **(f)** δTemperature data from EPICA Dome C ice core^83^**(g)** EM4 abundance **(h)** lithic grain number data from ref^84^.

The end-member analysis of the particle size data revealed the presence of four end-members EM1, EM2, EM3 and EM4 (Supplementary Fig. 9). EM1 has a mean grain size of 18.7 μm falling in the medium silt fraction. EM1 also shows a very strong statistically significant correlation with silt (*r* = 0.97, *p* = 2.6 × 10^− 68^, *n* = 107; Supplementary Table 5) and clay (*r* = 0.92, *p* = 2.1 × 10^− 43^, *n* = 107; Supplementary Table 5), suggesting that EM1 sediments are principally composed of silt and clay fractions (Fig. 9a and b). In the C-M diagram (Supplementary Fig. 8), EM1 plots along the field of uniform suspension, revealing that EM1 represents the fine-grained component of Heart Lake transported as suspended sediments. EM2 has a mean grain size of 91 μm. EM2 (Fig. 9c) shows a very similar trend to that of the sand percentage record from Adélie Basin, East Antarctica^79^ (Fig. 9d). Ref^79^. considered the sand percentage record to represent the strength of the Antarctic Coastal Current, with an increase in sand percent reflecting increased current strength. The Antarctic Coastal Current, first described by ref^80^., is a wind-driven current that flows westward along the coast of the Antarctic continent^81,82^. In the C-M diagram, EM2 plots along the field of suspension and rolling (Supplementary Fig. 8), suggesting that EM2 represents the current driven sediment resuspension and deposition from graded suspension. The similar trends observed for the plots of EM2, and sand percentage suggest that EM2 is controlled by the Antarctic Coastal Current, with EM2 showing greater abundances during periods of increased current strength. EM3 has a mean grain size of 256.6 μm. EM3 falls in the region of saltation in the C-M diagram (Supplementary Fig. 8), which indicate deposition from saltation population suggesting a possibly a stronger hydraulic condition with respect to uniform and graded suspension driven deposition of finer EMs. The EM3 record (Fig. 9e) is compared with the δT data from EPICA Dome C (EDC) ice core^83^ (Fig. 9f). Major peaks in EM3 coincide with increased Antarctic temperature suggesting that these episodes might represent the meltwater plume derived sedimentation into Heart Lake and EM3 represents the meltwater plume derived saltating grain population. EM4 has a mean grain size of 640.6 μm. In the C-M diagram (Supplementary Fig. 8), EM4 plots in the field of rolling revealing that EM4 is primarily composed of coarse-grained sediments. EM4 represents the coarsest component of Heart Lake sediments and predominantly consists of coarse sand grains, possibly including some Ice-rafted debris (IRD), and represents glaciogenic coarser sediment associated with glacial expansion/retreat. This observation is also supported by the close correlation of EM4 (Fig. 9g) with the lithic grain (> 5 μm) record from Adélie Basin^84^ (Fig. 9h) which represents a record of detrital input controlled by glacier recession/expansion.

### Principal components in the dataset

Principal component analysis of the dataset (environmental magnetic, geochemical and TOC data) was carried out on PAST^45^ (Version 4.03) to understand the control of paleoclimate on the environmental magnetic and elemental data of Heart Lake. The element concentrations were normalized with Al before the analysis to account for any potential dilution or sorting effects. Broken stick model^51,52^ was applied to identify the significant components and four components, PC1, PC2, PC3 and PC4, were found to be significant. These four components account for 69.1% of the variance in the dataset (Supplementary Table 6). PC1 accounts for 32.83% of the total variance. Elements Ag/Al, Ba/Al, Cd/Al, Co/Al, Cr/Al, Cu/Al, Fe/Al, K/Al, Li/Al, Mg/Al, Mn/Al, Zr/Al show significant positive loadings (> 0.6) in PC1. These metals could be associated with the feldspar rocks in the lake catchment^27^. PC2 accounts for 16.68% of the total variance. χ_lf_, χ_fd_, χ_ARM_, SIRM, S_20_ and HIRM show significant positive loadings in PC2. PC2 represents the remanence carrying magnetic minerals in Heart Lake sediments. The component PC3 accounts for 12.23% of the total variance. Ag/Al and Fe/Al show high positive loadings in PC3. A statistically significant correlation is also observed between Ag and Fe (*r* = 0.98, *p* = 9.0 × 10^− 39^, *n* = 57; Supplementary Table 4) indicating their strong association with each other. Since Fe is known to be sensitive to redox conditions^85^, PC3 could represent the redox component. PC4 accounts for 7.37% of the variance. Only TOC show significant positive loadings in PC4 and PC4 represents the sedimentary organic component.

### Paleoenvironmental reconstruction

Previous studies on Heart Lake^5^ revealed that the lake was submarine from 9 to 2.95 cal ka BP, characterised by the presence of marine diatoms. Following this period, lacustrine diatoms were found to be dominant. However, ref^6^. reported the dominance of marine and sea-ice diatoms in the lake sedimentary record till 5 cal ka BP; following this period, freshwater diatoms were found to be dominant. In the present study, based on the diatom data, we were able to identify three distinct zones and reconstruct the paleoenvironmental variations in the lake for the past 6.37 cal ka BP based on environmental magnetic and geochemical data.

#### Zone 1: 6.37 cal Ka BP − 3.07 cal Ka BP – Heart lake as a submarine basin

The period from 6.37 cal ka BP to 3.07 cal ka BP is marked by higher values of magnetic concentration dependent parameters χ_lf_, χ_ARM_ and SIRM (Figs. 2 and 8a). This indicates a higher concentration of magnetic minerals in the sediment samples. The magnetic grain size-dependent parameters, χ_ARM_/SIRM and χ_ARM_/χ_lf_, also show low values during this period (Fig. 2) indicating the dominance of coarse magnetic grains. This observation is also supported by the biplot of χ_ARM_/SIRM vs. χ_lf_ (Fig. 3c), where the samples from zone 1 show coarse magnetic grain sizes. In the Antarctic Lake sediments, generally during periods of colder climate, enriched values of magnetic susceptibility are recorded due to an enhanced supply of coarse-grained ferrimagnetic minerals. Frost weathering dominates during these periods, resulting in the release of strongly magnetic grains, which will be supplied to the lake basin by erosional agents^12,13,15,86^. However, previous studies indicate warm and wet conditions in the Larsemann Hills during this period based on records from three sediment cores, including Heart Lake^5^. The higher values of magnetic susceptibility and coarser magnetic grain sizes observed in our record could be due to the supply of glacial sediments by the nearby Dalk Glacier, which lies at 2.5 km from the lake^5^ (Fig. 1b).

The values of χ_fd_%, which is a semi-quantitative indicator for the pedogenic formation of magnetic minerals of the SP grain size^36,87^, show an oscillating trend with few samples showing higher values in zone 1 (Fig. 2). In the biplot of χ_ARM_ vs. χ_lf_^59^ (Fig. 3b), the samples from zone 1 can be observed to be showing coarser magnetic grain sizes. HIRM shows high values during some intervals in Zone 1 along with 100Ti/Al (Supplementary Fig. 5). This could be due to an enhanced supply of aeolian dust carrying high coercivity magnetic minerals such as hematite^88,89^.

**Fig. 10:**
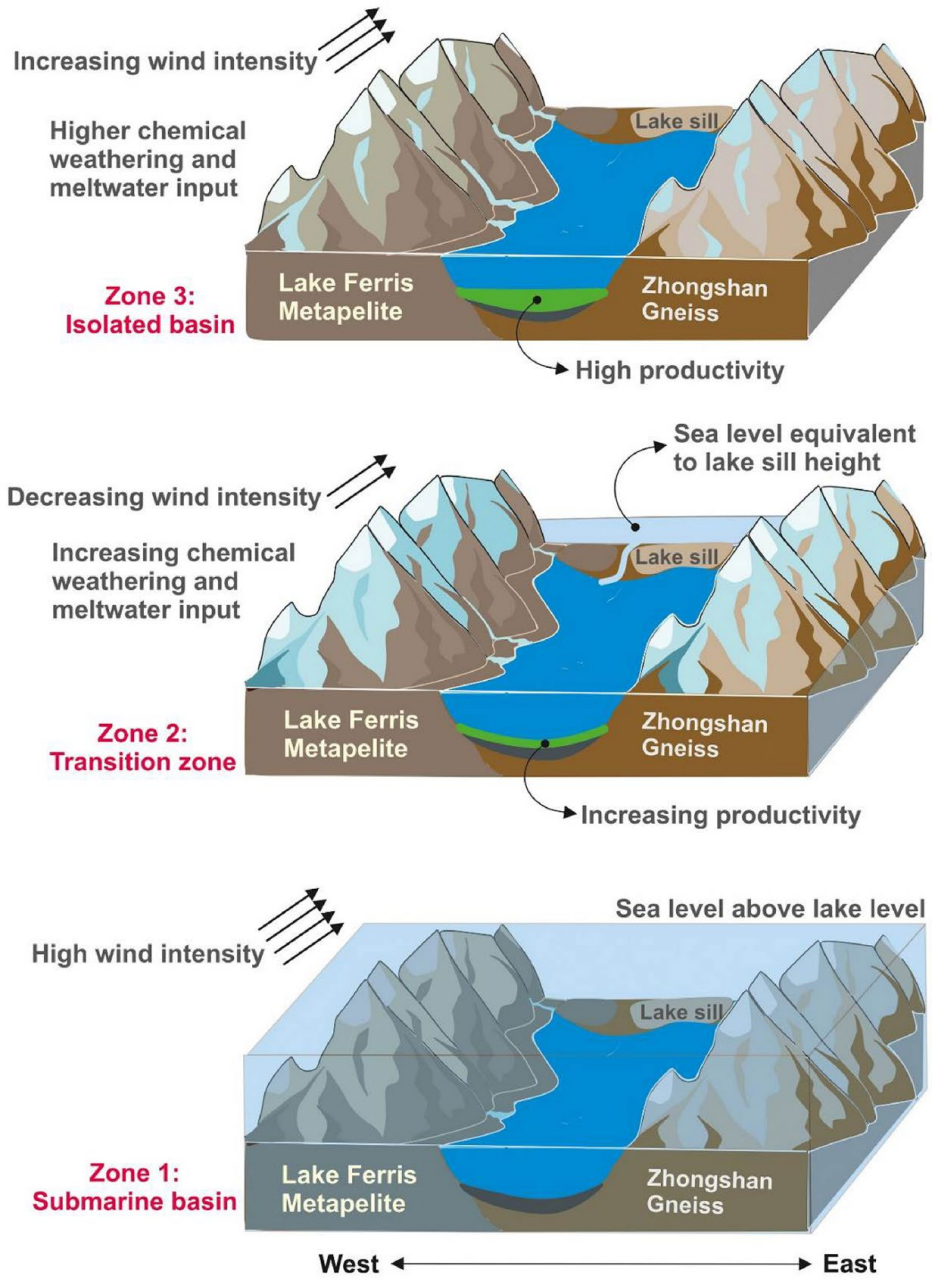
Schematic model representing the paleoenvironmental variations in Heart Lake. The figure was generated using the software CorelDraw2021 (https://www.coreldraw.com/en/).

The chemical weathering indices CIA and PIA show low values in zone 1 (up to ~ 4.3 ka BP) reflecting low degree of chemical weathering in the region during this period (Fig. 6). In the ternary plot of Al_2_O_3_ - (CaO + Na_2_O) - K_2_O (A-CN-K) (Fig. 7a), most samples from zone 1 lie below the feldspar line indicating very low degree of chemical alteration, while few samples show higher degree of alteration. The total organic carbon percentages (TOC) show very low values in Zone 1, suggesting low productivity (Fig. 6). In the biplot of TiO_2_ vs. Al_2_O_3_ (Fig. 7c), the samples from Zone 1 plot between the fields of felsic and intermediate provenance. The felsic province could be from the quartzo-feldspathic Zhongshan gneiss located on the eastern side of the lake. The intermediate provenance could be associated with the progress granite consisting of biotite granite, magnetite and spinel and lake Ferris metapelite consisting of dark garnet, sillimanite, biotite metapelite, with spinel and magnetite variably present located on the western side of the lake^27^. The downcore variations in the metal concentrations of Fe and Mg (Fig. 5) show higher values in zone 1, indicating that a larger proportion of the sediments in zone 1 are sourced from the western catchment of the lake. This points to an enhanced weathering of the western catchment as compared to its eastern counterpart in zone 1 and could be linked to the marine incursion in the lake. In the SL1 sediment core, the results from the diatom analysis indicate that the marine and sea-ice associated diatom *Fragilariopsis curta* dominate the assemblage in zone 1 (Supplementary Table 2). This indicates that the lake was under marine influence during this period (Fig. 10). The marine incursion could have limited the influx of metals from the feldspathic gneissic rocks on the eastern catchment (Supplementary Fig. 11). The ratio 100Ti/Al, a proxy for aeolian input, shows high values in zone 1, indicating a higher influx of aeolian material and higher wind intensity during this period (Fig. 8c). Dust flux record from EDC ice-core^74^ (Fig. 8e) also indicates enhanced wind activity during this period. Higher intensity of katabatic winds could have resulted in the enhanced weathering of the western catchment rocks.

The sediment samples from zone 1 show an average grain size of 90.8 μm, indicating a mixture of finer mud and coarser sand particles. In the C-M diagram (Supplementary Fig. 8), the majority of the samples from zone 1 plot along the fields of uniform suspension and suspension and rolling, suggesting that the sediments are predominantly composed of silt and clay-sized particles with sand-sized particles present in variable amounts. In the downcore plot of end-member abundances (Supplementary Fig. 9), it can be observed that EM1, shows higher values in zone 1. Highest values of EM2 representing the Antarctic coastal current are also observed in zone 1, along with matching peaks in sand percentage data from Adélie Basin (Fig. 9c and d), reflecting stronger currents resulting in an enhanced influx of marine clay, silt and fine sand-sized particles during this interval.

Previous investigation by ref^5^. is also in agreement with our observation that Heart Lake was a submarine between 9 and 3.07 cal ka BP. Located near the coast with a low elevation, the lake has recorded episodes of marine transgression during periods of high RSL. The RSL in the Larsemann Hills was at a regional high stand of about 8–10 m a.p.s.l. between 8.5 and 7.1 cal ka BP^9,11,90^. Between 9.26 and 7.27 cal ka BP, a pronounced rise in RSL was observed, which could be associated with a regression in the degree of isostatic uplift^90^. Following this period, the RSL started to fall, and the region underwent isostatic uplift at an average rate of 3.2 m/ka^7^.

From ~ 4.3 cal ka BP, the chemical weathering indices CIA and PIA show elevated values (Fig. 6), indicating intensified chemical weathering and suggesting the onset of warmer conditions favourable for such processes. This is supported by a concurrent decline in the K/Al ratio, reflecting enhanced leaching of potassium during chemical alteration (Fig. 6). χ_lf_, χ_ARM_ and SIRM also decrease during this period, reinforcing the onset of warming conditions (Fig. 2). The samples from this interval also show a higher degree of alteration in the A-CN-K ternary plot (Fig. 7a). Warming conditions are also reflected in the reconstructed sunspot number data^75^ (Fig. 8f), where an increasing trend is observed from ~ 4.3 ka BP. This warming trend is also observed in the δ^18^O record from the EDC ice-core (Fig. 8b) and several other lacustrine sedimentary records from East Antarctica^15,18,20,91^.

In the C-M diagram (Supplementary Fig. 8), a few samples from zone 1 plot along the fields of rolling and rolling and suspension, indicating higher energy of particle transport. These samples correspond to the period from ~ 4.3 cal ka BP. EM3 shows an increasing trend during this period, reflecting an increased influx of coarse-grained particles into the lake basin by the action of glacial meltwater. Following this interval, EM3 values can be observed to be showing a reducing trend. The major peaks in EM3 coincide with increased Antarctic temperature, except for the interval between ~ 3.7 and 2.7 cal ka BP (Fig. 9e and f). During this period, deposition by the coarsest end-member, EM4 and the current-driven end-members EM1 and EM2 were dominant over EM3. The observed warming trend in the region during this interval may have triggered the breaking of icebergs from the nearby Dalk glacier, leading to the enhanced deposition of glaciogenic particles, possibly including some IRD into the lake basin, thus leading to an increase in EM4 population.

#### Zone 2: 3.07 cal Ka BP – 1.75 cal Ka BP - Transition zone

After 3.07 cal ka BP, the values of χ_lf_, χ_ARM_ and SIRM (Fig. 2) show a sharp decrease in core SL1. The magnetic grain size-dependent parameters χ_ARM_/SIRM and χ_ARM_/χ_lf_ (Fig. 2) can be seen increasing into Zone 2, reflecting finer magnetic grain sizes. In the biplot of χ_ARM_/SIRM vs. χ_lf_ (Fig. 3c) the samples from zone 2 show lower magnetic concentration and finer magnetic grain sizes as compared to those from zone (1) χ_fd_%, which is an indicator of the pedogenic formation of magnetic minerals of the SP grain size^36,37^, registers higher values in zone 2, suggesting increasing intensity of pedogenesis, pointing towards the existence of warmer conditions in the region (Fig. 2). χ_fd_%, values for most samples range between 2 and 8 in zone (2) PC2, representing the remanence carrying minerals (Supplementary Fig. 10) also shows a decreasing trend in Zone 2, indicating an overall reduction in the concentration of magnetic minerals.

The samples from zone 2 plot near the plagioclase feldspar – K-feldspar join, indicating a low grade of alteration in the sediments (Fig. 7a). However, the samples from zone 2 show a higher degree of alteration on average as compared to the samples from zone 1, evident from their position in the A-CN-K diagram. Zone 2 represents a transition to increasingly warm and wet lacustrine conditions in the region (Fig. 10). The chemical weathering indices, CIA and PIA, show higher values in Zone 2, indicating higher intensity of chemical weathering in the catchment during this period (Fig. 6). K/Al shows low values in Zone 2, indicating the dominance of chemical weathering over physical weathering during this interval (Fig. 6). Previous studies also indicate active chemical weathering occurring in the Larsemann Hills^12,92^. The TiO_2_ vs. Al_2_O_3_ biplot reflects a predominantly felsic source for the sediments in zone 2 (Fig. 7c). Low values of 100Ti/Al (Fig. 8c), rounded quartz grains (Fig. 8d) and EDC dust flux data (Fig. 8e) indicate reduced aeolian activity in the region during this period, which could have resulted in the reduction of mineral input from the intermediate to mafic rocks on the western catchment.

The diatom records indicate the presence of an admixture of marine (*F. curta*) and freshwater (*S. inermis*) diatoms beginning from 3.07 cal ka BP (Supplementary Table 2). This mixed assemblage persists till 1.75 cal ka BP, suggesting that this period (3.07 to 1.75 cal ka BP) was a transition zone, i.e., the lake basin water level and the sea level were at the same level, and they interacted over this period recording a mixed assemblage. Hence, Zone 2 represents the onset of the submarine basin being isolated due to the isostatic upliftment, and the signals recorded are a mixed signal (Fig. 10). Ref^5^. reported that the transition from marine to lacustrine conditions occurred at around 2.95 cal ka BP in Heart Lake. The authors reported that a clear transition zone could not be identified in the lake. In the present study, we were able to note the transition zone starting from 3.07 cal ka BP during which time lacustrine diatoms first appeared in the assemblage. The sedimentation rate also shows a sharp decrease from 39.6 cm/ka in Zone 1 to around 7 cm/ka in Zone 2 (Supplementary Fig. 3), indicating a change in the environmental conditions. The RSL records a falling trend during this period reducing to 5 m a.p.s.l. at 2.7 cal ka BP and 4 m at 2.1 cal ka BP^90^. The isostatic uplift continued at a rate of around 1.5 m/ka^7^. A falling RSL in conjunction with the isostatic rebound could have resulted in the lake still coming to the same level as that of the sea level during that period. This could have led to the appearance of mixed diatom taxa in zone 2.

Deglaciation following the LGM has led to rising eustatic sea levels (ESL) along with the rebound of the continental crust. During the early-mid Holocene, the RSL rose along with the rising ESL. After this period, even though the ESL continued to increase between 8 and 5 cal ka BP, RSL shows a falling trend due to glacial isostatic adjustment (GIA)-induced uplift^9^. Even though the ice sheet retreat during deglaciation led to glacial isostatic uplift, the rise in the eustatic sea levels was more prominent, leading to rising RSL across Antarctica^9^. A global sea-level fall of ~ 0.7 m between 4 and 2.5 cal ka BP indicates that the RSL fall during this period in the Larsemann Hills is probably related to the fall in ESL rather than isostatic uplift^90^. An increase in the degree of RSL fall was recorded between 2.8 and 2.5 cal ka BP related to the regional glacial readvances during the mid-late Holocene which could have impeded the crustal uplift rates^90^.

The sediment particle size data shows an increasing trend in the mean grain size in Zone 2, indicating an increase in the energy of the transport medium. From the C-M diagram (Supplementary Fig. 8), it can be observed that most of the samples from Zone 2 plot in the field of rolling, indicating the high energy conditions required for the transport and deposition of coarse sand-sized particles. The plot of EM4 (Fig. 9g), which represents the coarsest particles in Heart Lake, shows an increasing trend, reflecting an enhanced influx of coarse-grained particles in zone 2. The discharge of icebergs from the Dalk glacier could have carried and deposited the coarse-grained sediments in zone 2. EM3 also shows an increasing trend in zone 2 (Fig. 9e), reflecting increasing proportions of coarse-grained particles in the lake during this interval. End-member EM2, reflecting the Antarctic Coastal Current strength, show low values in zone 2, indicating low-intensity currents in the region during this period.

#### Zone 3: 1.75 cal Ka BP – 0.25 cal Ka BP – Heart lake as an isolated basin

The magnetic concentration dependent parameters χ_lf_, χ_ARM_ and SIRM (Fig. 2) show a reducing trend into Zone 3, indicating low concentrations of magnetic minerals. The magnetic grains size-dependent parameters, χ_ARM_/SIRM and χ_ARM_/χ_lf_ (Fig. 2), can be seen increasing into zone 3, indicating finer magnetic grain sizes. The χ_fd_% values remain 0 in Zone 3, suggesting the absence of any SP grains in the samples. The biplots of χ_ARM_ vs. χ_lf_ (Fig. 3b) and χ_ARM_/SIRM vs. χ_lf_ (Fig. 3c) also indicate the lowest concentrations of magnetic minerals in Zone 3, along with finer magnetic grain sizes. From the plots of CIA and PIA (Fig. 6), we can infer that the degree of chemical weathering is highest in zone 3. TOC shows a sharp increase in Zone 3 (Fig. 6), along with PC4 (Supplementary Fig. 10), which could be due to the presence of cyanobacterial mats in the core-top, signalling conditions favourable for higher organic productivity.

The diatom data indicates the dominance of freshwater diatoms (*Stauroforma inermis*) and the absence of marine diatoms in Zone 3, reflecting the isolation of the basin and transition to a freshwater environment (Supplementary Table 2). The sedimentation rate is also the lowest in zone 3 with an average value of 5.56 cm/ka. The fall in RSL could have resulted in the isolation of the lake and a transition in the lake environment from marine to lacustrine by ~ 1.75 cal ka BP. The transition phase lasted till around 1.75 cal ka BP, following which the lake became isolated and free from marine influence (Fig. 10), which is marked by the absence of marine and sea-ice-associated diatoms. The lowest concentrations of the magnetic minerals are observed in Zone 3, along with reducing metal concentrations, coincident with the lake isolation and a progressively warming climate. Warming conditions in the region during the mid-late Holocene could have led to enhanced melting of ice caps and subsequent uplift of the crust. The warming conditions reflected in our proxy records agree with the EDC δ^18^O record (Fig. 8b) which also indicates a warming climate during this period. High values of summer, spring and annual insolation values at 65°S can also be observed during this interval (Fig. 8g).

The C-M diagram (Supplementary Fig. 8) of zone 3 samples indicates that they are carried in rolling and suspension and suspension and rolling, reflecting a mixed assemblage of coarse and fine-grained particles. End-member abundances also show low values in Zone 3, reflecting reduced sediment influx into the lake, probably due to the lake isolation. However, EM4 shows a slightly increasing trend, which could be the result of local sediment deposition by the melting snowbanks along the lake catchment^93^.

An increasing trend can be observed in the values of χ_lf_, χ_ARM_ and SIRM, during the late Holocene (Fig. 2). 100Ti/Al and rounded quartz data also show an increasing trend during this period, indicating increasing aeolian influx. These observations could represent a late Holocene cooling event, coeval with the Little Ice Age (LIA). However, low sample resolution during this interval restricts the interpretation of the climate signal. But previous studies indicate increasing dust flux in Antarctica during the Late Holocene, which was attributed to colder temperatures and enhanced strength of katabatic winds during this period^94,95^.

## Conclusions

This study presents a multi-proxy reconstruction of mid- to late-Holocene environmental change from Heart Lake, a coastal basin in the Larsemann Hills, East Antarctica. Diatom assemblages, magnetic parameters, geochemical indices, and sedimentological data reveal a distinct transition from marine to lacustrine conditions over the past ~ 6.37 ka.

Three main environmental phases were identified:

- Marine phase (~ 6.37–3.07 cal ka BP): The lake existed as a submarine basin, with high magnetic concentrations, coarse grains, and low chemical weathering indices indicating comparatively mild climate and stable conditions. Sediment composition was influenced by the Antarctic Coastal Current. From ~ 4.3 ka, a warming trend is observed in the lake.
- Transitional phase (~ 3.07–1.75 cal ka BP): The onset of lacustrine diatoms marks a shift toward a mixed environment. Declining magnetic concentrations, finer magnetic grain sizes, and rising weathering indices are observed in this zone. Increased coarse sediment input suggests intensified iceberg calving from the nearby Dalk Glacier, likely linked to a mid-Holocene warming.
- Freshwater phase (post-1.75 cal ka BP): The lake became fully isolated. The absence of marine indicators, reduced sedimentation rates, and enhanced chemical weathering reflect warm and wet conditions. A late Holocene cooling event, possibly associated with the LIA, can be observed in the record. However, low sample resolution during this interval restricts the interpretation of this climate signal.

Together, these findings underscore the interplay between regional climate variability, glacial dynamics, and marine-lacustrine transitions in shaping Antarctic coastal lake environments.

## Supplementary Information

Below is the link to the electronic supplementary material.

Supplementary Material 1

Supplementary Material 2

Supplementary Material 3

Supplementary Material 4

Supplementary Material 5

## Data Availability

The authors declare that the data supporting the findings of this study are available within the paper, its supplementary information files and extended data files.
