# Supplementary material for: Environmental evolution of a coastal lake in the Larsemann Hills, East Antarctica during the Holocene: a multi-proxy perspective
Source: Sci Rep. 2026 Feb 15;16:9139. doi: 10.1038/s41598-026-39218-8 (PMC12996284; doi:10.1038/s41598-026-39218-8)
Supplement: Supplementary file 1 — Supplementary Material 1 [file 41598_2026_39218_MOESM1_ESM.docx]

**Environmental Evolution of a Coastal Lake in the Larsemann Hills, East Antarctica during the Holocene: A Multi-Proxy Perspective**

G.S. Joju^1^, Anish Kumar Warrier^1,*^, Mahesh, B.S.^2^, Avirajsinh Jadav^2^, Cheryl A Noronha-D'Mello^2^, Masud Kawsar^3,4^, Manoj M.C.^4^, Yamuna Sali A.S.^1^, Gokul Valsan^1^, Krishnaprasad A.A.^5^, Shardool Kokare^5^, K. Balakrishna^6^ and Rahul Mohan^2^

^1^Centre for Climate Studies, Manipal Institute of Technology, Manipal Academy of Higher Education, Manipal, 576104, Karnataka, India

^2^Antarctic Science Division, National Centre for Polar and Ocean Research, Headland Sada, Vasco-da-Gama, 403804, Goa, India

^3^Indian Institute of Tropical Meteorology, Ministry of Earth Sciences, Pune, 411008, India

^4^Birbal Sahni Institute of Palaeosciences, 53 University Road, Lucknow, Uttar Pradesh, 226007, India

^5^Department of Sciences, Manipal Institute of Technology, Manipal Academy of Higher Education, Manipal 576104, Karnataka, India

^6^Centre for Smart Coastal Sustainability, Manipal Institute of Technology, Manipal Academy of Higher Education, Manipal, 576104, Karnataka, India

**Supplementary Note 1**

In the transitional zone of the core, the significant appearance of *Amphora veneta* marks the shift from marine to freshwater conditions. A few valves of *Luticola muticopsis* were also observed in association with *A. veneta*. This interval is observed by a decline in the dominance of marine species and a notable increase in freshwater taxa, indicating the onset of environmental transition. Eventually, freshwater species became dominant, while marine species and *A. veneta* began to disappear, which has been interpreted as marking the end of the transitional phase.

**Supplementary Note 2**

We have modelled the grain size compositional data, with 4 End Members (EM) in order to simplify the process interpretation and avoid EM repetition. Supplementary Figure 2 demonstrates the model fit statistics and a justification for choice of a 4 EM model. The end-member correlation (a measure of redundancy, where lower is better) is 0.06 in the 4 EM model, indicating good independence among end members. Furthermore, the inflection point in both the R² and angular deviation curves occurs at 4 EMs, beyond which additional end members do not significantly improve model performance or reduce the number of outliers. Therefore, the 4 EM model represents an optimal trade-off between model accuracy and complexity, providing a robust and interpretable solution for end-member unmixing.

Supplementary Table 1: Details of AMS ^14^C dates obtained for Stepped Lake sediment core. The ^14^C ages were calibrated and converted to calendar ages by using code Bacon^1^ (version 3.0.0) running on ‘R’^2^ (version 4.2.0), using SHCal20^3^ and Marine20^4^ calibration curves.

| Lab ID | Depth (cm) | δ^13^C (‰) | ^14^C age (yr BP) | Reservoir corrected age (yr BP) | Mean cal. age (yr BP) | δ^14^C error | Calibration curve used |
| --- | --- | --- | --- | --- | --- | --- | --- |
| AA105017 | 0.0-1.0 | -9.9 | 198 | - | 248 | 40 | SHCal20 |
| AA109246 | 4.0-5.0 | -10.7 | 1421 | - | 1308 | 23 | SHCal20 |
| AA109247 | 10.5-11.0 | -13.6 | 2024 | 724 | (outlier) | 30 | Marine20 |
| AA106637 | 20.5-21.0 | -18.6 | 5200 | 3900 | 3666 | 30 | Marine20 |
| AA106638 | 41.0-41.5 | -17.6 | 6032 | 4732 | 4761 | 35 | Marine20 |
| AA105018 | 62.0-62.5 | -18.2 | 6686 | 5386 | 5425 | 33 | Marine20 |
| AA106640 | 101.0-101.5 | -18.5 | 7063 | 5763 | 6128 | 34 | Marine20 |
| AA105019 | 112-112.5 | -18.2 | 7186 | 5886 | 6366 | 33 | Marine20 |

Supplementary Table 2: Distribution of dominat diatom species for SL1 sediment core

| Depth (cm) | Age (cal yr BP) | Dominant diatom species |
| --- | --- | --- |
| 1 | 248 | Not enough samples |
| 2 | 544 | Not enough samples |
| 3 | 840 | *S. inermis* |
| 4 | 1136 | Not enough samples |
| 5 | 1308 | *S. inermis* |
| 6 | 1481 | *S. inermis* |
| 7 | 1653 | *S. inermis* |
| 8 | 1746 | *S. inermis* |
| 9 | 1839 | *S. inermis* with rare *F. curta* |
| 10 | 1932 | *S. inermis* with *F. curta* |
| 11 | 2070 | *S. inermis* with *F. curta* |
| 12 | 2207 | *S. inermis* with *F. curta* |
| 13 | 2345 | *F. curta* slightly more than *S. inermis* |
| 14 | 2524 | *F. curta* with few *S. inermis* |
| 15 | 2703 | *F. curta* with rare *S. inermis* |
| 16 | 2883 | *F. curta* with rare *S. inermis* |
| 17 | 3073 | *F. curta* with rare *S. inermis* |
| 18 | 3264 | *F. curta* |
| 19 | 3455 | *F. curta* |
| 20 | 3560 | *F. curta* |
| 21 | 3666 | *F. curta* |
| 22 | 3771 | *F. curta* |
| 23 | 3826 | *F. curta* |
| 24 | 3881 | *F. curta* |
| 25 | 3937 | *F. curta* |
| 30 | 4195 | *F. curta* |
| 40 | 4699 | *F. curta* |
| 50 | 5048 | *F. curta* |
| 60 | 5359 | *F. curta* |
| 70 | 5569 | *F. curta* |
| 80 | 5749 | *F. curta* |
| 90 | 5923 | *F. curta* |
| 100 | 6099 | *F. curta* |
| 110 | 6311 | *F. curta* |

Supplementary Table 3: Pearson’s correlation table for environmental magnetic and sedimentary organic matter data. Significant correlations (p<0.05) are highlighted in bold.

|  | **χ_lf_** | **χ_fd_** | **χ_fd_%** | **χ_ARM_** | **IRM_1000mT_** | **χ_ARM_/SIRM** | **χ_ARM_/χ_lf_** | **S_20_** | **S-ratio** | **HIRM** | **TOC (%)** |
| --- | --- | --- | --- | --- | --- | --- | --- | --- | --- | --- | --- |
| **χ_lf_** | **1.00** |  |  |  |  |  |  |  |  |  |  |
| **χ_fd_** | **0.60** | **1.00** |  |  |  |  |  |  |  |  |  |
| **χ_fd_%** | 0.06 | **0.70** | **1.00** |  |  |  |  |  |  |  |  |
| **χ_ARM_** | **0.61** | **0.65** | 0.21 | **1.00** |  |  |  |  |  |  |  |
| **IRM_1000mT_** | **0.79** | **0.77** | 0.21 | **0.83** | **1.00** |  |  |  |  |  |  |
| **χ_ARM_/SIRM** | **-0.65** | **-0.52** | -0.20 | -0.22 | **-0.62** | **1.00** |  |  |  |  |  |
| **χ_ARM_/χ_lf_** | **-0.64** | **-0.45** | -0.20 | -0.09 | **-0.46** | **0.90** | **1.00** |  |  |  |  |
| **S_20_** | **0.61** | **0.47** | 0.12 | **0.30** | **0.48** | **-0.47** | **-0.35** | **1.00** |  |  |  |
| **S-ratio** | 0.12 | 0.18 | 0.07 | 0.15 | 0.17 | 0.10 | 0.21 | **0.27** | **1.00** |  |  |
| **HIRM** | **0.79** | **0.77** | 0.21 | **0.83** | **1.00** | **-0.62** | **-0.45** | **0.48** | 0.20 | **1.00** |  |
| **TOC (%)** | -0.19 | -0.23 | **-0.30** | -0.23 | -0.17 | 0.19 | **0.31** | -0.03 | 0.14 | -0.17 | **1.00** |
|  |  |  |  |  |  |  |  |  |  |  |  |

Supplementary Table 4: Pearson’s correlation table for elemental concentrations and sedimentary organic matter data. Significant correlations (p<0.05) are highlighted in bold.

|  | **Ag** | | **Al** | | **B** | | **Ba** | | **Ca** | | **Cd** | | **Co** | **Cr** | | **Cu** | | **Fe** | **K** | | **Li** | | **Mg** | | **Mn** | **Na** | | **Ni** | | **Pb** | **Sr** | | **Tl** | | **Zn** | | **Zr** | | **Rb** | | | **V** | **Ti** | **TOC (%)** |
| --- | --- | --- | --- | --- | --- | --- | --- | --- | --- | --- | --- | --- | --- | --- | --- | --- | --- | --- | --- | --- | --- | --- | --- | --- | --- | --- | --- | --- | --- | --- | --- | --- | --- | --- | --- | --- | --- | --- | --- | --- | --- | --- | --- | --- |
| **Ag** | | **1.00** | |  | |  | |  | |  | |  |  | |  | |  |  | |  | |  | |  |  | |  | |  |  | |  | |  | |  | |  | |  |  | |  |  |
| **Al** | | **0.31** | | **1.00** | |  | |  | |  | |  |  | |  | |  |  | |  | |  | |  |  | |  | |  |  | |  | |  | |  | |  | |  |  | |  |  |
| **B** | | **0.58** | | 0.25 | | **1.00** | |  | |  | |  |  | |  | |  |  | |  | |  | |  |  | |  | |  |  | |  | |  | |  | |  | |  |  | |  |  |
| **Ba** | | **0.61** | | **0.85** | | **0.39** | | **1.00** | |  | |  |  | |  | |  |  | |  | |  | |  |  | |  | |  |  | |  | |  | |  | |  | |  |  | |  |  |
| **Ca** | | **0.36** | | **0.70** | | **0.48** | | **0.72** | | **1.00** | |  |  | |  | |  |  | |  | |  | |  |  | |  | |  |  | |  | |  | |  | |  | |  |  | |  |  |
| **Cd** | | **0.46** | | **-0.33** | | **0.38** | | 0.01 | | 0.07 | | **1.00** |  | |  | |  |  | |  | |  | |  |  | |  | |  |  | |  | |  | |  | |  | |  |  | |  |  |
| **Co** | | **0.85** | | 0.01 | | **0.57** | | **0.32** | | 0.24 | | **0.59** | **1.00** | |  | |  |  | |  | |  | |  |  | |  | |  |  | |  | |  | |  | |  | |  |  | |  |  |
| **Cr** | | **0.97** | | 0.16 | | **0.58** | | **0.51** | | **0.28** | | **0.59** | **0.90** | | **1.00** | |  |  | |  | |  | |  |  | |  | |  |  | |  | |  | |  | |  | |  |  | |  |  |
| **Cu** | | **0.36** | | **-0.36** | | **0.27** | | -0.06 | | -0.03 | | **0.65** | **0.72** | | **0.51** | | **1.00** |  | |  | |  | |  |  | |  | |  |  | |  | |  | |  | |  | |  |  | |  |  |
| **Fe** | | **0.98** | | **0.37** | | **0.61** | | **0.64** | | **0.40** | | **0.40** | **0.82** | | **0.95** | | **0.31** | **1.00** | |  | |  | |  |  | |  | |  |  | |  | |  | |  | |  | |  |  | |  |  |
| **K** | | **0.42** | | -0.02 | | 0.07 | | 0.26 | | 0.09 | | **0.34** | **0.36** | | **0.47** | | **0.37** | **0.36** | | **1.00** | |  | |  |  | |  | |  |  | |  | |  | |  | |  | |  |  | |  |  |
| **Li** | | **0.39** | | -0.23 | | 0.18 | | 0.15 | | -0.02 | | **0.59** | **0.39** | | **0.50** | | **0.56** | **0.30** | | **0.79** | | **1.00** | |  |  | |  | |  |  | |  | |  | |  | |  | |  |  | |  |  |
| **Mg** | | **0.69** | | **0.33** | | **0.52** | | **0.66** | | **0.56** | | **0.48** | **0.68** | | **0.70** | | **0.55** | **0.66** | | **0.42** | | **0.60** | | **1.00** |  | |  | |  |  | |  | |  | |  | |  | |  |  | |  |  |
| **Mn** | | **0.69** | | **0.37** | | **0.60** | | **0.49** | | **0.59** | | **0.34** | **0.63** | | **0.68** | | **0.31** | **0.71** | | **0.51** | | **0.37** | | **0.60** | **1.00** | |  | |  |  | |  | |  | |  | |  | |  |  | |  |  |
| **Na** | | -0.04 | | **0.89** | | 0.07 | | **0.59** | | **0.52** | | **-0.56** | **-0.32** | | -0.19 | | **-0.62** | 0.05 | | **-0.27** | | **-0.51** | | -0.05 | 0.08 | | **1.00** | |  |  | |  | |  | |  | |  | |  |  | |  |  |
| **Ni** | | 0.23 | | **-0.34** | | 0.22 | | -0.21 | | -0.14 | | **0.32** | **0.67** | | **0.34** | | **0.81** | 0.23 | | -0.02 | | 0.10 | | **0.29** | 0.10 | | **-0.47** | | **1.00** |  | |  | |  | |  | |  | |  |  | |  |  |
| **Pb** | | -0.01 | | **0.73** | | 0.01 | | **0.42** | | **0.26** | | **-0.59** | **-0.29** | | -0.16 | | **-0.67** | 0.08 | | **-0.41** | | **-0.69** | | **-0.30** | -0.10 | | **0.88** | | **-0.39** | **1.00** | |  | |  | |  | |  | |  |  | |  |  |
| **Sr** | | **0.41** | | **0.89** | | **0.41** | | **0.89** | | **0.83** | | -0.16 | 0.25 | | **0.30** | | -0.09 | **0.48** | | -0.03 | | -0.14 | | **0.56** | **0.43** | | **0.70** | | -0.06 | **0.51** | | **1.00** | |  | |  | |  | |  |  | |  |  |
| **Tl** | | 0.09 | | **-0.67** | | 0.01 | | **-0.35** | | **-0.30** | | **0.66** | 0.22 | | 0.24 | | **0.50** | -0.01 | | **0.53** | | **0.78** | | 0.19 | 0.09 | | **-0.80** | | 0.11 | **-0.83** | | **-0.58** | | **1.00** | |  | |  | |  |  | |  |  |
| **Zn** | | 0.01 | | **0.72** | | 0.00 | | **0.56** | | **0.58** | | **-0.33** | -0.21 | | -0.10 | | **-0.39** | 0.06 | | -0.19 | | **-0.34** | | 0.08 | 0.13 | | **0.70** | | **-0.40** | **0.58** | | **0.66** | | **-0.56** | | **1.00** | |  | |  |  | |  |  |
| **Zr** | | **0.61** | | 0.09 | | **0.46** | | **0.27** | | 0.26 | | **0.43** | **0.64** | | **0.64** | | **0.43** | **0.53** | | **0.43** | | **0.38** | | **0.47** | **0.51** | | -0.14 | | **0.28** | -0.17 | | 0.13 | | 0.22 | | -0.08 | | **1.00** | |  |  | |  |  |
| **Rb** | | **0.29** | | **0.61** | | 0.25 | | **0.71** | | **0.60** | | -0.06 | 0.09 | | 0.21 | | -0.06 | **0.28** | | 0.12 | | 0.17 | | **0.59** | 0.25 | | **0.41** | | -0.22 | 0.19 | | **0.67** | | -0.23 | | **0.51** | | **0.38** | | **1.00** |  | |  |  |
| **V** | | **0.73** | | 0.13 | | **0.37** | | **0.39** | | **0.27** | | **0.39** | **0.70** | | **0.73** | | **0.36** | **0.66** | | **0.42** | | **0.35** | | **0.48** | **0.48** | | -0.15 | | 0.24 | -0.08 | | 0.21 | | 0.17 | | -0.04 | | **0.81** | | **0.39** | **1.00** | |  |  |
| **Ti** | | **0.74** | | 0.23 | | **0.40** | | **0.45** | | **0.34** | | **0.28** | **0.67** | | **0.71** | | **0.28** | **0.67** | | **0.40** | | **0.29** | | **0.47** | **0.54** | | -0.04 | | 0.18 | 0.00 | | **0.30** | | 0.07 | | 0.03 | | **0.82** | | **0.45** | **0.98** | | **1.00** |  |
| **TOC (%)** | | -0.04 | | -0.17 | | 0.07 | | -0.25 | | -0.17 | | -0.15 | **0.32** | | -0.01 | | **0.40** | 0.02 | | **-0.43** | | **-0.37** | | -0.02 | -0.18 | | -0.14 | | **0.83** | 0.00 | | 0.04 | | **-0.32** | | -0.21 | | -0.05 | | -0.23 | -0.05 | | -0.05 | **1.00** |

Supplementary Table 5: Pearson’s correlation table for end-member abundance and sediment particle size data. Significant correlations (p<0.05) are highlighted in bold.

|  | **Sand** | **Silt** | **Clay** | **EM 1** | **EM 2** | **EM 3** | **EM 4** |
| --- | --- | --- | --- | --- | --- | --- | --- |
| **Sand** | **1.00** |  |  |  |  |  |  |
| **Silt** | **-1.00** | **1.00** |  |  |  |  |  |
| **Clay** | **-0.93** | **0.90** | **1.00** |  |  |  |  |
| **EM 1** | **-0.98** | **0.97** | **0.92** | **1.00** |  |  |  |
| **EM 2** | 0.06 | -0.04 | -0.18 | **-0.25** | **1.00** |  |  |
| **EM 3** | **0.69** | **-0.70** | **-0.59** | **-0.67** | -0.18 | **1.00** |  |
| **EM 4** | **0.64** | **-0.65** | **-0.55** | **-0.52** | **-0.39** | 0.11 | **1.00** |

Supplementary Table 6: Loading data along with variance for the significant principal components. Significant loadings (>0.6) have been highlighted in bold.

|  | **PC 1** | **PC 2** | **PC 3** | **PC 4** |
| --- | --- | --- | --- | --- |
| **χ_lf_** | 0.31 | **0.80** | -0.12 | 0.17 |
| **χ_fd_** | 0.35 | **0.76** | 0.08 | 0.05 |
| **χ_fd_%** | 0.26 | 0.31 | 0.25 | -0.23 |
| **χ_ARM_** | -0.02 | **0.80** | 0.02 | 0.12 |
| **SIRM** | 0.29 | **0.87** | -0.08 | 0.21 |
| **χ_ARM_/SIRM** | -0.65 | -0.55 | 0.04 | 0.02 |
| **χ_ARM_/χ_lf_** | -0.64 | -0.45 | 0.06 | 0.17 |
| **S_20_** | 0.04 | **0.64** | -0.01 | 0.25 |
| **S-ratio** | -0.13 | 0.19 | -0.12 | 0.35 |
| **HIRM** | 0.29 | **0.87** | -0.08 | 0.22 |
| **Ag/Al** | **0.64** | -0.14 | **0.69** | 0.14 |
| **Ba/Al** | **0.60** | 0.14 | 0.46 | -0.20 |
| **Ca/Al** | 0.52 | -0.05 | -0.55 | -0.29 |
| **Cd/Al** | **0.78** | -0.15 | -0.29 | -0.05 |
| **Co/Al** | **0.87** | -0.29 | 0.10 | 0.34 |
| **Cr/Al** | **0.79** | -0.18 | 0.48 | 0.16 |
| **Cu/Al** | **0.77** | -0.26 | -0.48 | 0.20 |
| **Fe/Al** | **0.63** | -0.16 | **0.64** | 0.24 |
| **K/Al** | **0.71** | -0.12 | -0.41 | -0.19 |
| **Li/Al** | **0.76** | -0.09 | -0.43 | -0.22 |
| **Mg/Al** | **0.74** | 0.01 | 0.07 | -0.14 |
| **Mn/Al** | **0.78** | -0.04 | -0.25 | 0.02 |
| **Na/Al** | -0.55 | -0.21 | -0.53 | 0.09 |
| **Ni/Al** | 0.57 | -0.42 | -0.35 | 0.55 |
| **Pb/Al** | -0.69 | -0.10 | 0.54 | 0.27 |
| **Sr/Al** | 0.58 | -0.15 | -0.30 | 0.14 |
| **Zn/Al** | -0.46 | 0.13 | 0.03 | -0.36 |
| **Zr/Al** | **0.73** | -0.32 | -0.05 | -0.10 |
| **Rb/Al** | 0.37 | -0.09 | -0.04 | -0.56 |
| **V/Al** | 0.57 | -0.19 | 0.56 | -0.15 |
| **Ti/Al** | 0.53 | -0.19 | 0.56 | -0.16 |
| **TOC %** | 0.04 | -0.41 | -0.09 | **0.75** |
| **Eigenvalue** | **10.51** | **5.34** | **3.91** | **2.36** |
| **% variance** | **32.83** | **16.68** | **12.23** | **7.37** |


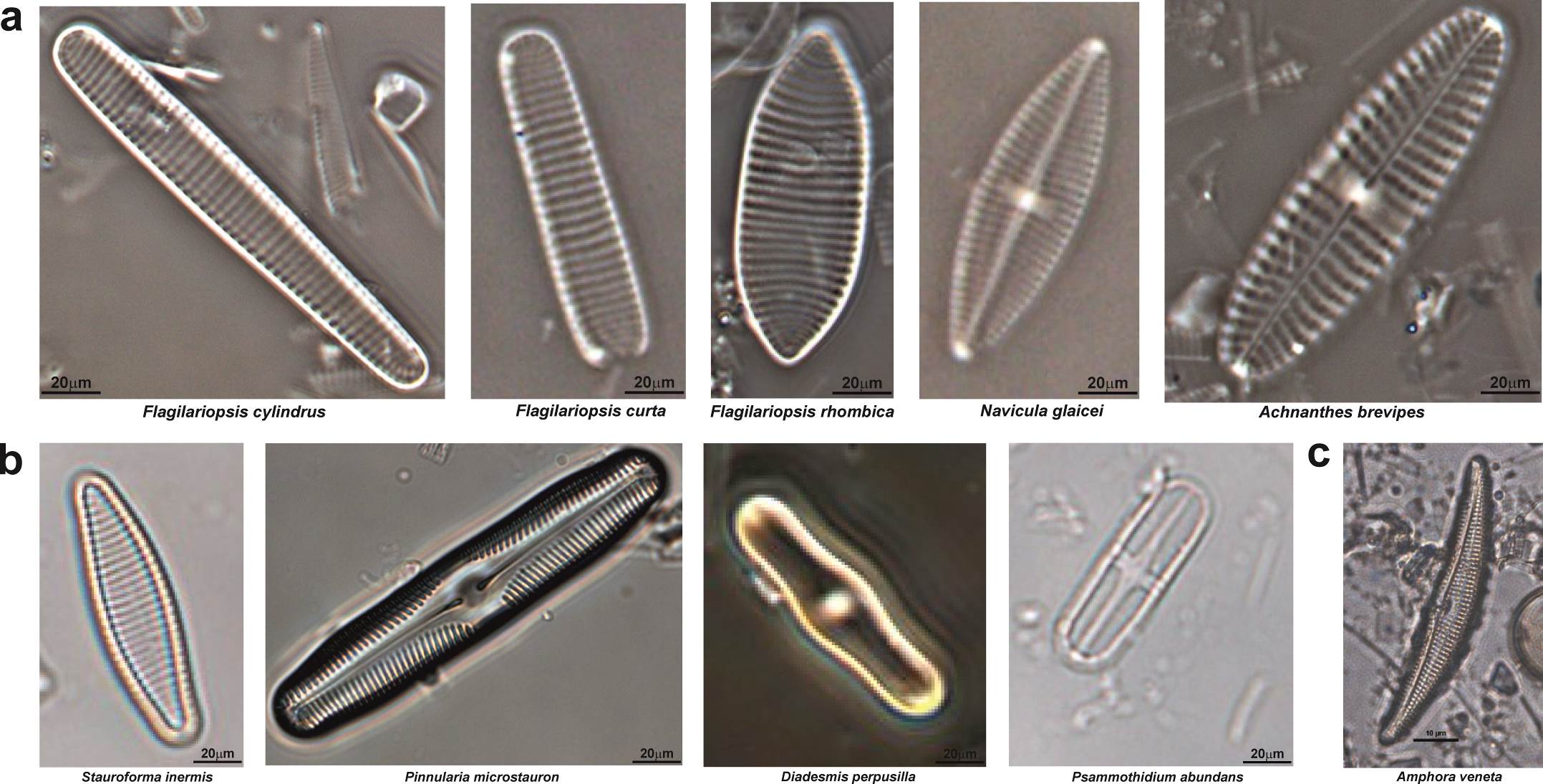


Supplementary Figure 1: Photographs of some diatom species in (a) marine environment; (b) freshwater environment and (c) transition zone from Heart Lake.


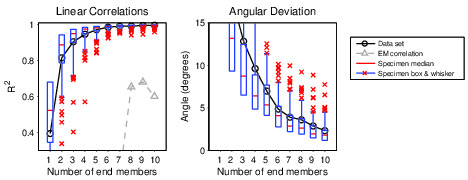


Supplementary Figure 2: End-member analysis (EMA) fitting statistics. Left; Correlation coefficient (R^2^; between observed and model grain size distribution) vs. number of EMs. Right: Angular deviation vs. number of EMs. In both left and right, solid black line and circles represent mean R^2^ and angular deviation, respectively. Blue boxes represent inter quartile range of R^2^ and angular deviation, with red line representing median R^2^ and angular deviation. The whiskers are one sided (95% confidence intervals of the fitting parameters, R^2^ and angular deviation).


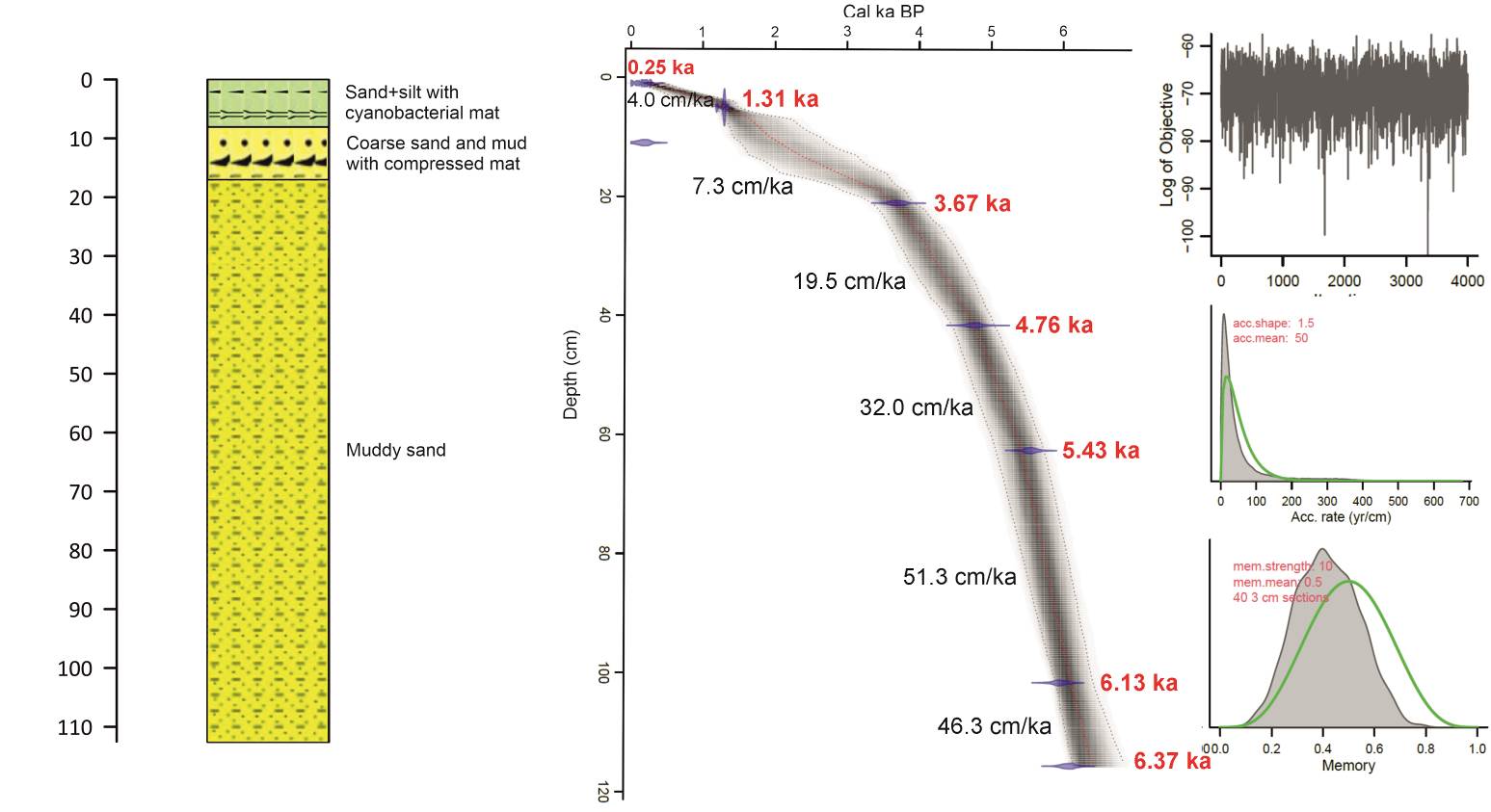


Supplementary Figure 3: Litholog of SL1 sediment core and age-depth model for SL1 sediment core reconstructed using BACON^1^ (version 3.0.0). Based on the ^14^C ages, the middle best estimate for calibrated age is shown (red dotted line), and the upper and lower values are shown in the grey band (95% confidence range; grey dashed line). Calibrated radiocarbon ages (text in red) and the sedimentation rates between these dates (text in black) are also given in figure.


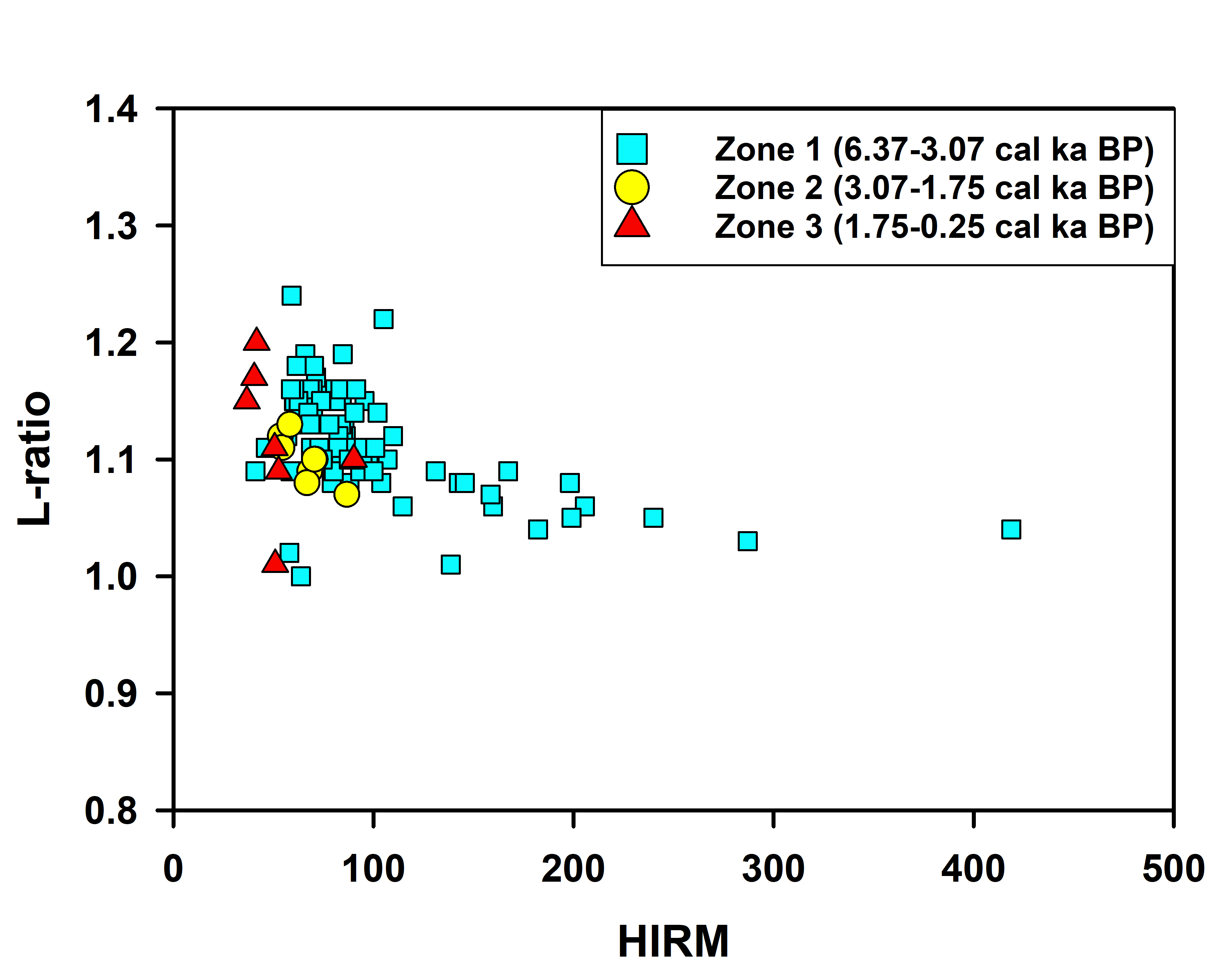


Supplementary Figure 4: Biplot of L-ratio and HIRM.


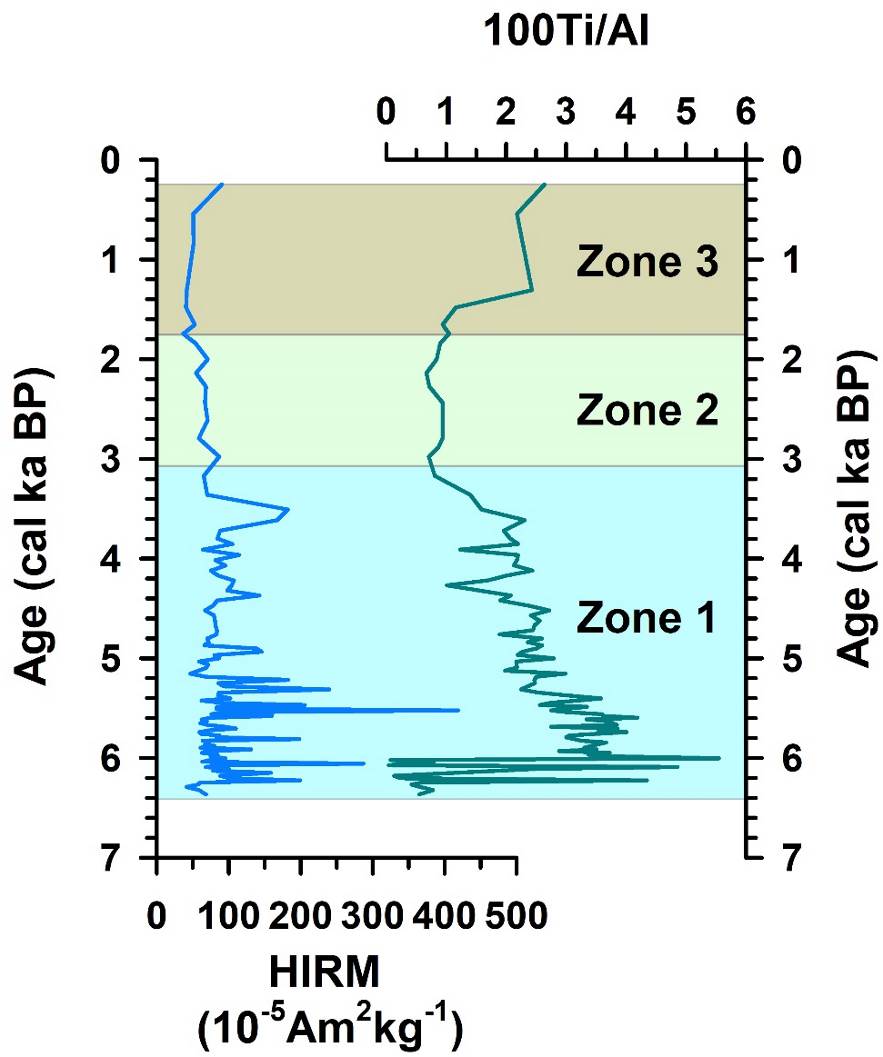


Supplementary Figure 5: Downcore variations in HIRM and the ratio 100Ti/Al.


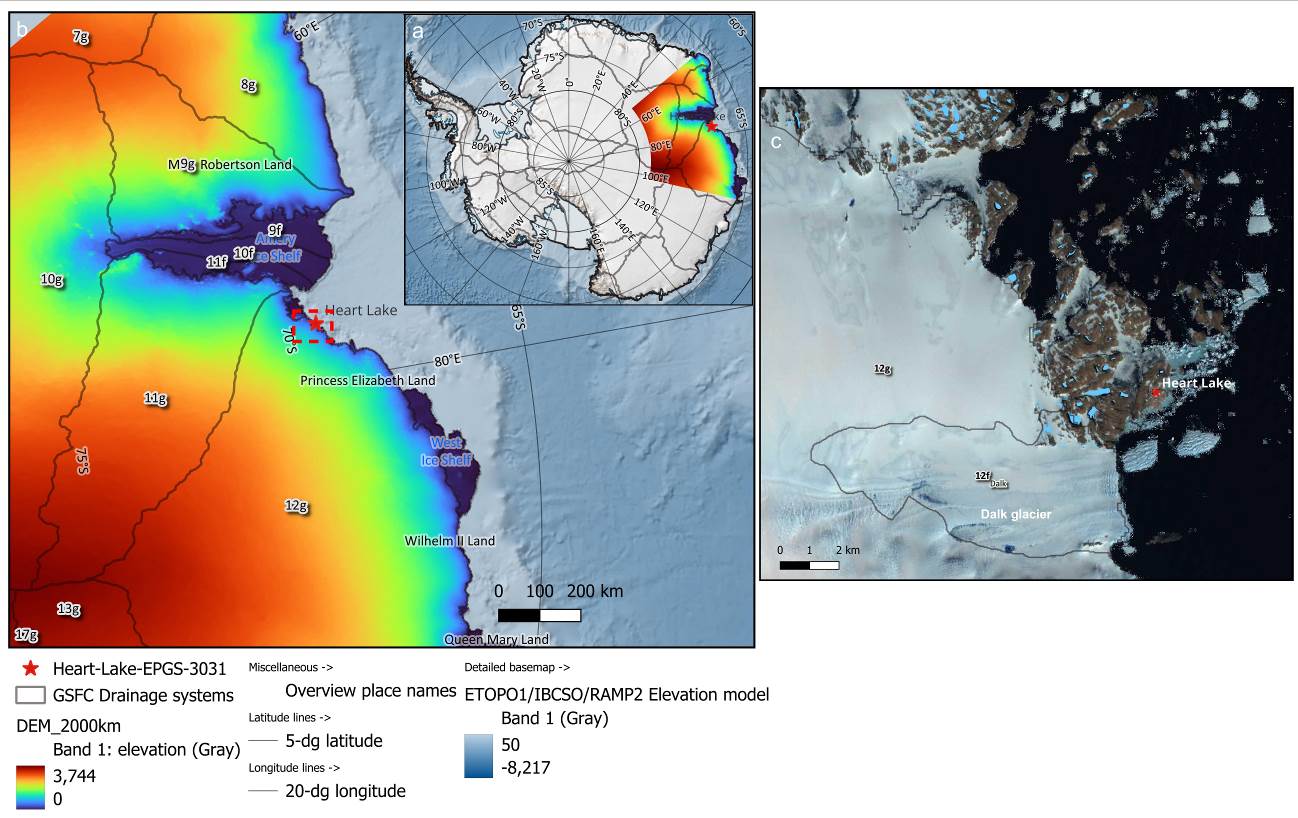


Supplementary Figure 6: (a) Antarctic continent with area of interest (East Antarctica). Solid grey lines represent NASA-Goddard Space Flight Center (GSFC) drainage boundaries^5^. The blue to red gradient shading represents the CryoSat-2 satellite radar altimeter based digital elevation model^6^ (1 km spatial resolution); (b) a zoomed in view of elevation variation in the eastern continental margin of Antarctica including study area, Lambert glacier, Amry Ice Shelf, Larsemann Hill region, drainage boundaries (7g to 17g) and Radarsat Antarctic Mapping Project Digital Elevation Model and bathymetry^7^. Both elevation and bathymetry are in meters (m); (c) A zoomed in satellite photograph (Landsat Image Mosaic of Antarctica; LIMA) of red rectangular insert in ‘b’ including Dalk Glacier, Broknes Peninsula, local drainage boundaries and Heart Lake core site. The figure was made using QGIS (version 3.40.4, https://qgis.org/) and Quantarctica package^8^ (version 3.2, https://npolar.no/quantarctica/). The digital elevation data was downloaded using google earth engine (URL: https://code.earthengine.google.com/). The GEE data ID is "ee.Image("CPOM/CryoSat2/ANTARCTICA_DEM")".


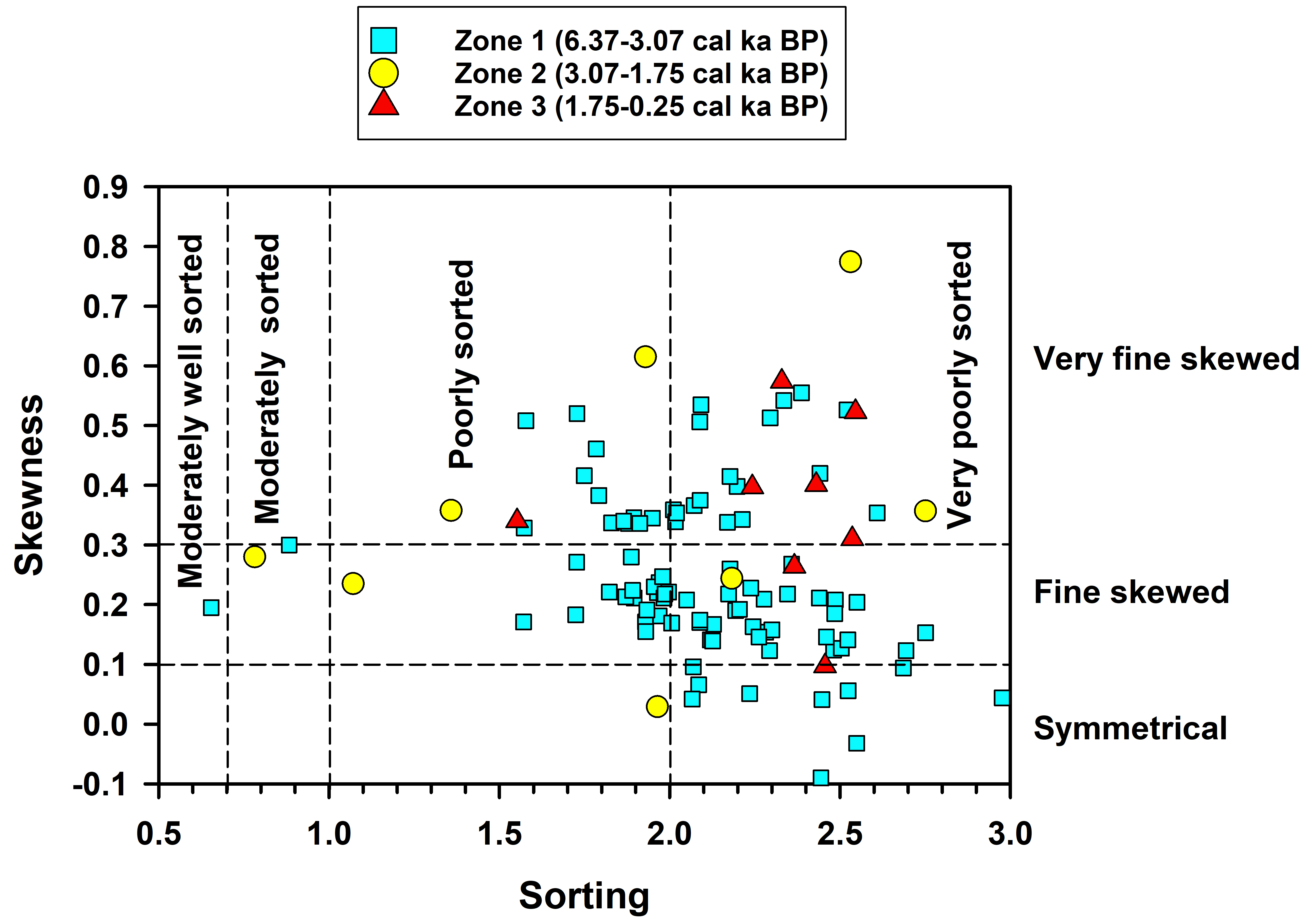


Supplementary Figure 7: Biplot of skewness and sorting.


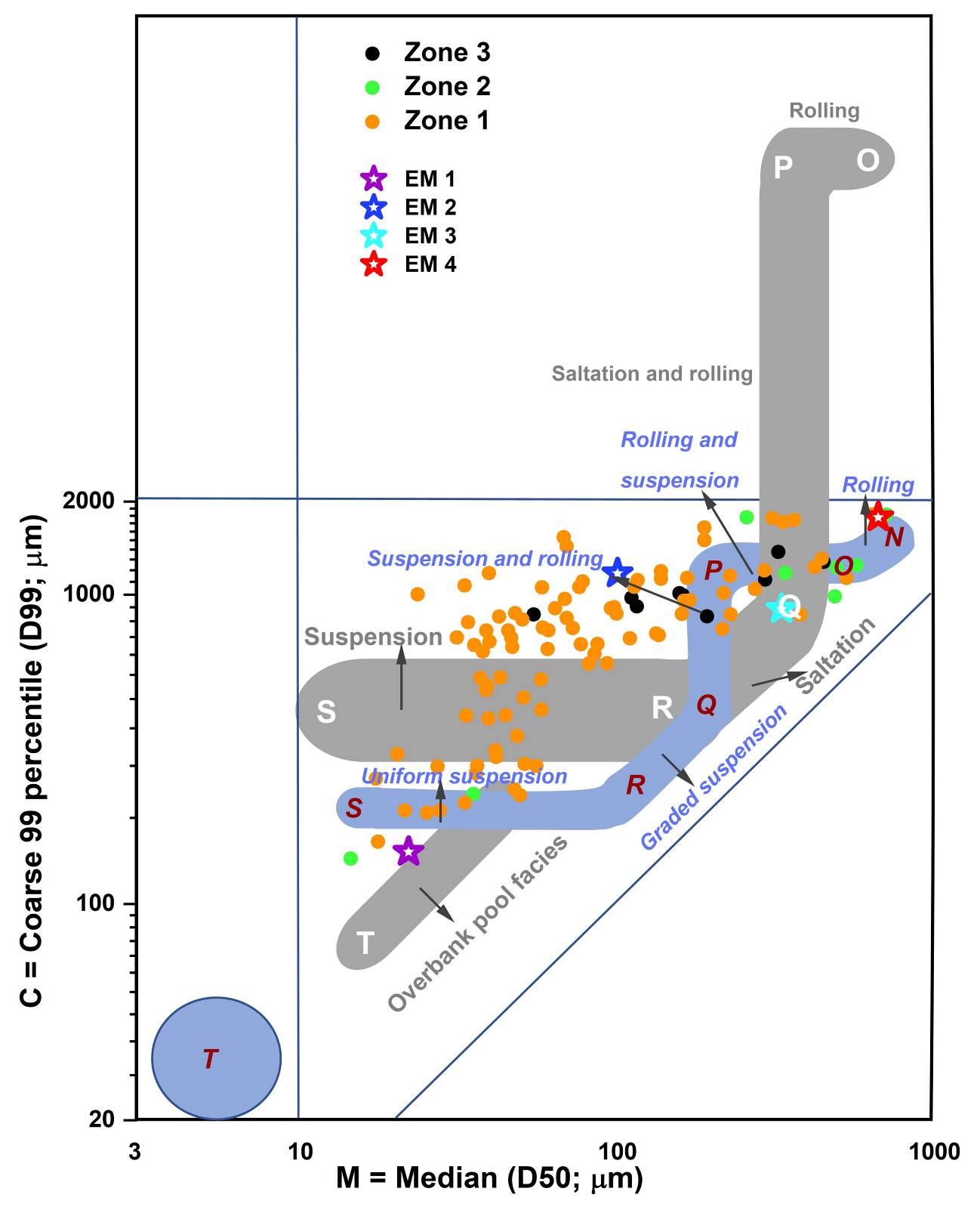


Supplementary Figure 8: C-M plot for Heart Lake sediments (After ref.^9^). The grey field is modified after ref.^9^ and the blue-grey field is modified after ref.^10^.


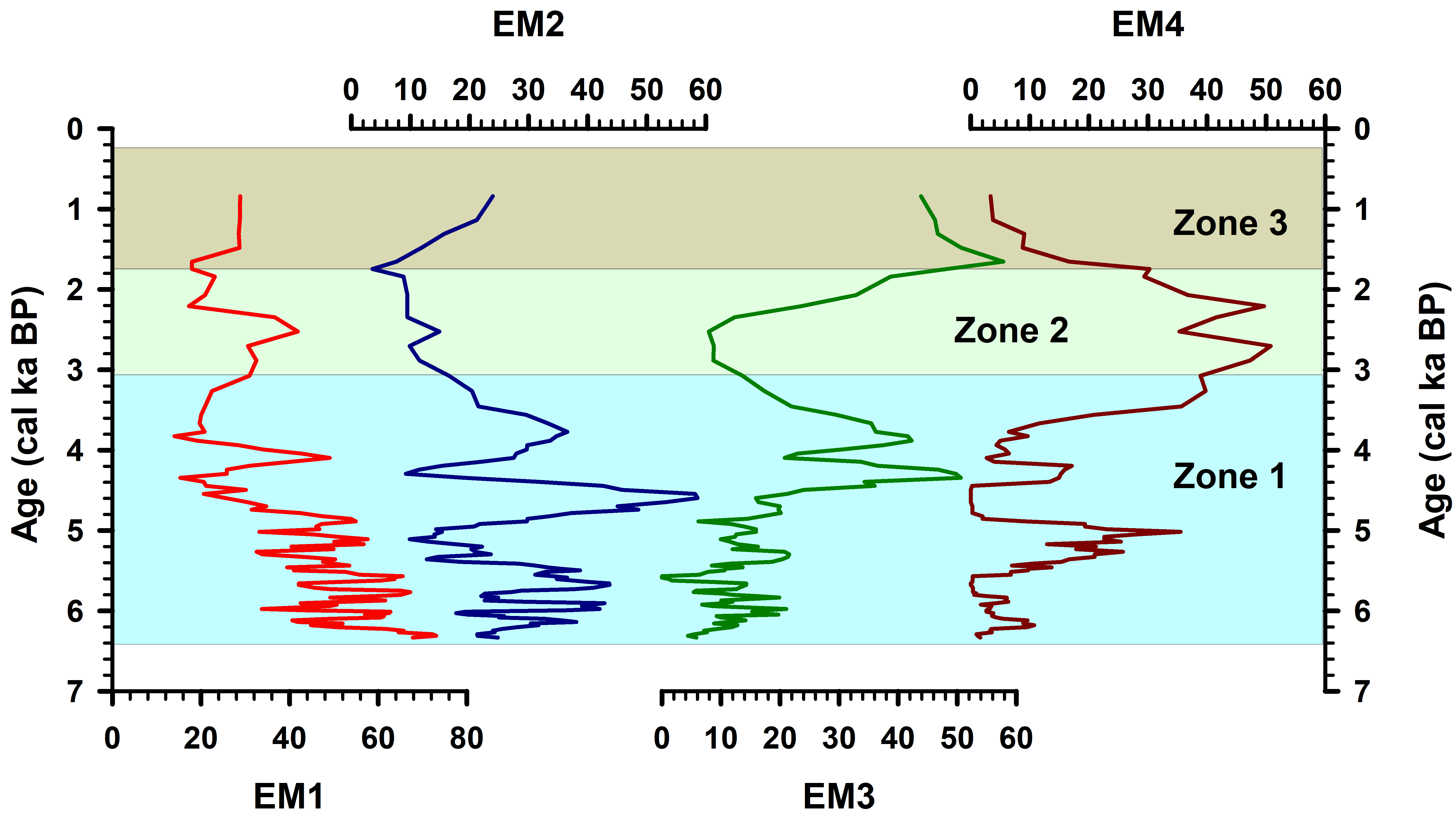


Supplementary Figure 9: Downcore variations in the end-member abundances for Heart Lake sediment core.


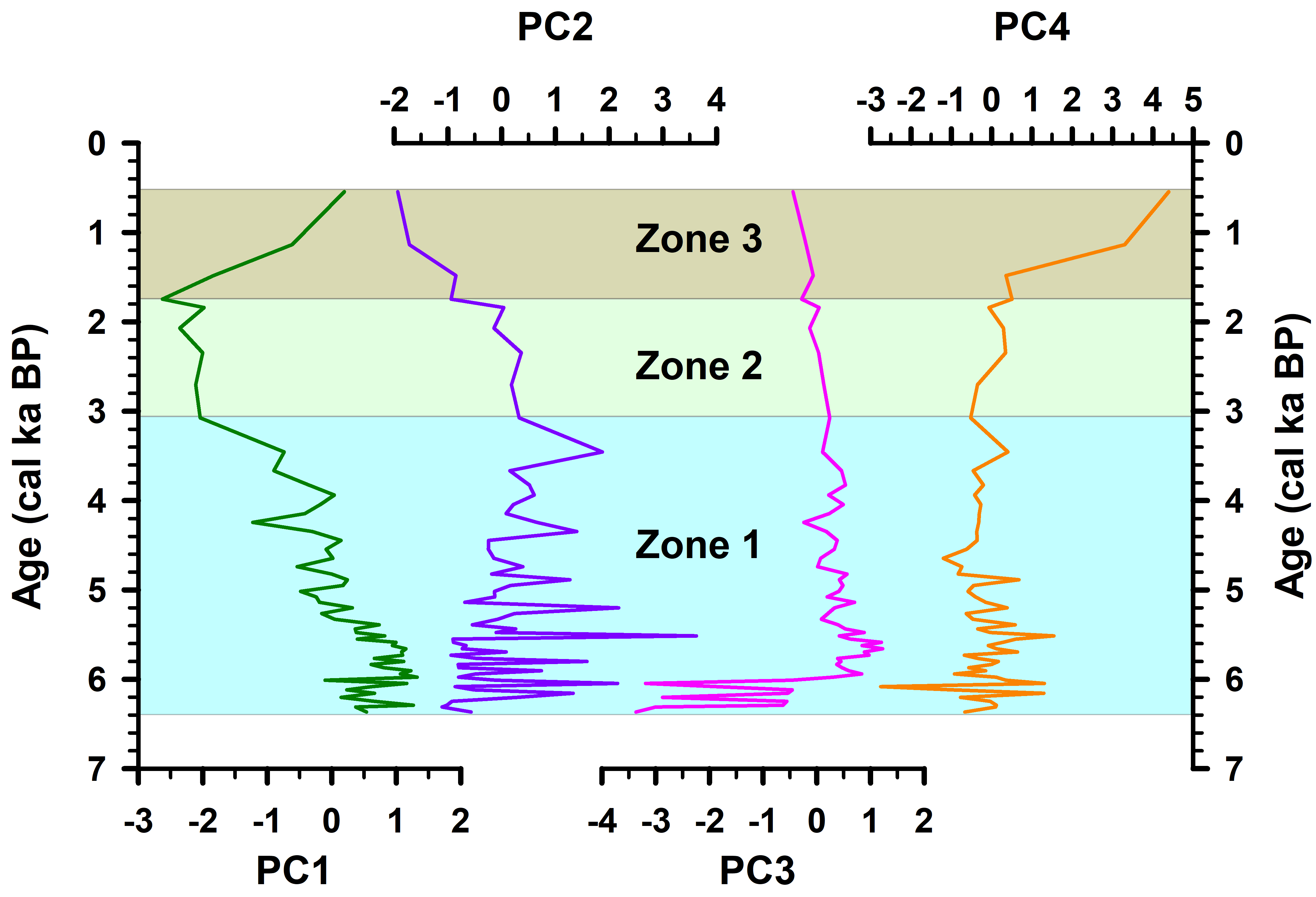


Supplementary Figure 10: Downcore variations in the significant principal components.


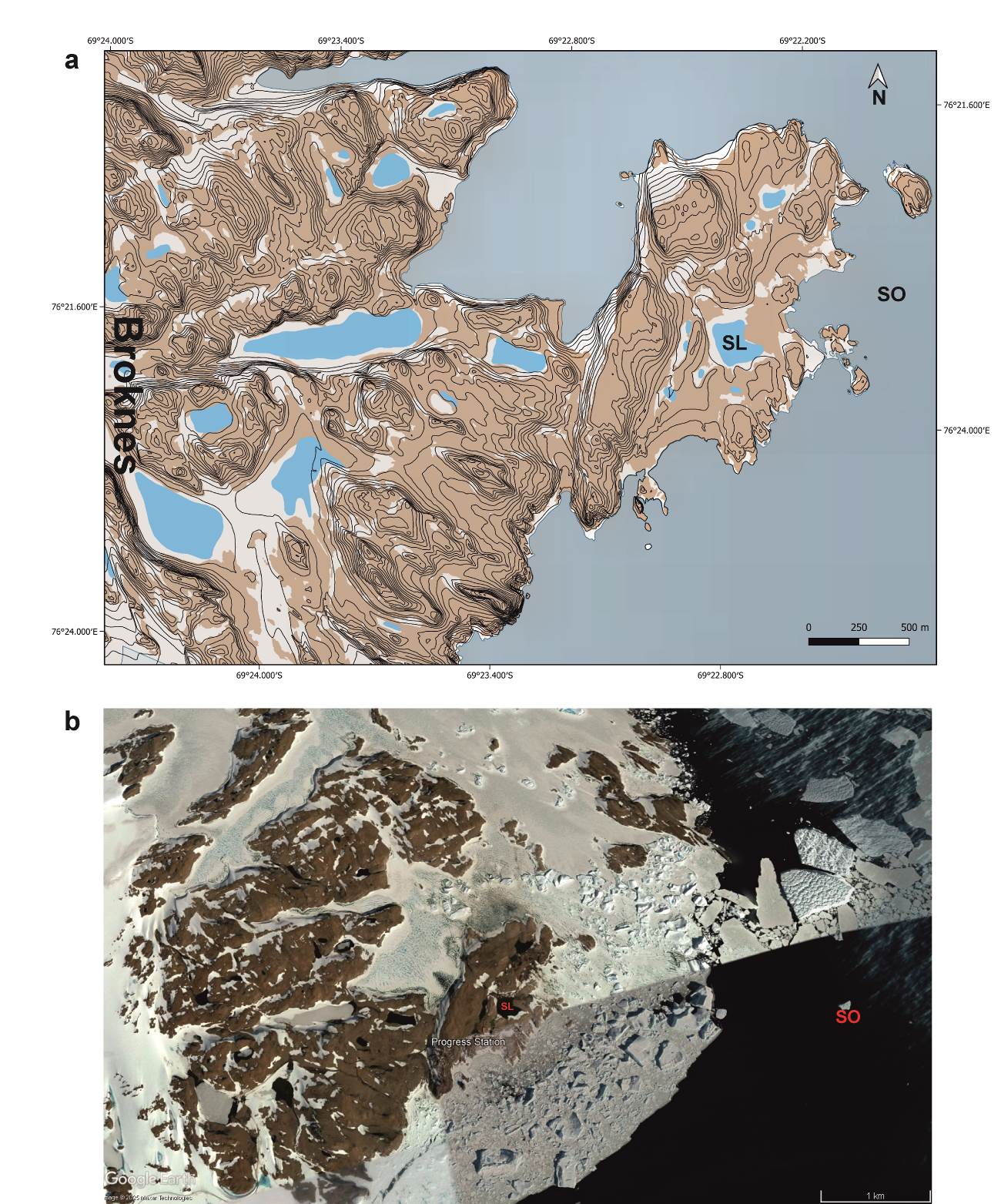


Supplementary Figure 11: (a) Contour map of the region surrounding Heart Lake, generated using the software Quantarctica^8^, a free GIS for Antarctica (version 3.2, https://npolar.no/quantarctica/) (b) aerial photograph of the region surrounding Heart Lake (obtained from Google Earth, https://earth.google.com/web/). SL represents Stepped Lake (Heart Lake) and SO represents Southern Ocean.

**References**

1. Blaauw, M. & Christen, J. A. Flexible paleoclimate age-depth models using an autoregressive gamma process. *Bayesian Anal.* **6**, 457–474 (2011).
2. R Core Team. R: A Language and Environment for Statistical Computing. R Foundation for Statistical Computing, Vienna. https://www.R-project.org (2021).
3. Hogg, A. G. *et al.* SHCal20 Southern Hemisphere Calibration, 0–55,000 Years cal BP. *Radiocarbon* **62**, 759–778 (2020).
4. Heaton, T. J. *et al.* Marine20—The Marine Radiocarbon Age Calibration Curve (0-55,000 cal BP). *Radiocarbon* **62**, 779–820 (2020).
5. Slater, T. *et al.* A new digital elevation model of Antarctica derived from CryoSat-2 altimetry. *Cryosphere* **12**, 1551–1562 (2018).
6. Zwally, H. *et al.* Antarctic and Greenland Drainage Systems, GSFC Cryospheric Sciences Laboratory, (2012) at http://icesat4.gsfc.nasa.gov/cryo_data/ant_grn_drainage_systems.php
7. Liu, H *et al.* Radarsat Antarctic Mapping Project Digital Elevation Model. (NSIDC-0082, Version 2). (2015) [Data Set]. Boulder, Colorado USA. NASA National Snow and Ice Data Center Distributed Active Archive Center. https://doi.org/10.5067/8JKNEW6BFRVD.
8. Matsuoka, K. *et al.* Quantarctica, an integrated mapping environment for Antarctica, the Southern Ocean, and sub-Antarctic islands. *Environ. Model. Softw.* **140**, 105015 (2021).
9. Passega, R. Texture as characteristic of clastic deposition. *Am. Assoc. Pet. Geol. Bull.* **41**, 1952–1984 (1957).
10. Ludwikowska-Kędzia, M. *Ewolucja Środkowego Odcinka Doliny Rzeki Belnianki w Późnym Glacjale i Holocenie*. (Wydaw. Akademickie Dialog, 2000).
