# Supplementary material for: Environmental evolution of a coastal lake in the Larsemann Hills, East Antarctica during the Holocene: a multi-proxy perspective
Source: Sci Rep. 2026 Feb 15;16:9139. doi: 10.1038/s41598-026-39218-8 (PMC12996284; doi:10.1038/s41598-026-39218-8)
Supplement: Supplementary file 2 — Supplementary Material 2 [file 41598_2026_39218_MOESM2_ESM.docx]

Extended Data Table 1: Environmental magnetic data for SL1 sediment core.

| **Age (cal ka BP)** | **χ_lf_ (10^-8^m^3^kg-^1^)** | | **χ_fd_%** | **χ_ARM_ (10^-5^m^3^kg-^1^)** | | **SIRM (10^-5^Am^2^kg^-1^)** | **χ_ARM_/SIRM (10^-5^mA^-1^)** | **χ_ARM_ /χ_lf_** | **S_20_** | **S-ratio** | **HIRM** | **L-ratio** |  |
| --- | --- | --- | --- | --- | --- | --- | --- | --- | --- | --- | --- | --- | --- |
|  |  |  |  |  |  |  |  |  |  |  |  |  |  |
| 0.25 | 41.61 | | 0.00 | 0.028 | | 90.13 | 31.00 | 0.67 | 0.27 | 1.00 | 90.13 | 1.10 |  |
| 0.54 | 26.75 | | 0.00 | 0.023 | | 50.53 | 45.62 | 0.86 | 0.24 | 1.00 | 50.53 | 1.11 |  |
| 0.84 | 24.58 | | 0.00 | 0.029 | | 50.84 | 56.51 | 1.17 | 0.23 | 1.00 | 50.84 | 1.01 |  |
| 1.31 | 48.95 | | 0.00 | 0.023 | | 42.73 | 54.06 | 0.47 | 0.19 | 0.95 | 41.60 | 1.20 |  |
| 1.48 | 31.66 | | 0.00 | 0.026 | | 41.35 | 63.88 | 0.83 | 0.17 | 0.95 | 40.32 | 1.17 |  |
| 1.65 | 24.90 | | 0.00 | 0.027 | | 54.43 | 49.99 | 1.09 | 0.17 | 0.93 | 52.60 | 1.09 |  |
| 1.75 | 28.15 | 0.00 | | 0.032 | | 36.63 | 87.04 | 1.13 | 0.17 | 1.00 | 36.63 | 1.15 |  |
| 1.84 | 51.21 | 6.25 | | 0.034 | | 53.57 | 63.67 | 0.67 | 0.20 | 1.00 | 53.49 | 1.12 |  |
| 2.00 | 48.29 | 6.90 | | 0.037 | | 71.55 | 51.32 | 0.76 | 0.22 | 0.98 | 70.82 | 1.10 |  |
| 2.14 | 39.22 | 3.03 | | 0.035 | | 54.48 | 63.52 | 0.88 | 0.22 | 0.99 | 54.25 | 1.11 |  |
| 2.28 | 53.73 | 3.70 | | 0.033 | | 68.18 | 48.78 | 0.62 | 0.31 | 1.00 | 68.18 | 1.09 |  |
| 2.43 | 61.85 | 5.88 | | 0.031 | | 66.74 | 46.56 | 0.50 | 0.23 | 1.00 | 66.74 | 1.08 |  |
| 2.61 | 62.66 | 1.59 | | 0.030 | | 71.02 | 42.73 | 0.48 | 0.30 | 0.98 | 70.42 | 1.10 |  |
| 2.79 | 53.20 | 1.59 | | 0.031 | | 59.05 | 52.29 | 0.58 | 0.25 | 0.97 | 58.17 | 1.13 |  |
| 2.98 | 79.22 | 1.52 | | 0.031 | | 86.64 | 35.22 | 0.39 | 0.24 | 1.00 | 86.64 | 1.07 |  |
| 3.17 | 59.74 | 3.57 | | 0.036 | | 65.46 | 54.87 | 0.60 | 0.20 | 0.99 | 65.17 | 1.09 |  |
| 3.36 | 76.49 | 1.85 | | 0.027 | | 70.63 | 38.36 | 0.35 | 0.25 | 0.98 | 69.99 | 1.10 |  |
| 3.51 | 296.30 | 1.56 | | 0.042 | | 184.57 | 22.52 | 0.14 | 0.26 | 0.97 | 182.17 | 1.04 |  |
| 3.61 | 195.38 | 2.17 | | 0.040 | 167.02 | | 24.00 | 0.21 | 0.30 | 1.00 | 167.23 | 1.09 |  |
| 3.72 | 88.36 | 2.04 | | 0.031 | 92.35 | | 33.74 | 0.35 | 0.20 | 0.91 | 87.98 | 1.08 |  |
| 3.80 | 92.17 | 1.64 | | 0.030 | 84.10 | | 35.86 | 0.33 | 0.25 | 1.00 | 84.10 | 1.15 |  |
| 3.85 | 131.00 | 4.00 | | 0.040 | 114.21 | | 34.91 | 0.30 | 0.26 | 0.84 | 105.01 | 1.22 |  |
| 3.91 | 122.03 | 1.89 | | 0.014 | 65.18 | | 22.19 | 0.12 | 0.33 | 0.95 | 63.70 | 1.00 |  |
| 3.96 | 148.46 | 4.04 | | 0.036 | 122.47 | | 29.68 | 0.24 | 0.24 | 0.87 | 114.54 | 1.06 |  |
| 4.02 | 83.06 | 6.06 | | 0.032 | 82.62 | | 38.87 | 0.39 | 0.18 | 0.96 | 81.07 | 1.15 |  |
| 4.07 | 77.03 | 5.13 | | 0.026 | 96.05 | | 26.98 | 0.34 | 0.20 | 0.99 | 95.42 | 1.15 |  |
| **Age (cal ka BP)** | **χ_lf_ (10^-8^m^3^kg-^1^)** | **χ_fd_%** | | **χ_ARM_ (10^-5^m^3^kg-^1^)** | **SIRM (10^-5^Am^2^kg^-1^)** | | **χ_ARM_/SIRM (10^-5^mA^-1^)** | **χ_ARM_ /χ_lf_** | **S_20_** | **S-ratio** | **HIRM** | **L-ratio** |  |
| 4.12 | 53.87 | 0.00 | | 0.028 | 77.17 | | 36.08 | 0.52 | 0.19 | 0.94 | 74.94 | 1.16 |  |
| 4.17 | 71.98 | 4.76 | | 0.031 | 87.04 | | 35.80 | 0.43 | 0.21 | 0.95 | 85.01 | 1.13 |  |
| 4.22 | 78.23 | 3.92 | | 0.036 | 107.45 | | 33.04 | 0.45 | 0.20 | 0.99 | 106.97 | 1.10 |  |
| 4.27 | 70.62 | 3.23 | | 0.035 | 105.65 | | 32.70 | 0.49 | 0.26 | 0.97 | 103.83 | 1.08 |  |
| 4.32 | 77.43 | 6.76 | | 0.033 | 98.88 | | 33.56 | 0.43 | 0.23 | 0.97 | 97.51 | 1.10 |  |
| 4.37 | 152.94 | 5.93 | | 0.034 | 145.30 | | 23.17 | 0.22 | 0.34 | 0.96 | 142.52 | 1.08 |  |
| 4.42 | 82.56 | 7.69 | | 0.032 | 85.24 | | 37.56 | 0.39 | 0.23 | 0.96 | 83.75 | 1.10 |  |
| 4.47 | 90.00 | 1.49 | | 0.029 | 80.63 | | 35.44 | 0.32 | 0.19 | 0.94 | 78.40 | 1.16 |  |
| 4.52 | 72.07 | 4.44 | | 0.022 | 69.37 | | 32.43 | 0.31 | 0.16 | 0.93 | 66.80 | 1.14 |  |
| 4.57 | 67.06 | 5.36 | | 0.028 | 85.05 | | 33.16 | 0.42 | 0.18 | 0.86 | 79.18 | 1.08 |  |
| 4.62 | 94.33 | 2.38 | | 0.031 | 83.09 | | 36.74 | 0.32 | 0.18 | 0.94 | 80.80 | 1.16 |  |
| 4.67 | 85.38 | 2.82 | | 0.031 | 84.69 | | 36.62 | 0.36 | 0.17 | 0.93 | 81.56 | 1.11 |  |
| 4.72 | 77.14 | 8.33 | | 0.030 | 84.83 | | 35.40 | 0.39 | 0.18 | 0.97 | 83.43 | 1.16 |  |
| 4.76 | 74.74 | 8.33 | | 0.033 | 84.67 | | 39.56 | 0.45 | 0.21 | 0.96 | 82.94 | 1.12 |  |
| 4.80 | 74.07 | 5.17 | | 0.031 | 72.27 | | 43.46 | 0.42 | 0.18 | 0.93 | 69.75 | 1.14 |  |
| 4.84 | 78.15 | 5.56 | | 0.031 | 75.35 | | 40.83 | 0.39 | 0.16 | 0.91 | 72.01 | 1.10 |  |
| 4.87 | 65.47 | 7.14 | | 0.027 | 67.55 | | 39.43 | 0.41 | 0.18 | 0.95 | 65.85 | 1.19 |  |
| 4.90 | 184.77 | 7.07 | | 0.032 | 138.58 | | 22.79 | 0.17 | 0.31 | 1.00 | 138.58 | 1.01 |  |
| 4.94 | 170.86 | 3.03 | | 0.030 | 146.80 | | 20.73 | 0.18 | 0.34 | 0.98 | 145.60 | 1.08 |  |
| 4.97 | 93.58 | 5.66 | | 0.030 | 81.26 | | 36.79 | 0.32 | 0.25 | 0.96 | 79.77 | 1.09 |  |
| 5.00 | 113.94 | 6.03 | | 0.030 | 87.72 | | 34.03 | 0.26 | 0.23 | 0.97 | 86.46 | 1.11 |  |
| 5.03 | 60.82 | 2.08 | | 0.020 | 59.66 | | 33.31 | 0.33 | 0.24 | 0.96 | 58.48 | 1.09 |  |
| 5.06 | 95.04 | 5.62 | | 0.030 | 72.64 | | 40.80 | 0.31 | 0.25 | 0.96 | 71.29 | 1.17 |  |
| 5.09 | 71.67 | 3.03 | | 0.027 | 71.35 | | 37.88 | 0.38 | 0.24 | 0.93 | 68.81 | 1.10 |  |
| 5.12 | 59.16 | 3.51 | | 0.024 | 59.81 | | 40.67 | 0.41 | 0.27 | 0.94 | 58.00 | 1.02 |  |
| 5.16 | 41.94 | 6.45 | | 0.023 | 47.16 | | 49.79 | 0.56 | 0.22 | 0.96 | 46.14 | 1.11 |  |
| 5.19 | 63.88 | 5.00 | | 0.029 | 70.43 | | 41.04 | 0.45 | 0.21 | 0.95 | 68.63 | 1.11 |  |
| 5.22 | 314.15 | 3.98 | | 0.049 | 187.07 | | 26.23 | 0.16 | 0.34 | 0.95 | 182.27 | 1.04 |  |
| 5.25 | 85.60 | 2.38 | | 0.031 | 87.47 | | 35.30 | 0.36 | 0.22 | 0.95 | 85.42 | 1.13 |  |
|  |  |  | |  |  | |  |  |  |  |  |  |  |
| **Age (cal ka BP)** | **χ_lf_ (10^-8^m^3^kg-^1^)** | **χ_fd_%** | | **χ_ARM_ (10^-5^m^3^kg-^1^)** | **SIRM (10^-5^Am^2^kg^-1^)** | | **χ_ARM_/SIRM (10^-5^mA^-1^)** | **χ_ARM_ /χ_lf_** | **S_20_** | **S-ratio** | **HIRM** | **L-ratio** |  |
| 5.28 | 77.40 | 6.67 | | 0.032 | 95.42 | | 33.35 | 0.41 | 0.20 | 0.95 | 92.95 | 1.11 |  |
| 5.31 | 204.23 | 1.06 | | 0.044 | 239.73 | | 18.40 | 0.22 | 0.38 | 1.00 | 239.83 | 1.05 |  |
| 5.34 | 84.34 | 4.17 | | 0.031 | 88.00 | | 35.47 | 0.37 | 0.20 | 0.96 | 86.04 | 1.12 |  |
| 5.38 | 71.64 | 5.80 | | 0.029 | 84.78 | | 34.61 | 0.41 | 0.23 | 1.00 | 84.78 | 1.12 |  |
| 5.40 | 96.17 | 2.70 | | 0.029 | 102.56 | | 28.46 | 0.30 | 0.17 | 0.99 | 102.07 | 1.14 |  |
| 5.43 | 56.42 | 7.14 | | 0.029 | 64.10 | | 44.65 | 0.51 | 0.19 | 0.93 | 61.76 | 1.13 |  |
| 5.45 | 90.10 | 8.33 | | 0.032 | 95.61 | | 32.98 | 0.35 | 0.22 | 0.95 | 93.37 | 1.09 |  |
| 5.47 | 214.93 | 5.21 | | 0.040 | 209.51 | | 19.22 | 0.19 | 0.24 | 0.96 | 205.66 | 1.06 |  |
| 5.49 | 101.12 | 3.49 | | 0.032 | 87.06 | | 36.90 | 0.32 | 0.21 | 0.93 | 83.84 | 1.13 |  |
| 5.51 | 94.80 | 1.09 | | 0.031 | 85.02 | | 35.97 | 0.32 | 0.17 | 0.94 | 82.49 | 1.12 |  |
| 5.52 | 250.19 | 7.58 | | 0.072 | 423.37 | | 16.96 | 0.29 | 0.22 | 0.98 | 418.87 | 1.04 |  |
| 5.54 | 73.19 | 1.52 | | 0.034 | 90.48 | | 37.89 | 0.47 | 0.20 | 1.00 | 90.48 | 1.14 |  |
| 5.56 | 69.30 | 0.00 | | 0.029 | 77.72 | | 36.96 | 0.41 | 0.16 | 0.95 | 75.59 | 1.11 |  |
| 5.58 | 101.53 | 8.97 | | 0.041 | 164.34 | | 24.75 | 0.40 | 0.27 | 0.94 | 159.76 | 1.06 |  |
| 5.60 | 72.20 | 4.26 | | 0.026 | 75.98 | | 34.25 | 0.36 | 0.17 | 0.94 | 73.88 | 1.15 |  |
| 5.61 | 74.66 | 0.00 | | 0.029 | 63.74 | | 45.44 | 0.39 | 0.15 | 0.95 | 62.00 | 1.18 |  |
| 5.63 | 67.73 | 6.67 | | 0.022 | 71.57 | | 30.61 | 0.32 | 0.19 | 0.95 | 69.60 | 1.16 |  |
| 5.65 | 78.42 | 0.00 | | 0.022 | 63.17 | | 34.22 | 0.28 | 0.13 | 0.90 | 60.11 | 1.15 |  |
| 5.67 | 73.84 | 9.52 | | 0.032 | 72.65 | | 43.78 | 0.43 | 0.15 | 0.94 | 70.30 | 1.18 |  |
| 5.69 | 176.91 | 5.65 | | 0.029 | 98.15 | | 29.47 | 0.16 | 0.29 | 0.93 | 94.58 | 1.10 |  |
| 5.70 | 103.04 | 8.33 | | 0.042 | 109.77 | | 38.49 | 0.41 | 0.20 | 1.00 | 109.77 | 1.12 |  |
| 5.72 | 82.98 | 8.89 | | 0.035 | 74.93 | | 46.74 | 0.42 | 0.16 | 0.94 | 72.70 | 1.15 |  |
| 5.74 | 74.81 | 8.00 | | 0.028 | 61.32 | | 46.09 | 0.38 | 0.11 | 0.93 | 59.10 | 1.24 |  |
| 5.76 | 97.90 | 8.33 | | 0.032 | 62.77 | | 51.60 | 0.33 | 0.12 | 0.96 | 61.57 | 1.18 |  |
| 5.78 | 89.88 | 0.00 | | 0.027 | 88.73 | | 30.61 | 0.30 | 0.20 | 0.96 | 87.07 | 1.11 |  |
| 5.79 | 92.50 | 4.69 | | 0.029 | 83.85 | | 34.21 | 0.31 | 0.22 | 0.96 | 82.25 | 1.11 |  |
| 5.81 | 173.95 | 7.03 | | 0.040 | 198.50 | | 20.30 | 0.23 | 0.36 | 1.00 | 198.20 | 1.08 |  |
| 5.83 | 65.43 | 8.57 | | 0.024 | 65.45 | | 36.67 | 0.37 | 0.19 | 0.94 | 63.41 | 1.14 |  |
| **Age (cal ka BP)** | **χ_lf_ (10^-8^m^3^kg-^1^)** | **χ_fd_%** | | **χ_ARM_ (10^-5^m^3^kg-^1^)** | **SIRM (10^-5^Am^2^kg^-1^)** | | **χ_ARM_/SIRM (10^-5^mA^-1^)** | **χ_ARM_ /χ_lf_** | **S_20_** | **S-ratio** | **HIRM** | **L-ratio** |  |
| 5.84 | 95.40 | 3.90 | | 0.026 | 75.14 | | 34.45 | 0.27 | 0.23 | 0.94 | 72.81 | 1.11 |  |
| 5.86 | 81.48 | 10.91 | | 0.026 | 68.35 | | 38.01 | 0.32 | 0.20 | 0.94 | 66.24 | 1.15 |  |
| 5.88 | 63.45 | 2.50 | | 0.028 | 83.28 | | 33.89 | 0.44 | 0.20 | 0.92 | 80.01 | 1.09 |  |
| 5.90 | 68.31 | 9.52 | | 0.025 | 62.83 | | 39.24 | 0.36 | 0.15 | 0.92 | 60.39 | 1.15 |  |
| 5.91 | 166.24 | 6.45 | | 0.030 | 131.35 | | 22.94 | 0.18 | 0.32 | 1.00 | 131.08 | 1.09 |  |
| 5.93 | 82.62 | 5.56 | | 0.034 | 69.86 | | 48.87 | 0.41 | 0.15 | 0.95 | 68.06 | 1.16 |  |
| 5.95 | 79.37 | 10.00 | | 0.025 | 63.49 | | 38.76 | 0.31 | 0.17 | 0.97 | 62.48 | 1.15 |  |
| 5.97 | 88.28 | 10.42 | | 0.030 | 91.40 | | 32.97 | 0.34 | 0.21 | 0.85 | 84.55 | 1.19 |  |
| 5.98 | 121.96 | 1.82 | | 0.023 | 76.63 | | 29.99 | 0.19 | 0.22 | 0.95 | 74.68 | 1.10 |  |
| 6.00 | 80.04 | 4.00 | | 0.032 | 96.20 | | 33.11 | 0.40 | 0.24 | 0.98 | 95.06 | 1.11 |  |
| 6.02 | 87.64 | 2.22 | | 0.028 | 76.62 | | 36.47 | 0.32 | 0.18 | 0.92 | 73.75 | 1.15 |  |
| 6.04 | 69.90 | 5.26 | | 0.027 | 64.15 | | 41.71 | 0.38 | 0.18 | 0.96 | 62.95 | 1.13 |  |
| 6.06 | 266.18 | 4.29 | | 0.049 | 287.06 | | 16.97 | 0.18 | 0.32 | 1.00 | 287.06 | 1.03 |  |
| 6.07 | 89.73 | 6.67 | | 0.028 | 93.61 | | 29.91 | 0.31 | 0.22 | 0.95 | 91.32 | 1.16 |  |
| 6.09 | 80.25 | 1.96 | | 0.026 | 69.73 | | 37.71 | 0.33 | 0.14 | 0.94 | 67.50 | 1.14 |  |
| 6.11 | 81.07 | 7.14 | | 0.030 | 102.83 | | 29.25 | 0.37 | 0.25 | 0.94 | 99.98 | 1.09 |  |
| 6.13 | 57.53 | 3.03 | | 0.046 | 79.22 | | 58.61 | 0.81 | 0.16 | 0.96 | 77.81 | 1.13 |  |
| 6.15 | 344.33 | 0.44 | | 0.035 | 159.94 | | 21.81 | 0.10 | 0.32 | 0.98 | 158.43 | 1.07 |  |
| 6.18 | 107.47 | 2.50 | | 0.033 | 90.42 | | 36.85 | 0.31 | 0.24 | 0.94 | 87.60 | 1.10 |  |
| 6.20 | 122.80 | 4.17 | | 0.031 | 102.61 | | 30.53 | 0.26 | 0.21 | 0.97 | 100.82 | 1.11 |  |
| 6.22 | 206.15 | 4.04 | | 0.038 | 204.12 | | 18.71 | 0.19 | 0.27 | 0.95 | 198.93 | 1.05 |  |
| 6.25 | 66.23 | 2.70 | | 0.027 | 61.59 | | 44.00 | 0.41 | 0.16 | 0.96 | 60.35 | 1.16 |  |
| 6.27 | 70.49 | 5.41 | | 0.021 | 59.34 | | 35.42 | 0.30 | 0.21 | 0.92 | 56.86 | 1.12 |  |
| 6.29 | 38.18 | 7.69 | | 0.015 | 41.88 | | 36.49 | 0.40 | 0.29 | 0.96 | 40.98 | 1.09 |  |
| 6.32 | 73.79 | 2.70 | | 0.020 | 60.02 | | 34.12 | 0.28 | 0.16 | 0.95 | 58.64 | 1.16 |  |
| 6.37 | 103.77 | 5.36 | | 0.026 | 70.77 | | 36.78 | 0.25 | 0.16 | 0.93 | 68.44 | 1.13 |  |
