# Supplementary material for: Environmental evolution of a coastal lake in the Larsemann Hills, East Antarctica during the Holocene: a multi-proxy perspective
Source: Sci Rep. 2026 Feb 15;16:9139. doi: 10.1038/s41598-026-39218-8 (PMC12996284; doi:10.1038/s41598-026-39218-8)
Supplement: Supplementary file 3 — Supplementary Material 3 [file 41598_2026_39218_MOESM3_ESM.docx]

Extended Data Table 2: Metal concentration and weathering indices data for SL1 sediment core.

| **Age (cal ka BP)** | **Ag (ppm)** | **Al (ppm)** | **Ba (ppm)** | **Ca (ppm)** | **Cd (ppm)** | **Co (ppm)** | **Cr (ppm)** | **Cu (ppm)** | **Fe (ppm)** | **K (ppm)** | **Li (ppm)** | **Mg (ppm)** | **Mn (ppm)** | **Na (ppm)** |
| --- | --- | --- | --- | --- | --- | --- | --- | --- | --- | --- | --- | --- | --- | --- |
| 0.25 | 120.95 | 31901.55 | 196.51 | 8172.66 | 1.29 | 12.67 | 46.23 | 26.54 | 18619.02 | 11071.95 | 21.18 | 3931.34 | 326.71 | 8176.56 |
| 0.54 | 121.30 | 37236.15 | 267.85 | 9479.46 | 1.33 | 14.99 | 43.24 | 22.69 | 16805.87 | 15453.99 | 28.18 | 4019.01 | 301.20 | 10185.63 |
| 1.31 | 115.17 | 33253.17 | 173.53 | 7393.83 | 0.96 | 9.80 | 35.19 | 12.65 | 14100.50 | 16588.56 | 24.40 | 847.46 | 281.61 | 11365.94 |
| 1.48 | 79.22 | 50451.74 | 339.61 | 10245.18 | 0.57 | 6.18 | 23.36 | 8.57 | 10447.32 | 22595.51 | 34.84 | 2034.34 | 245.34 | 16196.17 |
| 1.65 | 37.29 | 30436.13 | 214.32 | 6560.18 | 0.42 | 2.75 | 13.16 | 4.14 | 6175.74 | 10334.73 | 16.59 | 1470.59 | 108.36 | 6362.84 |
| 1.75 | 50.84 | 36618.43 | 204.62 | 7370.36 | 0.33 | 3.27 | 13.45 | 2.63 | 7206.25 | 19621.50 | 29.13 | 595.81 | 198.34 | 14630.37 |
| 1.84 | 65.20 | 54717.27 | 340.49 | 10533.24 | 0.46 | 4.37 | 19.95 | 5.20 | 10465.06 | 25108.28 | 37.91 | 1755.33 | 279.77 | 17922.36 |
| 2.00 | 59.98 | 54460.30 | 345.37 | 9654.86 | 0.41 | 3.50 | 15.54 | 3.88 | 7628.81 | 26219.05 | 40.46 | 1289.95 | 192.67 | 17723.61 |
| 2.14 | 36.94 | 45647.79 | 267.41 | 8183.27 | 0.35 | 2.45 | 13.28 | 3.89 | 6990.47 | 23801.90 | 37.47 | 819.90 | 195.42 | 15838.87 |
| 2.28 | 46.59 | 52446.09 | 359.76 | 9183.01 | 0.51 | 3.24 | 15.37 | 6.95 | 8159.14 | 26727.54 | 39.77 | 1281.76 | 229.62 | 16811.94 |
| 2.43 | 62.54 | 49062.76 | 287.73 | 9441.97 | 0.47 | 3.93 | 18.55 | 4.37 | 9275.33 | 24435.15 | 38.77 | 924.17 | 267.46 | 16886.00 |
| 2.79 | 58.09 | 56570.85 | 407.47 | 9607.94 | 0.51 | 3.45 | 15.83 | 6.61 | 7911.30 | 27720.46 | 43.67 | 1369.49 | 206.33 | 17691.97 |
| 2.88 | 72.93 | 49036.07 | 315.32 | 9096.70 | 0.78 | 4.51 | 20.69 | 4.48 | 10426.06 | 24426.37 | 40.55 | 1464.01 | 279.30 | 16170.32 |
| 2.98 | 36.75 | 40445.41 | 255.21 | 7419.68 | 0.45 | 2.44 | 11.70 | 3.85 | 6453.84 | 19582.08 | 39.29 | 919.60 | 167.13 | 13281.52 |
| 3.17 | 53.79 | 50082.88 | 352.02 | 8563.38 | 0.53 | 3.46 | 17.44 | 5.47 | 8349.68 | 24952.13 | 42.44 | 1496.02 | 211.13 | 14749.82 |
| 3.36 | 87.02 | 44383.33 | 276.30 | 9494.31 | 1.19 | 5.80 | 25.18 | 4.35 | 11454.26 | 17823.79 | 39.07 | 1824.89 | 279.15 | 13597.28 |
| 3.51 | 122.86 | 49908.66 | 354.30 | 10331.62 | 1.68 | 7.55 | 39.06 | 9.90 | 15963.96 | 21398.92 | 39.72 | 2780.62 | 367.35 | 14319.99 |
| 3.61 | 148.87 | 42548.42 | 327.47 | 9347.92 | 3.46 | 9.78 | 46.67 | 4.99 | 17988.48 | 20990.46 | 35.37 | 2175.73 | 403.17 | 12038.71 |
| 3.72 | 132.56 | 43299.93 | 308.03 | 9417.64 | 1.94 | 7.59 | 34.80 | 4.33 | 14751.01 | 20993.75 | 38.85 | 2282.93 | 352.65 | 13240.99 |
| 3.80 | 144.85 | 45983.75 | 312.02 | 11069.07 | 2.90 | 8.44 | 46.22 | 7.00 | 16629.37 | 21527.45 | 38.31 | 2220.47 | 377.74 | 13533.33 |
| 3.85 | 184.98 | 51139.59 | 362.66 | 12587.34 | 3.07 | 10.34 | 53.54 | 9.02 | 20880.26 | 24182.16 | 52.36 | 3387.57 | 517.86 | 16236.84 |
| 3.91 | 91.82 | 52100.58 | 337.91 | 11112.87 | 1.24 | 5.30 | 28.24 | 5.27 | 12130.22 | 23741.61 | 52.14 | 2169.66 | 352.66 | 15973.70 |
| 3.96 | 167.79 | 47171.48 | 373.33 | 15381.41 | 3.92 | 10.12 | 50.19 | 9.60 | 18800.40 | 22050.93 | 43.99 | 3498.62 | 446.94 | 13841.42 |
| 4.02 | 169.28 | 49633.79 | 371.72 | 13821.49 | 3.59 | 9.99 | 52.97 | 9.80 | 18981.21 | 20829.43 | 48.79 | 4700.84 | 428.74 | 13909.44 |
| 4.07 | 202.56 | 58280.45 | 452.13 | 16093.30 | 5.40 | 12.17 | 61.50 | 15.62 | 22231.29 | 24610.81 | 55.92 | 5217.54 | 491.69 | 15051.32 |
| 4.12 | 198.40 | 50781.58 | 402.62 | 14279.95 | 6.69 | 11.59 | 59.85 | 13.44 | 21492.22 | 21980.21 | 52.51 | 5034.88 | 462.33 | 14597.52 |
| 4.17 | 131.08 | 44719.91 | 341.14 | 11797.19 | 3.73 | 7.95 | 41.63 | 8.80 | 14311.25 | 20137.27 | 49.71 | 3583.76 | 311.59 | 12861.79 |
| 4.22 | 122.65 | 49802.12 | 347.11 | 12811.93 | 1.30 | 7.44 | 34.40 | 5.56 | 15471.50 | 21926.96 | 49.73 | 3187.69 | 433.13 | 14295.13 |
| 4.27 | 51.11 | 39207.75 | 276.16 | 9224.46 | 0.83 | 3.71 | 18.96 | 5.46 | 8924.84 | 18529.59 | 46.13 | 1729.98 | 244.98 | 11518.11 |
| 4.32 | 114.18 | 48667.09 | 337.28 | 12268.08 | 1.51 | 7.17 | 33.70 | 5.86 | 15285.80 | 20968.17 | 47.90 | 3164.20 | 423.43 | 13809.05 |
| 4.37 | 128.18 | 43686.41 | 349.20 | 12769.33 | 2.64 | 7.95 | 40.27 | 6.84 | 14868.94 | 21834.61 | 51.33 | 2828.06 | 330.98 | 13147.97 |
| 4.42 | 117.34 | 42408.29 | 331.56 | 12838.56 | 2.75 | 7.52 | 38.41 | 11.33 | 14343.50 | 33592.31 | 52.98 | 3431.73 | 322.55 | 12074.64 |
| 4.47 | 181.83 | 48808.34 | 397.25 | 12825.52 | 4.01 | 11.36 | 55.11 | 14.93 | 20103.53 | 36181.62 | 58.59 | 4775.91 | 432.57 | 12469.69 |
| 4.52 | 187.60 | 46852.28 | 396.95 | 12377.16 | 4.02 | 11.73 | 55.78 | 15.66 | 20677.20 | 37065.95 | 58.06 | 4900.04 | 447.14 | 13312.64 |
| 4.57 | 133.83 | 40765.47 | 354.45 | 10332.93 | 2.62 | 8.77 | 41.99 | 8.87 | 16401.94 | 35776.17 | 51.18 | 2523.18 | 359.91 | 12734.23 |
| 4.62 | 177.24 | 47367.31 | 396.02 | 11974.64 | 2.31 | 10.60 | 52.07 | 9.27 | 19239.61 | 36068.03 | 57.40 | 4717.83 | 444.30 | 13192.91 |
| 4.67 | 159.97 | 43716.37 | 346.47 | 14423.00 | 2.03 | 9.56 | 45.01 | 10.44 | 17482.23 | 33488.04 | 50.63 | 4000.76 | 418.21 | 12084.41 |
| 4.72 | 124.54 | 36390.42 | 293.87 | 10795.73 | 1.95 | 7.96 | 36.93 | 9.30 | 15000.71 | 29315.32 | 50.12 | 2914.94 | 361.72 | 10882.59 |
| 4.76 | 91.72 | 37512.84 | 284.73 | 9761.83 | 1.27 | 5.52 | 26.40 | 7.04 | 11236.54 | 31244.15 | 50.62 | 2251.52 | 291.73 | 10653.47 |
| 4.80 | 175.37 | 46154.54 | 388.81 | 12113.96 | 2.98 | 10.23 | 54.93 | 12.70 | 18648.29 | 37047.51 | 63.12 | 4238.65 | 392.14 | 12611.60 |
| 4.84 | 179.28 | 51040.68 | 410.18 | 12553.38 | 2.72 | 10.53 | 56.02 | 12.28 | 19924.46 | 38129.08 | 67.23 | 4605.13 | 454.81 | 13223.30 |
| 4.87 | 201.42 | 52431.82 | 431.65 | 13250.89 | 2.56 | 11.54 | 57.90 | 14.70 | 20590.08 | 39915.19 | 68.15 | 5102.34 | 457.33 | 13801.21 |
| 4.90 | 129.80 | 36935.14 | 282.22 | 8781.56 | 2.06 | 8.25 | 43.57 | 9.14 | 17143.10 | 33133.72 | 53.16 | 1681.68 | 415.84 | 10756.04 |
| 4.94 | 141.10 | 48489.08 | 319.41 | 10781.88 | 1.75 | 8.63 | 44.86 | 10.45 | 19650.97 | 35410.89 | 66.89 | 3510.32 | 601.47 | 14293.65 |
| 4.97 | 203.50 | 50066.59 | 395.43 | 11681.02 | 2.61 | 11.75 | 62.21 | 14.57 | 21462.88 | 36832.29 | 70.56 | 5168.81 | 507.07 | 13283.94 |
| 5.00 | 174.21 | 51892.01 | 371.26 | 11343.86 | 1.97 | 9.96 | 54.72 | 12.23 | 21887.72 | 39999.61 | 72.78 | 3674.29 | 619.82 | 14233.94 |
| 5.03 | 173.35 | 51609.77 | 367.45 | 10876.73 | 1.76 | 8.90 | 51.71 | 10.75 | 18897.46 | 37439.07 | 73.93 | 3697.96 | 534.13 | 13621.39 |
| 5.09 | 146.23 | 48567.52 | 350.65 | 10915.58 | 1.78 | 8.59 | 45.54 | 11.47 | 18074.44 | 36813.10 | 72.02 | 3935.10 | 501.80 | 13248.66 |
| 5.12 | 141.35 | 50651.17 | 371.22 | 10610.56 | 1.92 | 7.89 | 43.35 | 10.57 | 15793.82 | 40130.00 | 75.99 | 3382.17 | 383.67 | 14355.25 |
| 5.16 | 161.35 | 38331.90 | 287.28 | 8768.34 | 2.05 | 8.92 | 48.38 | 9.20 | 16649.99 | 35925.76 | 61.47 | 1899.02 | 357.78 | 11080.96 |
| 5.19 | 148.90 | 42184.80 | 337.52 | 10207.10 | 1.97 | 8.94 | 49.44 | 12.09 | 17271.89 | 35754.49 | 72.95 | 3329.79 | 400.44 | 12173.99 |
| 5.22 | 182.40 | 50273.94 | 386.72 | 12014.48 | 2.20 | 10.37 | 55.05 | 13.57 | 19351.91 | 39127.19 | 75.15 | 4239.28 | 475.64 | 13007.80 |
| 5.25 | 158.07 | 45802.80 | 352.37 | 10628.56 | 2.22 | 9.16 | 48.77 | 12.64 | 17469.80 | 35607.65 | 75.15 | 3826.47 | 412.29 | 12266.33 |
| 5.31 | 138.01 | 46984.89 | 353.94 | 11824.26 | 2.16 | 8.87 | 46.59 | 13.03 | 17513.33 | 37900.03 | 73.24 | 3733.32 | 445.05 | 12761.11 |
| 5.34 | 130.95 | 40219.08 | 352.60 | 11038.00 | 2.73 | 8.35 | 46.10 | 13.01 | 16085.73 | 36680.36 | 72.21 | 2718.79 | 371.12 | 12252.73 |
| 5.38 | 152.63 | 34876.64 | 342.93 | 10622.82 | 2.77 | 9.75 | 48.15 | 14.28 | 16637.42 | 34864.06 | 73.05 | 2220.46 | 447.62 | 12236.18 |
| 5.40 | 143.85 | 30012.60 | 248.10 | 8300.23 | 3.23 | 9.89 | 48.67 | 12.73 | 16731.96 | 32579.10 | 54.59 | 963.78 | 354.83 | 9431.36 |
| 5.43 | 187.17 | 41721.78 | 330.65 | 10070.41 | 3.57 | 11.44 | 53.53 | 14.75 | 17974.56 | 35937.41 | 71.05 | 3088.46 | 405.79 | 11963.72 |
| 5.45 | 157.01 | 42831.96 | 352.08 | 10037.67 | 3.93 | 10.01 | 51.50 | 13.84 | 17628.95 | 34830.81 | 74.88 | 3928.51 | 375.99 | 11358.74 |
| 5.47 | 143.33 | 42751.16 | 340.13 | 9453.81 | 2.73 | 9.36 | 48.85 | 12.39 | 17297.57 | 34175.61 | 73.03 | 3688.45 | 385.71 | 11100.66 |
| 5.49 | 199.28 | 44028.76 | 381.09 | 9398.89 | 2.85 | 11.46 | 55.14 | 13.54 | 19112.58 | 36384.40 | 75.19 | 3850.18 | 391.74 | 11244.27 |
| 5.52 | 175.31 | 45068.80 | 355.00 | 10246.83 | 3.35 | 10.33 | 54.78 | 13.15 | 18501.79 | 36803.03 | 72.99 | 3807.79 | 395.14 | 11119.47 |
| 5.54 | 152.62 | 38231.80 | 303.89 | 8638.85 | 3.02 | 9.87 | 50.99 | 11.19 | 16434.49 | 35253.35 | 67.20 | 2820.01 | 354.54 | 11460.28 |
| 5.56 | 154.12 | 31914.98 | 217.93 | 7780.10 | 3.44 | 10.01 | 48.99 | 10.42 | 16945.83 | 32924.82 | 54.09 | 1273.83 | 352.66 | 8803.48 |
| 5.58 | 194.59 | 39632.05 | 304.12 | 9109.75 | 3.85 | 11.98 | 60.69 | 14.64 | 19767.44 | 35648.70 | 68.99 | 3274.66 | 426.93 | 10465.70 |
| 5.60 | 196.70 | 34828.78 | 290.20 | 7941.70 | 3.55 | 12.33 | 65.50 | 14.70 | 19666.92 | 34623.66 | 62.63 | 2637.04 | 370.45 | 9566.28 |
| 5.61 | 148.14 | 32798.63 | 281.37 | 8331.34 | 3.00 | 9.40 | 49.64 | 14.09 | 15350.79 | 27944.08 | 60.03 | 3529.30 | 291.70 | 8827.90 |
| 5.63 | 177.94 | 36139.67 | 315.55 | 8984.41 | 3.41 | 11.33 | 57.78 | 14.35 | 18068.65 | 33275.30 | 68.87 | 3442.84 | 360.80 | 9969.31 |
| 5.65 | 188.59 | 36399.87 | 332.31 | 8733.96 | 4.01 | 11.89 | 59.47 | 15.38 | 18388.85 | 33018.08 | 71.76 | 3970.22 | 350.93 | 9690.97 |
| 5.67 | 187.55 | 34459.96 | 294.01 | 8041.48 | 4.23 | 12.40 | 65.00 | 16.51 | 19034.30 | 33537.53 | 66.98 | 2999.67 | 373.21 | 8905.15 |
| 5.69 | 143.30 | 40150.28 | 329.59 | 8478.15 | 2.89 | 9.65 | 51.75 | 17.16 | 17729.12 | 34957.98 | 75.39 | 3723.33 | 414.32 | 10302.17 |
| 5.70 | 165.32 | 31686.44 | 239.54 | 7624.27 | 3.18 | 10.54 | 52.93 | 11.89 | 18790.30 | 29503.07 | 62.17 | 2693.58 | 454.81 | 8595.23 |
| 5.72 | 159.44 | 34328.60 | 299.43 | 8225.74 | 3.32 | 10.51 | 53.29 | 13.61 | 17071.49 | 30422.95 | 69.97 | 3090.84 | 351.43 | 9152.78 |
| 5.74 | 200.39 | 37347.55 | 321.50 | 10240.19 | 3.84 | 12.55 | 64.70 | 14.92 | 19468.81 | 34589.75 | 72.71 | 4095.06 | 347.90 | 10022.67 |
| 5.76 | 168.69 | 36410.77 | 322.25 | 8990.31 | 3.66 | 10.64 | 55.38 | 13.65 | 17303.96 | 31670.11 | 76.64 | 4317.96 | 324.24 | 9476.68 |
| 5.78 | 184.85 | 43467.50 | 368.14 | 11248.21 | 4.27 | 11.18 | 59.77 | 15.76 | 19916.87 | 36063.33 | 81.68 | 4910.07 | 389.15 | 10955.54 |
| 5.79 | 143.23 | 37171.96 | 320.65 | 9083.38 | 2.59 | 9.62 | 47.28 | 11.83 | 16134.36 | 32488.93 | 75.42 | 3748.23 | 345.13 | 10243.78 |
| 5.81 | 163.24 | 39260.78 | 359.16 | 9333.42 | 4.48 | 10.39 | 53.68 | 14.35 | 17216.24 | 33278.19 | 78.91 | 4637.52 | 318.75 | 10302.15 |
| 5.83 | 172.03 | 39530.37 | 334.97 | 9516.06 | 3.99 | 10.43 | 52.38 | 13.79 | 17950.22 | 34302.52 | 79.97 | 4282.57 | 348.99 | 11449.94 |
| 5.84 | 142.99 | 29731.44 | 218.74 | 7133.32 | 2.52 | 8.98 | 44.24 | 9.51 | 15383.31 | 30859.49 | 66.66 | 1430.12 | 304.49 | 9023.21 |
| 5.86 | 144.40 | 30977.77 | 275.45 | 7228.96 | 3.89 | 9.43 | 48.33 | 13.00 | 16152.62 | 30370.99 | 68.72 | 2838.31 | 328.42 | 8604.46 |
| 5.90 | 138.07 | 31476.57 | 282.65 | 7408.44 | 2.35 | 8.82 | 46.60 | 12.97 | 14420.34 | 28100.30 | 72.04 | 3632.40 | 270.71 | 8511.00 |
| 5.91 | 172.72 | 37595.02 | 338.59 | 9133.82 | 2.95 | 10.56 | 55.63 | 14.65 | 17362.84 | 32492.41 | 80.75 | 4720.97 | 321.57 | 10041.45 |
| 5.93 | 152.90 | 38764.37 | 339.26 | 9477.79 | 2.77 | 9.46 | 49.87 | 12.17 | 16174.19 | 32728.80 | 78.71 | 4058.46 | 323.93 | 10051.18 |
| 5.95 | 189.34 | 38885.10 | 354.27 | 9511.30 | 3.21 | 11.48 | 60.70 | 15.33 | 18810.07 | 34750.03 | 83.25 | 4521.13 | 356.19 | 10568.16 |
| 5.97 | 158.51 | 35856.81 | 326.51 | 8543.00 | 5.03 | 10.06 | 55.96 | 16.34 | 17494.08 | 33295.54 | 83.12 | 4054.80 | 320.13 | 10139.45 |
| 5.98 | 145.36 | 29363.76 | 240.39 | 7413.98 | 5.68 | 9.53 | 50.85 | 15.66 | 16219.68 | 30703.72 | 64.08 | 2185.75 | 296.70 | 8273.55 |
| 6.00 | 10.21 | 23147.11 | 211.63 | 8620.90 | 4.92 | 6.23 | 11.01 | 17.74 | 1693.05 | 31881.46 | 61.83 | 1390.10 | 330.71 | 8649.50 |
| 6.02 | 140.96 | 39860.04 | 330.01 | 9518.45 | 3.06 | 8.63 | 45.20 | 12.34 | 16317.45 | 35774.75 | 81.33 | 4161.79 | 345.59 | 11537.32 |
| 6.04 | 26.97 | 33639.90 | 260.53 | 9256.54 | 3.51 | 7.55 | 25.70 | 17.16 | 6187.01 | 34977.95 | 77.38 | 3279.77 | 335.52 | 9492.07 |
| 6.06 | 12.75 | 24347.12 | 190.47 | 7538.58 | 3.45 | 7.30 | 15.62 | 16.42 | 3035.61 | 31082.71 | 64.37 | 1841.18 | 314.70 | 8065.81 |
| 6.07 | 7.17 | 15569.95 | 134.31 | 8381.85 | 4.01 | 5.26 | 4.80 | 11.94 | 208.18 | 27714.05 | 50.86 | 743.80 | 247.33 | 7197.86 |
| 6.09 | 10.94 | 27536.27 | 199.99 | 9933.45 | 2.07 | 4.77 | 8.06 | 12.88 | 1191.91 | 30134.42 | 70.69 | 2523.06 | 276.27 | 8799.06 |
| 6.11 | 89.02 | 33316.69 | 304.06 | 9563.67 | 2.65 | 7.02 | 31.34 | 12.26 | 11035.11 | 31341.19 | 72.90 | 3585.25 | 277.54 | 9270.56 |
| 6.18 | 153.04 | 34727.75 | 308.82 | 8606.13 | 2.71 | 9.11 | 49.23 | 12.89 | 17648.63 | 31592.67 | 78.32 | 3693.97 | 365.84 | 9482.67 |
| 6.20 | 10.03 | 27359.25 | 178.89 | 9812.04 | 2.49 | 5.13 | 11.12 | 13.36 | 1947.92 | 31609.89 | 64.13 | 1811.55 | 301.81 | 8502.75 |
| 6.22 | 11.73 | 26503.74 | 200.44 | 7766.24 | 3.89 | 5.48 | 13.33 | 15.45 | 2615.49 | 31725.24 | 76.25 | 2257.44 | 272.07 | 8644.13 |
| 6.25 | 137.55 | 32228.48 | 294.69 | 8135.49 | 4.74 | 8.50 | 48.24 | 14.36 | 15674.41 | 29177.05 | 79.30 | 4092.08 | 282.74 | 9112.82 |
| 6.27 | 10.15 | 22502.31 | 158.55 | 6916.74 | 3.47 | 5.21 | 16.77 | 14.80 | 3318.70 | 27555.51 | 65.48 | 1924.17 | 315.87 | 7113.69 |
| 6.29 | 117.69 | 23199.08 | 205.07 | 6109.83 | 4.87 | 7.71 | 43.14 | 14.38 | 14118.29 | 23926.77 | 62.73 | 2357.73 | 240.32 | 6939.79 |
| 6.32 | 19.07 | 26090.89 | 120.99 | 7801.97 | 4.14 | 6.69 | 21.70 | 15.73 | 5067.20 | 31456.35 | 69.52 | 1844.77 | 339.79 | 9022.52 |
| 6.37 | 12.19 | 21832.93 | 114.14 | 7310.09 | 3.51 | 5.43 | 12.98 | 14.74 | 2844.61 | 29512.23 | 64.45 | 1447.04 | 277.24 | 7517.95 |

Extended Data Table 2 (Contd).: Metal concentration and weathering indices data for SL1 sediment core.

| **Age (cal ka BP)** | **Ni (ppm)** | **Pb (ppm)** | **Sr (ppm)** | **Zn (ppm)** | **Zr (ppm)** | **Rb (ppm)** | **V (ppm)** | **Ti (ppm)** | **K/Al** | **CIA** | **PIA** | **ICV** | **100Ti/Al** |
| --- | --- | --- | --- | --- | --- | --- | --- | --- | --- | --- | --- | --- | --- |
| 0.25 | 40.58 | 12.21 | 76.21 | 114.37 | 48.00 | 168.43 | 38.68 | 1489.15 | 0.35 | 54.32 | 55.83 | 1.50 | 2.64 |
| 0.54 | 44.71 | 17.13 | 84.75 | 146.46 | 36.10 | 181.94 | 37.50 | 1433.71 | 0.42 | 52.10 | 52.99 | 1.46 | 2.18 |
| 1.31 | 27.43 | 22.89 | 59.82 | 46.58 | 49.61 | 105.82 | 29.51 | 1427.79 | 0.50 | 49.75 | 49.61 | 1.36 | 2.43 |
| 1.48 | 11.77 | 29.19 | 80.86 | 290.81 | 33.66 | 271.50 | 23.11 | 1039.54 | 0.45 | 51.64 | 52.41 | 1.18 | 1.16 |
| 1.65 | 6.41 | 13.67 | 50.83 | 146.40 | 18.07 | 130.09 | 8.85 | 508.85 | 0.34 | 57.97 | 60.94 | 1.00 | 0.94 |
| 1.75 | 2.79 | 28.81 | 49.76 | 240.00 | 25.45 | 145.80 | 11.07 | 683.52 | 0.54 | 48.12 | 47.08 | 1.27 | 1.05 |
| 1.84 | 4.13 | 32.89 | 78.20 | 276.90 | 42.44 | 285.14 | 15.38 | 873.16 | 0.46 | 51.57 | 52.33 | 1.15 | 0.90 |
| 2.00 | 2.70 | 35.52 | 75.07 | 138.94 | 31.21 | 265.79 | 15.32 | 809.09 | 0.48 | 51.77 | 52.69 | 1.09 | 0.84 |
| 2.14 | 2.23 | 33.68 | 61.63 | 150.56 | 32.54 | 204.42 | 8.47 | 538.37 | 0.52 | 50.42 | 50.66 | 1.14 | 0.67 |
| 2.28 | 2.36 | 36.00 | 74.90 | 648.55 | 31.47 | 307.13 | 11.97 | 668.91 | 0.51 | 51.50 | 52.35 | 1.11 | 0.72 |
| 2.43 | 2.58 | 37.64 | 65.96 | 193.46 | 30.06 | 181.23 | 14.72 | 816.50 | 0.50 | 50.42 | 50.64 | 1.16 | 0.94 |
| 2.79 | 2.86 | 37.87 | 85.37 | 508.58 | 33.93 | 267.85 | 20.57 | 943.31 | 0.49 | 52.26 | 53.49 | 1.08 | 0.94 |
| 2.88 | 2.72 | 33.69 | 73.16 | 439.54 | 32.01 | 334.71 | 12.33 | 755.27 | 0.50 | 51.10 | 51.69 | 1.17 | 0.87 |
| 2.98 | 1.69 | 31.84 | 56.87 | 159.11 | 26.48 | 214.97 | 9.39 | 507.80 | 0.48 | 51.60 | 52.44 | 1.11 | 0.71 |
| 3.17 | 2.77 | 32.98 | 73.20 | 524.17 | 31.78 | 255.57 | 15.70 | 715.07 | 0.50 | 52.72 | 54.27 | 1.09 | 0.81 |
| 3.36 | 4.31 | 21.26 | 65.46 | 239.77 | 34.39 | 227.45 | 22.28 | 1102.35 | 0.40 | 52.66 | 53.76 | 1.17 | 1.40 |
| 3.51 | 4.97 | 20.95 | 82.42 | 454.59 | 29.39 | 248.43 | 29.88 | 1407.48 | 0.43 | 52.96 | 54.31 | 1.23 | 1.59 |
| 3.61 | 8.76 | 22.01 | 70.71 | 221.68 | 40.42 | 175.70 | 47.66 | 1740.67 | 0.49 | 51.51 | 52.33 | 1.34 | 2.31 |
| 3.72 | 5.56 | 23.09 | 71.02 | 363.60 | 39.49 | 216.10 | 31.55 | 1502.96 | 0.48 | 51.03 | 51.56 | 1.32 | 1.96 |
| 3.80 | 7.08 | 23.49 | 80.78 | 256.91 | 49.80 | 230.88 | 44.62 | 1687.02 | 0.47 | 50.82 | 51.23 | 1.32 | 2.07 |
| 3.85 | 8.04 | 28.47 | 80.95 | 252.99 | 61.23 | 255.28 | 45.01 | 1992.37 | 0.47 | 49.80 | 49.71 | 1.43 | 2.20 |
| 3.91 | 3.50 | 26.90 | 77.28 | 222.93 | 33.13 | 319.66 | 20.12 | 1130.54 | 0.46 | 51.56 | 52.31 | 1.20 | 1.23 |
| 3.96 | 8.75 | 23.47 | 92.18 | 265.79 | 46.28 | 243.71 | 53.25 | 1838.00 | 0.47 | 49.72 | 49.59 | 1.52 | 2.20 |
| 4.02 | 9.52 | 18.63 | 87.14 | 302.00 | 52.60 | 277.58 | 51.30 | 1912.56 | 0.42 | 51.35 | 51.92 | 1.44 | 2.18 |
| 4.07 | 12.66 | 23.85 | 95.30 | 436.10 | 55.37 | 291.09 | 65.37 | 2202.01 | 0.42 | 52.70 | 53.90 | 1.40 | 2.13 |
| 4.12 | 12.44 | 20.68 | 93.69 | 292.12 | 60.13 | 280.33 | 62.15 | 2194.64 | 0.43 | 50.67 | 50.97 | 1.50 | 2.44 |
| 4.17 | 8.11 | 18.67 | 78.93 | 185.86 | 44.52 | 256.95 | 42.93 | 1587.80 | 0.45 | 50.56 | 50.82 | 1.38 | 2.01 |
| 4.22 | 4.43 | 21.13 | 85.06 | 216.81 | 30.49 | 266.14 | 29.22 | 1501.17 | 0.44 | 50.92 | 51.33 | 1.32 | 1.70 |
| 4.27 | 2.71 | 18.15 | 63.91 | 153.45 | 24.53 | 239.51 | 12.02 | 701.75 | 0.47 | 51.06 | 51.59 | 1.22 | 1.01 |
| 4.32 | 4.22 | 19.70 | 81.92 | 182.61 | 31.29 | 286.65 | 28.25 | 1331.58 | 0.43 | 51.38 | 51.99 | 1.31 | 1.55 |
| 4.37 | 7.15 | 20.53 | 76.74 | 403.95 | 38.27 | 250.73 | 41.46 | 1608.47 | 0.50 | 48.75 | 48.12 | 1.45 | 2.08 |
| 4.42 | 8.66 | 15.52 | 74.74 | 383.70 | 43.58 | 259.27 | 40.79 | 1428.12 | 0.79 | 45.15 | 40.42 | 1.68 | 1.90 |
| 4.47 | 13.49 | 15.63 | 79.49 | 219.78 | 59.07 | 270.02 | 60.44 | 2034.85 | 0.74 | 47.37 | 44.89 | 1.64 | 2.36 |
| 4.52 | 13.12 | 16.46 | 84.67 | 286.46 | 60.35 | 274.03 | 64.68 | 2253.94 | 0.79 | 45.24 | 40.59 | 1.74 | 2.72 |
| 4.57 | 8.99 | 17.48 | 74.00 | 145.00 | 39.80 | 206.15 | 43.18 | 1737.80 | 0.88 | 43.76 | 36.72 | 1.70 | 2.41 |
| 4.62 | 10.62 | 14.17 | 86.04 | 134.72 | 56.56 | 290.41 | 54.84 | 2143.29 | 0.76 | 46.12 | 42.46 | 1.67 | 2.56 |
| 4.67 | 8.79 | 14.37 | 80.86 | 407.69 | 49.35 | 283.50 | 48.59 | 1912.42 | 0.77 | 45.93 | 42.08 | 1.75 | 2.47 |
| 4.72 | 7.36 | 13.41 | 67.18 | 186.00 | 42.04 | 242.06 | 34.98 | 1577.60 | 0.81 | 44.29 | 38.75 | 1.74 | 2.45 |
| 4.76 | 4.24 | 14.64 | 65.51 | 162.46 | 28.68 | 218.34 | 27.40 | 1254.89 | 0.83 | 44.89 | 39.44 | 1.58 | 1.89 |
| 4.80 | 12.38 | 16.91 | 82.26 | 277.46 | 56.86 | 275.32 | 56.76 | 2123.71 | 0.80 | 45.55 | 41.02 | 1.69 | 2.60 |
| 4.84 | 11.75 | 16.69 | 84.17 | 224.51 | 66.32 | 300.06 | 55.70 | 2133.64 | 0.75 | 47.09 | 44.34 | 1.60 | 2.36 |
| 4.87 | 12.59 | 17.87 | 90.25 | 259.08 | 65.57 | 335.37 | 66.17 | 2409.74 | 0.76 | 46.66 | 43.45 | 1.64 | 2.60 |
| 4.90 | 8.87 | 19.14 | 59.18 | 105.85 | 39.67 | 181.96 | 46.38 | 1635.82 | 0.90 | 44.45 | 37.65 | 1.67 | 2.50 |
| 4.94 | 8.07 | 19.41 | 73.39 | 217.24 | 61.84 | 287.60 | 46.32 | 1946.18 | 0.73 | 47.05 | 44.38 | 1.56 | 2.27 |
| 4.97 | 12.69 | 14.74 | 83.39 | 329.56 | 61.98 | 321.00 | 45.80 | 1940.67 | 0.74 | 47.39 | 44.97 | 1.62 | 2.19 |
| 5.00 | 9.34 | 20.21 | 79.66 | 270.53 | 72.32 | 294.75 | 65.55 | 2570.66 | 0.77 | 47.04 | 44.07 | 1.58 | 2.80 |
| 5.03 | 8.47 | 17.78 | 76.70 | 381.59 | 43.19 | 274.63 | 45.79 | 1982.27 | 0.73 | 48.27 | 46.65 | 1.48 | 2.17 |
| 5.09 | 11.06 | 15.88 | 77.47 | 135.27 | 49.94 | 279.16 | 45.06 | 1872.66 | 0.76 | 47.12 | 44.32 | 1.56 | 2.18 |
| 5.12 | 9.33 | 20.51 | 79.10 | 161.36 | 46.39 | 278.88 | 43.69 | 1775.56 | 0.79 | 46.76 | 43.36 | 1.51 | 1.98 |
| 5.16 | 10.50 | 17.82 | 62.48 | 196.52 | 50.26 | 162.73 | 52.30 | 2029.25 | 0.94 | 44.17 | 36.40 | 1.68 | 2.99 |
| 5.19 | 9.81 | 16.36 | 69.28 | 245.31 | 44.52 | 166.18 | 48.60 | 1875.02 | 0.85 | 45.00 | 39.45 | 1.68 | 2.51 |
| 5.22 | 10.15 | 16.31 | 80.04 | 268.55 | 55.85 | 268.30 | 57.70 | 2200.01 | 0.78 | 46.74 | 43.45 | 1.59 | 2.47 |
| 5.25 | 9.83 | 14.23 | 73.13 | 237.51 | 47.51 | 231.33 | 50.84 | 2012.16 | 0.78 | 46.77 | 43.52 | 1.59 | 2.48 |
| 5.31 | 8.61 | 16.79 | 76.20 | 281.75 | 46.91 | 251.27 | 44.31 | 1870.72 | 0.81 | 45.67 | 41.19 | 1.62 | 2.25 |
| 5.34 | 10.15 | 19.11 | 71.93 | 290.92 | 46.67 | 193.80 | 50.84 | 1796.98 | 0.91 | 42.96 | 34.67 | 1.76 | 2.52 |
| 5.38 | 11.92 | 20.66 | 67.61 | 326.23 | 61.74 | 135.57 | 51.74 | 1884.98 | 1.00 | 40.34 | 28.22 | 1.96 | 3.05 |
| 5.40 | 12.16 | 19.24 | 53.47 | 154.78 | 48.32 | 133.84 | 58.42 | 1903.34 | 1.09 | 40.78 | 26.31 | 1.91 | 3.58 |
| 5.43 | 13.07 | 20.46 | 65.68 | 97.96 | 62.15 | 161.36 | 62.15 | 2347.37 | 0.86 | 44.88 | 39.02 | 1.70 | 3.18 |
| 5.45 | 11.70 | 13.13 | 70.20 | 159.56 | 48.67 | 239.68 | 58.67 | 2078.66 | 0.81 | 46.27 | 42.25 | 1.65 | 2.74 |
| 5.47 | 11.88 | 12.25 | 67.54 | 85.92 | 50.92 | 275.55 | 51.70 | 1939.48 | 0.80 | 47.01 | 43.79 | 1.60 | 2.56 |
| 5.49 | 12.08 | 13.00 | 71.12 | 80.11 | 53.18 | 207.53 | 64.11 | 2611.77 | 0.83 | 46.92 | 43.37 | 1.64 | 3.35 |
| 5.52 | 12.48 | 12.41 | 71.20 | 91.06 | 56.64 | 180.75 | 55.66 | 2191.92 | 0.82 | 46.87 | 43.36 | 1.61 | 2.75 |
| 5.54 | 11.53 | 14.79 | 65.64 | 70.71 | 60.18 | 197.13 | 54.61 | 2045.93 | 0.92 | 44.20 | 36.75 | 1.73 | 3.02 |
| 5.56 | 11.45 | 15.42 | 47.50 | 65.84 | 50.55 | 114.98 | 55.46 | 2033.30 | 1.03 | 42.96 | 31.87 | 1.79 | 3.60 |
| 5.58 | 15.06 | 14.08 | 62.32 | 85.30 | 65.28 | 185.58 | 72.14 | 2542.01 | 0.90 | 45.23 | 39.11 | 1.75 | 3.62 |
| 5.60 | 15.77 | 15.48 | 55.78 | 102.58 | 61.21 | 177.76 | 79.52 | 2582.22 | 0.99 | 43.81 | 34.49 | 1.85 | 4.19 |
| 5.61 | 12.44 | 9.54 | 62.51 | 213.49 | 45.59 | 186.51 | 59.65 | 1940.92 | 0.85 | 45.23 | 39.82 | 1.79 | 3.34 |
| 5.63 | 14.72 | 12.62 | 62.05 | 91.35 | 56.23 | 184.89 | 73.24 | 2347.25 | 0.92 | 44.21 | 36.78 | 1.83 | 3.67 |
| 5.65 | 16.30 | 11.30 | 62.95 | 77.03 | 56.20 | 223.83 | 77.50 | 2437.73 | 0.91 | 44.84 | 38.25 | 1.83 | 3.78 |
| 5.67 | 17.80 | 15.12 | 55.64 | 99.02 | 57.57 | 181.76 | 76.96 | 2350.47 | 0.97 | 44.33 | 35.99 | 1.84 | 3.85 |
| 5.69 | 11.45 | 12.40 | 63.59 | 65.14 | 54.79 | 238.09 | 56.36 | 1951.35 | 0.87 | 46.35 | 41.75 | 1.67 | 2.75 |
| 5.70 | 10.95 | 10.70 | 50.41 | 57.32 | 52.30 | 154.53 | 65.21 | 2166.91 | 0.93 | 44.47 | 37.10 | 1.85 | 3.86 |
| 5.72 | 13.04 | 12.16 | 57.94 | 164.24 | 53.06 | 237.71 | 67.48 | 2153.57 | 0.89 | 45.17 | 39.22 | 1.77 | 3.54 |
| 5.74 | 16.95 | 12.83 | 69.42 | 216.77 | 68.08 | 223.34 | 84.57 | 2652.28 | 0.93 | 44.07 | 36.43 | 1.91 | 4.01 |
| 5.76 | 14.33 | 10.76 | 62.23 | 144.89 | 57.35 | 254.93 | 73.74 | 2284.62 | 0.87 | 45.32 | 39.73 | 1.81 | 3.54 |
| 5.78 | 14.49 | 11.01 | 73.94 | 186.23 | 58.92 | 296.71 | 72.05 | 2348.40 | 0.83 | 46.21 | 41.95 | 1.76 | 3.05 |
| 5.79 | 10.76 | 12.51 | 64.42 | 153.28 | 41.22 | 277.73 | 60.14 | 1972.84 | 0.87 | 44.95 | 38.97 | 1.76 | 3.00 |
| 5.81 | 14.87 | 9.96 | 70.12 | 149.78 | 51.18 | 317.79 | 66.63 | 2167.38 | 0.85 | 45.79 | 40.94 | 1.76 | 3.12 |
| 5.83 | 13.40 | 11.42 | 72.97 | 139.15 | 55.63 | 312.55 | 65.27 | 2275.76 | 0.87 | 44.77 | 38.72 | 1.80 | 3.25 |
| 5.84 | 10.42 | 12.61 | 47.96 | 80.49 | 54.66 | 202.66 | 52.33 | 1932.52 | 1.04 | 42.43 | 30.70 | 1.84 | 3.67 |
| 5.86 | 12.93 | 10.91 | 54.46 | 147.44 | 45.26 | 231.62 | 56.49 | 1944.18 | 0.98 | 43.87 | 34.91 | 1.85 | 3.55 |
| 5.90 | 11.60 | 7.69 | 56.41 | 99.21 | 47.84 | 245.29 | 59.54 | 1841.25 | 0.89 | 45.17 | 39.11 | 1.80 | 3.30 |
| 5.91 | 13.33 | 9.15 | 67.62 | 112.27 | 64.03 | 313.93 | 71.07 | 2338.01 | 0.86 | 45.32 | 39.82 | 1.82 | 3.51 |
| 5.93 | 12.11 | 9.14 | 66.66 | 127.04 | 55.88 | 300.33 | 57.80 | 1974.09 | 0.84 | 45.74 | 40.87 | 1.72 | 2.88 |
| 5.95 | 14.95 | 10.86 | 72.02 | 147.47 | 60.19 | 324.47 | 73.11 | 2563.87 | 0.89 | 44.73 | 38.25 | 1.84 | 3.73 |
| 5.97 | 15.31 | 11.70 | 65.29 | 154.24 | 51.44 | 339.61 | 71.64 | 2136.50 | 0.93 | 44.22 | 36.66 | 1.86 | 3.37 |
| 5.98 | 14.00 | 10.32 | 53.08 | 93.10 | 45.97 | 194.24 | 69.06 | 2005.07 | 1.05 | 42.50 | 30.60 | 1.91 | 3.86 |
| 6.00 | 14.11 |  | 55.54 | 75.20 | 56.56 | 246.24 | 64.98 | 2273.80 | 1.38 | 35.37 | 5.35 | 2.18 | 5.55 |
| 6.02 | 11.10 | 10.62 | 73.27 | 79.35 | 5.63 | 125.08 |  | 47.94 | 0.90 | 44.41 | 37.58 | 1.72 | 0.07 |
| 6.04 | 16.05 | 0.91 | 63.27 | 78.34 | 50.01 | 213.53 | 2.91 | 470.91 | 1.04 | 42.02 | 29.90 | 1.75 | 0.79 |
| 6.06 | 15.05 |  | 49.18 | 58.30 | 35.25 | 115.03 | 1.27 | 173.77 | 1.28 | 37.89 | 13.57 | 1.94 | 0.40 |
| 6.07 | 12.36 |  | 45.59 | 128.19 | 7.57 | 93.47 | 2.51 | 11.03 | 1.78 | 30.18 | 26.64 | 2.62 | 0.04 |
| 6.09 | 10.37 |  | 53.48 | 148.37 | 54.04 | 302.01 | 67.56 | 2367.82 | 1.09 | 39.92 | 24.61 | 1.95 | 4.86 |
| 6.11 | 9.75 | 7.22 | 65.13 | 120.41 | 44.94 | 288.41 | 31.34 | 1488.90 | 0.94 | 43.44 | 34.95 | 1.82 | 2.52 |
| 6.18 | 11.74 | 7.90 | 62.20 | 105.94 | 22.51 | 143.52 |  | 80.78 | 0.91 | 44.47 | 37.48 | 1.78 | 0.13 |
| 6.20 | 11.65 | 0.08 | 50.50 | 124.35 | 32.61 | 163.87 |  | 110.83 | 1.16 | 39.58 | 21.75 | 1.84 | 0.23 |
| 6.22 | 14.19 |  | 50.43 | 84.19 | 48.09 | 137.12 | 57.90 | 2038.35 | 1.20 | 39.06 | 19.16 | 1.94 | 4.35 |
| 6.25 | 13.38 | 5.87 | 59.96 | 99.60 | 42.94 | 197.67 | 4.98 | 333.72 | 0.91 | 44.23 | 37.11 | 1.83 | 0.59 |
| 6.27 | 13.10 |  | 43.15 | 55.61 | 36.28 | 162.27 | 3.07 | 167.50 | 1.22 | 38.78 | 17.44 | 1.91 | 0.42 |
| 6.29 | 12.03 | 4.82 | 44.37 | 83.93 | 33.46 | 171.24 | 0.83 | 232.66 | 1.03 | 42.23 | 30.52 | 1.96 | 0.57 |
| 6.32 | 16.20 |  | 42.14 | 89.98 | 50.62 | 98.14 | 4.78 | 362.37 | 1.21 | 38.51 | 18.02 | 1.92 | 0.78 |
| 6.37 | 15.00 |  | 38.35 | 120.06 | 31.33 | 127.92 | 1.37 | 211.22 | 1.35 | 36.56 | 7.73 | 2.02 | 0.55 |
