# Supplementary material for: Environmental evolution of a coastal lake in the Larsemann Hills, East Antarctica during the Holocene: a multi-proxy perspective
Source: Sci Rep. 2026 Feb 15;16:9139. doi: 10.1038/s41598-026-39218-8 (PMC12996284; doi:10.1038/s41598-026-39218-8)
Supplement: Supplementary file 4 — Supplementary Material 4 [file 41598_2026_39218_MOESM4_ESM.docx]

Extended Data Table 3: Particle size data and total organic carbon (TOC) data for SL1 sediment core.

| **Age (cal ka BP)** | **Sand (%)** | **Silt (%)** | **Clay (%)** | **Mean Grain Size (microns)** | **EM1 (% Abundance)** | **EM2 (% Abundance)** | **EM3 (% Abundance)** | **EM4 (% Abundance)** |
| --- | --- | --- | --- | --- | --- | --- | --- | --- |
| 0.25 | 61.10 | 34.19 | 4.71 | 82.47 |  |  |  |  |
| 0.54 | 69.87 | 26.02 | 4.11 | 105.03 |  |  |  |  |
| 0.84 | 60.06 | 33.69 | 6.26 | 78.30 | 28.82 | 23.93 | 43.87 | 3.38 |
| 1.14 | 47.07 | 45.38 | 7.55 | 49.32 | 28.76 | 21.24 | 46.23 | 3.77 |
| 1.31 | 88.59 | 9.47 | 1.94 | 228.76 | 28.49 | 15.73 | 46.71 | 9.06 |
| 1.48 | 63.78 | 31.54 | 4.68 | 97.83 | 28.66 | 11.86 | 50.68 | 8.80 |
| 1.65 | 78.07 | 18.20 | 3.73 | 181.82 | 17.93 | 7.64 | 57.79 | 16.64 |
| 1.75 | 62.15 | 31.95 | 5.90 | 93.50 | 17.93 | 3.55 | 48.22 | 30.30 |
| 1.84 | 92.39 | 5.70 | 1.91 | 351.19 | 23.10 | 8.81 | 38.74 | 29.36 |
| 2.07 | 97.03 | 2.29 | 0.69 | 496.70 | 20.87 | 9.48 | 32.87 | 36.78 |
| 2.21 | 36.40 | 51.34 | 12.26 | 27.85 | 17.27 | 9.48 | 23.57 | 49.69 |
| 2.35 | 86.01 | 10.68 | 3.31 | 287.52 | 36.62 | 9.48 | 12.30 | 41.59 |
| 2.52 | 74.30 | 20.67 | 5.03 | 194.39 | 41.83 | 14.92 | 7.91 | 35.34 |
| 2.70 | 14.87 | 68.50 | 16.63 | 14.38 | 30.56 | 9.87 | 8.78 | 50.79 |
| 2.88 | 71.59 | 23.67 | 4.74 | 166.71 | 32.50 | 11.51 | 8.68 | 47.31 |
| 3.07 | 96.36 | 2.85 | 0.79 | 607.53 | 30.91 | 16.53 | 13.66 | 38.91 |
| 3.26 | 76.46 | 19.44 | 4.10 | 182.32 | 22.44 | 20.40 | 17.37 | 39.78 |
| 3.46 | 82.17 | 14.58 | 3.25 | 222.10 | 20.89 | 21.50 | 21.95 | 35.66 |
| 3.56 | 45.61 | 43.51 | 10.88 | 44.15 | 20.01 | 29.57 | 29.55 | 20.87 |
| 3.67 | 73.77 | 21.98 | 4.25 | 145.73 | 19.65 | 33.26 | 35.45 | 11.64 |
| 3.77 | 81.73 | 15.46 | 2.81 | 157.40 | 20.77 | 36.55 | 36.23 | 6.45 |
| 3.83 | 73.67 | 22.38 | 3.95 | 121.37 | 13.98 | 34.71 | 41.64 | 9.67 |
| 3.88 | 68.78 | 26.50 | 4.73 | 95.87 | 19.02 | 33.67 | 42.28 | 5.03 |
| 3.94 | 74.69 | 20.39 | 4.91 | 150.20 | 28.40 | 29.76 | 37.47 | 4.36 |
| 3.99 | 52.76 | 39.42 | 7.81 | 57.19 | 34.10 | 29.70 | 30.39 | 5.81 |
| 4.04 | 48.86 | 42.85 | 8.29 | 48.42 | 42.65 | 27.91 | 22.97 | 6.48 |
| **Age (cal ka BP)** | **Sand (%)** | **Silt (%)** | **Clay (%)** | **Mean Grain Size (microns)** | **EM1 (% Abundance)** | **EM2 (% Abundance)** | **EM3 (% Abundance)** | **EM4 (% Abundance)** |
| 4.10 | 51.99 | 39.13 | 8.88 | 54.55 | 49.05 | 27.47 | 20.78 | 2.69 |
| 4.15 | 41.34 | 50.11 | 8.55 | 38.81 | 40.35 | 21.99 | 33.67 | 3.99 |
| 4.20 | 49.43 | 40.32 | 10.24 | 50.83 | 30.97 | 15.37 | 36.53 | 17.13 |
| 4.24 | 95.38 | 3.56 | 1.06 | 325.00 | 25.81 | 11.50 | 46.77 | 15.92 |
| 4.30 | 97.39 | 2.06 | 0.54 | 482.98 | 25.80 | 9.20 | 49.75 | 15.26 |
| 4.35 | 76.15 | 19.76 | 4.09 | 140.55 | 15.29 | 19.15 | 50.62 | 14.94 |
| 4.40 | 42.44 | 47.61 | 9.95 | 40.20 | 20.58 | 31.74 | 34.30 | 13.37 |
| 4.45 | 82.62 | 14.99 | 2.39 | 141.92 | 21.11 | 42.61 | 36.04 | 0.24 |
| 4.50 | 60.64 | 35.09 | 4.27 | 70.98 | 30.14 | 45.83 | 24.02 | 0.00 |
| 4.55 | 76.73 | 20.02 | 3.25 | 123.06 | 20.61 | 58.19 | 21.20 | 0.00 |
| 4.60 | 38.43 | 53.31 | 8.26 | 36.28 | 25.50 | 58.59 | 15.91 | 0.00 |
| 4.65 | 65.95 | 28.41 | 5.64 | 68.05 | 30.37 | 53.27 | 16.36 | 0.00 |
| 4.70 | 59.65 | 32.69 | 7.65 | 59.28 | 34.71 | 45.04 | 19.98 | 0.28 |
| 4.74 | 46.90 | 45.42 | 7.68 | 46.85 | 31.41 | 48.58 | 19.74 | 0.28 |
| 4.78 | 71.67 | 23.25 | 5.08 | 122.92 | 42.31 | 37.26 | 20.15 | 0.28 |
| 4.82 | 47.06 | 45.15 | 7.79 | 42.87 | 47.45 | 33.53 | 17.00 | 2.02 |
| 4.86 | 35.80 | 52.84 | 11.36 | 30.82 | 53.77 | 29.79 | 14.43 | 2.02 |
| 4.89 | 46.93 | 45.27 | 7.80 | 50.31 | 54.92 | 29.71 | 6.16 | 9.21 |
| 4.92 | 26.01 | 63.14 | 10.85 | 23.99 | 47.23 | 21.75 | 11.66 | 19.36 |
| 4.95 | 68.00 | 27.87 | 4.12 | 136.70 | 45.98 | 20.75 | 13.91 | 19.36 |
| 4.98 | 86.77 | 11.56 | 1.67 | 297.34 | 46.70 | 14.31 | 15.95 | 23.04 |
| 5.02 | 41.75 | 48.02 | 10.22 | 40.38 | 33.14 | 15.35 | 15.95 | 35.56 |
| 5.05 | 51.05 | 39.96 | 8.99 | 69.33 | 45.30 | 14.02 | 12.59 | 28.09 |
| 5.08 | 86.68 | 10.87 | 2.44 | 386.77 | 50.90 | 14.10 | 12.41 | 22.59 |
| 5.11 | 20.58 | 63.65 | 15.77 | 16.93 | 57.60 | 9.86 | 9.96 | 22.59 |
| 5.14 | 63.63 | 30.69 | 5.68 | 106.24 | 50.05 | 12.97 | 11.53 | 25.44 |
| 5.17 | 17.60 | 67.52 | 14.88 | 16.88 | 56.69 | 17.57 | 12.81 | 12.92 |
| **Age (cal ka BP)** | **Sand (%)** | **Silt (%)** | **Clay (%)** | **Mean Grain Size (microns)** | **EM1 (% Abundance)** | **EM2 (% Abundance)** | **EM3 (% Abundance)** | **EM4 (% Abundance)** |
| 5.20 | 78.88 | 18.78 | 2.33 | 214.83 | 40.37 | 22.14 | 16.27 | 21.21 |
| 5.23 | 47.38 | 46.31 | 6.31 | 48.03 | 49.92 | 20.28 | 11.96 | 17.84 |
| 5.26 | 83.75 | 13.76 | 2.50 | 250.93 | 32.53 | 20.93 | 20.76 | 25.78 |
| 5.30 | 28.96 | 59.19 | 11.86 | 27.06 | 33.87 | 23.59 | 21.56 | 20.98 |
| 5.33 | 87.97 | 10.46 | 1.57 | 291.22 | 43.12 | 14.57 | 21.33 | 20.98 |
| 5.36 | 68.19 | 28.67 | 3.15 | 120.07 | 50.26 | 12.75 | 20.33 | 16.66 |
| 5.39 | 18.79 | 64.72 | 16.49 | 17.57 | 47.52 | 18.48 | 18.61 | 15.39 |
| 5.41 | 54.67 | 38.00 | 7.34 | 73.66 | 49.86 | 28.30 | 11.40 | 10.44 |
| 5.44 | 34.09 | 56.70 | 9.21 | 31.61 | 53.47 | 31.16 | 8.40 | 6.97 |
| 5.46 | 68.01 | 27.18 | 4.81 | 96.78 | 39.39 | 33.24 | 13.67 | 13.70 |
| 5.48 | 46.96 | 43.66 | 9.37 | 40.29 | 44.17 | 35.48 | 10.63 | 9.72 |
| 5.50 | 72.70 | 22.34 | 4.95 | 154.35 | 40.94 | 38.71 | 10.63 | 9.72 |
| 5.51 | 33.22 | 58.20 | 8.58 | 33.37 | 52.61 | 32.85 | 7.75 | 6.79 |
| 5.55 | 43.04 | 48.03 | 8.94 | 36.90 | 55.78 | 31.09 | 6.33 | 6.79 |
| 5.57 | 30.39 | 59.99 | 9.62 | 28.38 | 65.50 | 34.16 | 0.00 | 0.34 |
| 5.59 | 41.21 | 50.96 | 7.84 | 36.88 | 63.13 | 36.53 | 0.00 | 0.34 |
| 5.61 | 33.87 | 57.54 | 8.59 | 32.96 | 63.70 | 34.76 | 1.20 | 0.34 |
| 5.62 | 39.75 | 52.23 | 8.02 | 36.01 | 60.58 | 37.48 | 1.66 | 0.28 |
| 5.64 | 42.99 | 49.93 | 7.08 | 41.97 | 50.89 | 40.25 | 8.57 | 0.28 |
| 5.66 | 39.39 | 52.40 | 8.21 | 36.49 | 42.06 | 43.66 | 14.28 | 0.00 |
| 5.68 | 71.75 | 23.89 | 4.36 | 98.35 | 42.02 | 43.70 | 14.28 | 0.00 |
| 5.71 | 62.27 | 32.67 | 5.06 | 77.19 | 45.57 | 40.95 | 13.08 | 0.40 |
| 5.73 | 39.76 | 52.23 | 8.01 | 36.75 | 49.35 | 37.63 | 12.62 | 0.40 |
| 5.75 | 33.65 | 58.56 | 7.80 | 34.34 | 65.11 | 28.72 | 5.78 | 0.40 |
| 5.77 | 28.66 | 63.52 | 7.82 | 30.07 | 67.23 | 27.02 | 5.35 | 0.40 |
| 5.78 | 24.49 | 63.90 | 11.61 | 23.18 | 66.08 | 22.59 | 10.34 | 0.98 |
| 5.80 | 55.01 | 38.42 | 6.56 | 61.87 | 65.06 | 22.33 | 12.02 | 0.58 |
| 5.82 | 50.17 | 41.97 | 7.86 | 56.65 | 59.21 | 21.98 | 16.14 | 2.67 |
| **Age (cal ka BP)** | **Sand (%)** | **Silt (%)** | **Clay (%)** | **Mean Grain Size (microns)** | **EM1 (% Abundance)** | **EM2 (% Abundance)** | **EM3 (% Abundance)** | **EM4 (% Abundance)** |
| 5.84 | 36.56 | 54.83 | 8.61 | 34.68 | 49.15 | 24.85 | 19.90 | 6.10 |
| 5.85 | 51.94 | 41.86 | 6.20 | 66.77 | 55.58 | 23.50 | 14.82 | 6.10 |
| 5.87 | 60.27 | 34.46 | 5.27 | 88.28 | 61.59 | 22.54 | 10.05 | 5.82 |
| 5.89 | 33.20 | 58.69 | 8.11 | 31.56 | 52.40 | 29.26 | 11.99 | 6.36 |
| 5.91 | 29.04 | 61.17 | 9.79 | 28.17 | 42.46 | 42.85 | 10.42 | 4.27 |
| 5.92 | 63.92 | 31.75 | 4.33 | 80.19 | 50.64 | 40.90 | 6.78 | 1.69 |
| 5.94 | 72.65 | 23.80 | 3.55 | 87.94 | 49.02 | 39.01 | 8.47 | 3.50 |
| 5.98 | 31.51 | 58.84 | 9.66 | 30.70 | 33.68 | 42.05 | 21.07 | 3.20 |
| 5.99 | 42.37 | 49.74 | 7.89 | 45.55 | 43.22 | 36.59 | 17.53 | 2.66 |
| 6.01 | 80.24 | 16.71 | 3.05 | 148.20 | 62.74 | 19.34 | 15.26 | 2.66 |
| 6.03 | 34.67 | 57.79 | 7.53 | 33.61 | 62.33 | 17.73 | 16.10 | 3.84 |
| 6.05 | 17.87 | 68.71 | 13.42 | 18.48 | 56.96 | 19.69 | 19.73 | 3.62 |
| 6.06 | 34.10 | 55.72 | 10.19 | 35.32 | 61.28 | 25.86 | 9.24 | 3.62 |
| 6.08 | 60.77 | 34.26 | 4.97 | 83.03 | 60.67 | 24.96 | 10.02 | 4.35 |
| 6.10 | 59.56 | 35.37 | 5.06 | 63.08 | 49.05 | 32.74 | 12.69 | 5.52 |
| 6.12 | 37.41 | 52.95 | 9.63 | 35.99 | 40.57 | 35.65 | 14.16 | 9.62 |
| 6.14 | 54.74 | 39.69 | 5.57 | 64.82 | 41.87 | 38.09 | 11.14 | 8.89 |
| 6.16 | 63.51 | 31.89 | 4.60 | 113.78 | 51.96 | 30.35 | 8.79 | 8.89 |
| 6.18 | 52.44 | 41.31 | 6.26 | 56.87 | 44.77 | 31.67 | 12.80 | 10.77 |
| 6.20 | 29.47 | 61.40 | 9.14 | 27.97 | 50.48 | 28.09 | 11.83 | 9.60 |
| 6.22 | 62.51 | 32.45 | 5.04 | 90.84 | 61.66 | 25.54 | 9.33 | 3.47 |
| 6.25 | 36.64 | 55.79 | 7.57 | 36.10 | 65.72 | 23.93 | 7.10 | 3.25 |
| 6.27 | 23.22 | 65.79 | 10.99 | 22.43 | 64.55 | 24.26 | 7.61 | 3.57 |
| 6.29 | 39.12 | 52.74 | 8.14 | 37.46 | 72.26 | 21.30 | 5.48 | 0.97 |
| 6.31 | 34.20 | 56.23 | 9.57 | 31.63 | 73.06 | 21.36 | 4.36 | 1.21 |
| 6.33 | 36.74 | 54.88 | 8.38 | 36.86 | 67.76 | 24.81 | 5.82 | 1.61 |

| **Age (cal ka BP)** | **TOC (%)** |
| --- | --- |
| 0.25 | 21.49 |
| 0.54 | 20.68 |
| 0.84 | 23.04 |
| 1.14 | 20.68 |
| 1.31 | 13.75 |
| 1.48 | 4.49 |
| 1.65 | 3.76 |
| 1.75 | 1.46 |
| 1.84 | 1.17 |
| 2.07 | 0.40 |
| 2.21 | 0.26 |
| 2.35 | 0.56 |
| 2.52 | 0.27 |
| 2.70 | 0.32 |
| 2.88 | 0.20 |
| 3.07 | 0.19 |
| 3.26 | 0.41 |
| 3.46 | 0.84 |
| 3.67 | 0.63 |
| 3.83 | 0.84 |
| 3.94 | 0.42 |
| 4.04 | 1.14 |
| 4.15 | 1.29 |
| 4.24 | 0.32 |
| 4.35 | 0.34 |
| 4.45 | 0.91 |
| 4.55 | 1.04 |
| 4.65 | 0.69 |
| 4.74 | 0.61 |
| 4.82 | 0.65 |
| 4.89 | 0.94 |
| 4.95 | 0.71 |
| 5.02 | 0.41 |
| 5.08 | 0.38 |
| 5.14 | 0.44 |
| 5.20 | 0.54 |
| 5.26 | 0.74 |
| 5.33 | 0.78 |
| 5.39 | 1.02 |
| 5.44 | 1.18 |
| 5.48 | 1.06 |
| 5.51 | 0.96 |
| 5.55 | 1.05 |
| 5.59 | 1.36 |
| 5.62 | 1.67 |
| **Age (cal ka BP)** | **TOC (%)** |
| 5.66 | 1.49 |
| 5.70 | 1.64 |
| 5.73 | 1.48 |
| 5.77 | 2.24 |
| 5.80 | 1.11 |
| 5.84 | 1.90 |
| 5.87 | 1.69 |
| 5.91 | 1.82 |
| 5.94 | 1.30 |
| 5.98 | 2.03 |
| 6.01 | 1.23 |
| 6.05 | 1.31 |
| 6.08 | 1.34 |
| 6.12 | 1.16 |
| 6.16 | 1.57 |
| 6.20 | 1.34 |
| 6.25 | 1.79 |
| 6.29 | 2.21 |
| 6.31 | 1.69 |
| 6.37 | 1.51 |
