# Supplementary material for: Environmental evolution of a coastal lake in the Larsemann Hills, East Antarctica during the Holocene: a multi-proxy perspective
Source: Sci Rep. 2026 Feb 15;16:9139. doi: 10.1038/s41598-026-39218-8 (PMC12996284; doi:10.1038/s41598-026-39218-8)
Supplement: Supplementary file 5 — Supplementary Material 5 [file 41598_2026_39218_MOESM5_ESM.docx]

Extended Data Table 4: Percentage rounded quartz grain data for SL1 sediment core.

| **Age (cal ka BP)** | **Rounded Grains (%)** |
| --- | --- |
| 0.54 | 19.25 |
| 1.14 | 10.25 |
| 1.48 | 13.25 |
| 1.75 | 13.25 |
| 1.84 | 11.90 |
| 2.07 | 12.25 |
| 2.35 | 13.71 |
| 2.70 | 13.65 |
| 3.07 | 19.25 |
| 3.46 | 16.01 |
| 3.67 | 20.75 |
| 3.83 | 14.50 |
| 3.94 | 11.00 |
| 4.04 | 20.50 |
| 4.15 | 17.50 |
| 4.24 | 12.25 |
| 4.35 | 14.00 |
| 4.45 | 11.75 |
| 4.55 | 13.25 |
| 4.65 | 18.00 |
| 4.74 | 15.50 |
| 4.82 | 20.75 |
| 4.89 | 16.00 |
| 4.95 | 18.00 |
| 5.02 | 18.50 |
| 5.08 | 17.25 |
| 5.14 | 13.50 |
| 5.20 | 20.50 |
| 5.26 | 18.50 |
| 5.33 | 10.50 |
| 5.39 | 15.25 |
| 5.44 | 12.25 |
| 5.48 | 20.75 |
| 5.51 | 13.50 |
| 5.55 | 16.00 |
| 5.59 | 13.50 |
| 5.62 | 20.75 |
| 5.66 | 27.25 |
| 5.70 | 18.00 |
| 5.73 | 15.25 |
| 5.77 | 24.25 |
| 5.80 | 21.25 |
| 5.84 | 19.50 |
| 5.87 | 19.25 |
| 5.91 | 20.75 |
| **Age (cal ka BP)** | **Rounded Grains (%)** |
|  |  |
| 5.94 | 24.50 |
| 5.98 | 27.25 |
| 6.01 | 19.50 |
| 6.05 | 20.50 |
| 6.08 | 23.50 |
| 6.12 | 15.00 |
| 6.16 | 17.25 |
| 6.20 | 14.00 |
| 6.25 | 26.75 |
| 6.29 | 29.50 |
| 6.31 | 28.00 |
| 6.37 | 26.50 |
